# The Comet Interceptor Mission

**DOI:** 10.1007/s11214-023-01035-0

**Published:** 2024-01-24

**Authors:** Geraint H. Jones, Colin Snodgrass, Cecilia Tubiana, Michael Küppers, Hideyo Kawakita, Luisa M. Lara, Jessica Agarwal, Nicolas André, Nicholas Attree, Uli Auster, Stefano Bagnulo, Michele Bannister, Arnaud Beth, Neil Bowles, Andrew Coates, Luigi Colangeli, Carlos Corral van Damme, Vania Da Deppo, Johan De Keyser, Vincenzo Della Corte, Niklas Edberg, Mohamed Ramy El-Maarry, Sara Faggi, Marco Fulle, Ryu Funase, Marina Galand, Charlotte Goetz, Olivier Groussin, Aurélie Guilbert-Lepoutre, Pierre Henri, Satoshi Kasahara, Akos Kereszturi, Mark Kidger, Matthew Knight, Rosita Kokotanekova, Ivana Kolmasova, Konrad Kossacki, Ekkehard Kührt, Yuna Kwon, Fiorangela La Forgia, Anny-Chantal Levasseur-Regourd, Manuela Lippi, Andrea Longobardo, Raphael Marschall, Marek Morawski, Olga Muñoz, Antti Näsilä, Hans Nilsson, Cyrielle Opitom, Mihkel Pajusalu, Antoine Pommerol, Lubomir Prech, Nicola Rando, Francesco Ratti, Hanna Rothkaehl, Alessandra Rotundi, Martin Rubin, Naoya Sakatani, Joan Pau Sánchez, Cyril Simon Wedlund, Anamarija Stankov, Nicolas Thomas, Imre Toth, Geronimo Villanueva, Jean-Baptiste Vincent, Martin Volwerk, Peter Wurz, Arno Wielders, Kazuo Yoshioka, Konrad Aleksiejuk, Fernando Alvarez, Carine Amoros, Shahid Aslam, Barbara Atamaniuk, Jędrzej Baran, Tomasz Barciński, Thomas Beck, Thomas Behnke, Martin Berglund, Ivano Bertini, Marcin Bieda, Piotr Binczyk, Martin-Diego Busch, Andrei Cacovean, Maria Teresa Capria, Chris Carr, José María Castro Marín, Matteo Ceriotti, Paolo Chioetto, Agata Chuchra-Konrad, Lorenzo Cocola, Fabrice Colin, Chiaki Crews, Victoria Cripps, Emanuele Cupido, Alberto Dassatti, Björn J. R. Davidsson, Thierry De Roche, Jan Deca, Simone Del Togno, Frederik Dhooghe, Kerri Donaldson Hanna, Anders Eriksson, Andrey Fedorov, Estela Fernández-Valenzuela, Stefano Ferretti, Johan Floriot, Fabio Frassetto, Jesper Fredriksson, Philippe Garnier, Dorota Gaweł, Vincent Génot, Thomas Gerber, Karl-Heinz Glassmeier, Mikael Granvik, Benjamin Grison, Herbert Gunell, Tedjani Hachemi, Christian Hagen, Rajkumar Hajra, Yuki Harada, Johann Hasiba, Nico Haslebacher, Miguel Luis Herranz De La Revilla, Daniel Hestroffer, Tilak Hewagama, Carrie Holt, Stubbe Hviid, Iaroslav Iakubivskyi, Laura Inno, Patrick Irwin, Stavro Ivanovski, Jiri Jansky, Irmgard Jernej, Harald Jeszenszky, Jaime Jimenéz, Laurent Jorda, Mihkel Kama, Shingo Kameda, Michael S. P. Kelley, Kamil Klepacki, Tomáš Kohout, Hirotsugu Kojima, Tomasz Kowalski, Masaki Kuwabara, Michal Ladno, Gunter Laky, Helmut Lammer, Radek Lan, Benoit Lavraud, Monica Lazzarin, Olivier Le Duff, Qiu-Mei Lee, Cezary Lesniak, Zoe Lewis, Zhong-Yi Lin, Tim Lister, Stephen Lowry, Werner Magnes, Johannes Markkanen, Ignacio Martinez Navajas, Zita Martins, Ayako Matsuoka, Barbara Matyjasiak, Christian Mazelle, Elena Mazzotta Epifani, Mirko Meier, Harald Michaelis, Marco Micheli, Alessandra Migliorini, Aude-Lyse Millet, Fernando Moreno, Stefano Mottola, Bruno Moutounaick, Karri Muinonen, Daniel R. Müller, Go Murakami, Naofumi Murata, Kamil Myszka, Shintaro Nakajima, Zoltan Nemeth, Artiom Nikolajev, Simone Nordera, Dan Ohlsson, Aire Olesk, Harald Ottacher, Naoya Ozaki, Christophe Oziol, Manish Patel, Aditya Savio Paul, Antti Penttilä, Claudio Pernechele, Joakim Peterson, Enrico Petraglio, Alice Maria Piccirillo, Ferdinand Plaschke, Szymon Polak, Frank Postberg, Herman Proosa, Silvia Protopapa, Walter Puccio, Sylvain Ranvier, Sean Raymond, Ingo Richter, Martin Rieder, Roberto Rigamonti, Irene Ruiz Rodriguez, Ondrej Santolik, Takahiro Sasaki, Rolf Schrödter, Katherine Shirley, Andris Slavinskis, Balint Sodor, Jan Soucek, Peter Stephenson, Linus Stöckli, Paweł Szewczyk, Gabor Troznai, Ludek Uhlir, Naoto Usami, Aris Valavanoglou, Jakub Vaverka, Wei Wang, Xiao-Dong Wang, Gaëtan Wattieaux, Martin Wieser, Sebastian Wolf, Hajime Yano, Ichiro Yoshikawa, Vladimir Zakharov, Tomasz Zawistowski, Paola Zuppella, Giovanna Rinaldi, Hantao Ji

**Affiliations:** 1 Mullard Space Science Laboratory, University College London, Holmbury St. Mary, Dorking, UK; 2 The Centre for Planetary Sciences at UCL/Birkbeck, London, UK; 3 The University of Edinburgh, Edinburgh, UK; 4 INAF, IAPS, Rome, Italy; 5 European Space Agency (ESA), European Space Astronomy Centre (ESAC), Madrid, Spain; 6 Koyama Astronomical Observatory, Kyoto Sangyo University, Kyoto, Japan; 7 Instituto de Astrofisica de Andalucía – CSIC, Granada, Spain; 8 Institut für Geophysik und extraterrestrische Physik, Technische Universität Braunschweig, Braunschweig, Germany; 9 IRAP, CNRS, University Toulouse 3, CNES, Toulouse, France; 10 Armagh Observatory and Planetarium, Armagh, UK; 11 University of Canterbury, Christchurch, New Zealand; 12 Umeå University, Umeå, Sweden; 13 Department of Physics, University of Oxford, Oxford, UK; 14 European Space Agency, ESTEC, Noordwijk, The Netherlands; 15 CNR-Institute for Photonics and Nanotechnologies, Padova, Italy; 16 Royal Belgian Institute of Space Aeronomy, Brussels, Belgium; 17 Swedish Institute of Space Physics, Uppsala/Kiruna, Sweden; 18 Space and Planetary Science Center and Department of Earth Sciences, Khalifa University, Abu Dhabi, United Arab Emirates; 19 NASA Goddard Space Flight Center, Greenbelt, USA; 20 INAF – Osservatorio Astronomico di Trieste, Trieste, Italy; 21 Institute of Space and Astronautical Science, Japan Aerospace Exploration Agency, Kanagawa, Japan; 22 Department of Physics, Imperial College London, London, UK; 23 LGL-TPE, CNRS, ENS, Université Lyon1, UJM, Lyon, France; 24 Laboratoire Lagrange, CNRS, OCA, Université Côte d’Azur, and LPC2E, CNRS, Université d’Orléans, CNES, Orléans, France; 25 The University of Tokyo, Tokyo, Japan; 26 Konkoly Astronomical Institute, Research Centre for Astronomy and Earth Sciences, HUN-REN, Budapest, Hungary; 27 U.S. Naval Academy, Annapolis, USA; 28 Institute of Astronomy and National Astronomical Observatory, Bulgarian Academy of Sciences, Sofia, Bulgaria; 29 Institute of Atmospheric Physics of the Czech Academy of Sciences, Prague, Czech Republic; 30 Faculty of Physics, University of Warsaw, Warsaw, Poland; 31 DLR, Institute of Optical Sensor Systems, Berlin, Germany; 32 Department of Physics and Astronomy, University of Padova, Padova, Italy; 33 LATMOS, Sorbonne Université, CNRS, CNES, Paris, France; 34 CNRS, Laboratoire J.-L. Lagrange, Observatoire de la Côte d’Azur, Nice, France; 35 Space Research Centre of the Polish Academy of Sciences, Warsaw, Poland; 36 VTT Technical Research Centre of Finland Ltd, Espoo, Finland; 37 Tartu Observatory, University of Tartu, Tartu, Estonia; 38 Space Research and Planetary Sciences, Physics Institute, University of Bern, Bern, Switzerland; 39 Charles University, Prague, Czech Republic; 40 Dipartimento di Scienze e Tecnologie, Università degli Studi di Napoli “Parthenope”, Napoli, Italy; 41 Institut Supérieur de l’Aéronautique et de l’Espace, Toulouse, France; 42 Austrian Academy of Sciences, Space Research Institute, Graz, Austria; 43 DLR Institute of Planetary Research, Berlin, Germany; 44 Creotech Instruments, Piaseczno, Poland; 45 University of Glasgow, Glasgow, UK; 46 LPC2E, CNRS, Université d’Orléans, CNES, Orléans, France; 47 The Open University, Milton Keynes, UK; 48 REDS, School of Management and Engineering Vaud, HES-SO University of Applied Sciences and Arts Western Switzerland, Delémont, Switzerland; 49 Jet Propulsion Laboratory, California Institute of Technology, Pasadena, USA; 50 Laboratory for Atmospheric and Space Physics, University of Colorado Boulder, Boulder, USA; 51 Department of Physics, University of Central Florida, Orlando, USA; 52 Florida Space Institute, University of Central Florida, Orlando, USA; 53 Laboratoire d’Astrophysique de Marseille, Aix-Marseille Université, CNRS, Marseille, France; 54 Department of Physics, University of Helsinki, Helsinki, Finland; 55 Asteroid Engineering Lab, Luleå University of Technology, Kiruna, Sweden; 56 Indian Institute of Technology Indore, Indore, India; 57 Kyoto University, Kyoto, Japan; 58 IMCCE, Paris Observatory, Université PSL, CNRS, Sorbonne Université, Univ. Lille, Paris, France; 59 Department of Astronomy, University of Maryland, College Park, USA; 60 University College London, London, UK; 61 College of Science, Rikkyo University, Tokyo, Japan; 62 Department of Geosciences and Geography, University of Helsinki, Helsinki, Finland; 63 Institute of Geology of the Czech Academy of Sciences, Prague, Czech Republic; 64 Research Institute for Sustainable Humanosphere, Kyoto University, Kyoto, Japan; 65 Laboratoire d’astrophysique de Bordeaux, Univ. Bordeaux, CNRS, Nouvelle-Aquitaine, France; 66 Institute of Astronomy, National Central University, Taoyuan, Taiwan; 67 Las Cumbres Observatory, Goleta, USA; 68 University of Kent, Kent, UK; 69 Centro de Química Estrutural, Institute of Molecular Sciences and Department of Chemical Engineering, Instituto Superior Técnico, Universidade de Lisboa, Lisbon, Portugal; 70 INAF – Osservatorio Astronomico di Roma, Rome, Italy; 71 ESA NEO Coordination Centre, Frascati, Italy; 72 Wigner Research Centre for Physics, Budapest, Hungary; 73 INAF-Osservatorio Astronomico di Padova, Padova, Italy; 74 Freie Universitaet Berlin, Berlin, Germany; 75 Southwest Research Institute, Boulder, USA; 76 SGF Ltd., Egyed, Hungary; 77 Laboratoire Plasma et Conversion d’Energie (LAPLACE), CNRS, Université de Toulouse 3, Toulouse, France; 78 LESIA, Observatoire de Paris, Université PSL, Sorbonne Université, Université Paris Cité, CNRS, Paris, France; 79 Caltech/IPAC, 1200 E California Blvd, MC 100-22 Pasadena, CA 91125, USA; 80 Department of Astrophysical Sciences, Princeton University, Princeton, USA

**Keywords:** Comets, Spacecraft, Instruments – spaceborne and space research

## Abstract

Here we describe the novel, multi-point Comet Interceptor mission. It is dedicated to the exploration of a little-processed long-period comet, possibly entering the inner Solar System for the first time, or to encounter an interstellar object originating at another star. The objectives of the mission are to address the following questions: What are the surface composition, shape, morphology, and structure of the target object? What is the composition of the gas and dust in the coma, its connection to the nucleus, and the nature of its interaction with the solar wind? The mission was proposed to the European Space Agency in 2018, and formally adopted by the agency in June 2022, for launch in 2029 together with the Ariel mission. Comet Interceptor will take advantage of the opportunity presented by ESA’s F-Class call for fast, flexible, low-cost missions to which it was proposed. The call required a launch to a halo orbit around the Sun-Earth L2 point. The mission can take advantage of this placement to wait for the discovery of a suitable comet reachable with its minimum $\varDelta $V capability of $600\text{ ms}^{-1}$. Comet Interceptor will be unique in encountering and studying, at a nominal closest approach distance of 1000 km, a comet that represents a near-pristine sample of material from the formation of the Solar System. It will also add a capability that no previous cometary mission has had, which is to deploy two sub-probes – B1, provided by the Japanese space agency, JAXA, and B2 – that will follow different trajectories through the coma. While the main probe passes at a nominal 1000 km distance, probes B1 and B2 will follow different chords through the coma at distances of 850 km and 400 km, respectively. The result will be unique, simultaneous, spatially resolved information of the 3-dimensional properties of the target comet and its interaction with the space environment. We present the mission’s science background leading to these objectives, as well as an overview of the scientific instruments, mission design, and schedule.

## Introduction

Comets are the surviving remnants of the original building blocks of the Solar System. A significant amount of pristine material from the formation of the Solar System persists in the Oort Cloud (OC), which extends out to at least 1 light year from the Sun, unmodified or barely modified since the earliest days of the Solar System. All other material to which we have access – asteroids, meteorites, lunar and planetary surface samples and atmospheres – has been significantly or heavily modified, both physically and chemically since its formation. While multiple comets have been studied *in situ*, all, with the exception of 1P/Halley, have arguably been low-activity, short-period, highly-evolved comets, which have changed radically since their formation, having spent considerable time in the inner Solar System. Even Comet Halley, with its longer period and high activity is thought to have made several thousand returns to perihelion. Such objects are highly depleted in volatiles (A’Hearn et al. 1995), particularly low-temperature volatiles, at least in their outer layers. While all the comets studied *in situ* to date share certain characteristics (e.g., low albedo, jet activity, …), the observed nucleus morphology shows considerable differences between objects, suggesting that they have experienced radically different evolutionary processes, possibly due, at least in part, to their orbital instability that leads to considerable variations of perihelion distance and thus insolation over timescales of decades and centuries. These highly evolved objects also show significant morphological differences with the only small Kuiper-Belt object to be observed to date, 2014 MU_69_ (Arrokoth), studied by the New Horizons mission during a ∼3540 km flyby at a relative speed of 14.3 km s^−1^, around 3 times further and likely slower than the proposed Comet Interceptor encounter.

Dynamically new comets arriving from the OC have, by definition, never visited the inner Solar System before. They are expected to be rich in the low-temperature volatiles retained from their formation and close to their pristine state, particularly in the case of a pre-perihelion encounter. Given the long lead-up times required to plan and launch a space mission, it has been impossible prior to Comet Interceptor to contemplate encounter missions with such objects, to observe a pristine, or minimally evolved nucleus and study its morphology, its activity, and its interactions with the interplanetary medium. The opportunity to observe, *in situ*, a dynamically new comet that is entering the inner Solar System for the first time will allow the data from previous comet encounter missions and from ground-based campaigns to be placed in its proper context. Such an encounter will allow the composition, both chemical and isotopic, of the protosolar nebula to be studied. This will offer valuable insights into the chemical and isotopic evolution of the Solar System since its formation.

It has been known for many years that there is a population of dynamically new objects that are lost to the Solar System. These objects’ paths are perturbed in the inner Solar System into hyperbolic orbits and, thus, escape solar influence. It has long been assumed that there is a similar population of objects external to the Solar System that enter it from interstellar space: these objects have been expelled from the stellar system where they formed and thus present the opportunity of sampling primordial material formed around other stars, in different conditions to the formation of our Solar System. Such an interstellar wanderer manifests itself by having a sufficiently large hyperbolic excess of velocity to demonstrate that it could not have originated in the OC. Two such objects are now known: 1I/’Oumuamua and 2I/Borisov, discovered in 2017 and 2019 respectively. While the probability of detecting such an interstellar object that satisfies the targeting conditions for Comet Interceptor is low, it is non-zero: such an object would be potentially an extremely high-value target scientifically. It is expected that the Vera C. Rubin Observatory’s Legacy Survey of Space and Time (LSST) will increase considerably the detection capability, not just of dynamically new objects, but also of this population of interstellar comets.

Comet Interceptor was proposed to the European Space Agency by an international consortium of scientists and engineers, in response to a call for mission proposals issued by ESA in July 2018. The project was selected in June 2019 as the prime candidate for the agency’s first F-class mission in its Cosmic Vision Programme. Phase A started in 2020, with two parallel industrial studies. In April 2021, Comet Interceptor moved into Phase B1. During Phase A and B1, the Science Study Team and Science Steering Committee studied and validated the science case, supported by the ESA study team and members of the proposal consortium, organized into working groups. The mission was formally adopted by ESA in June 2022.

**Fig. 1:**
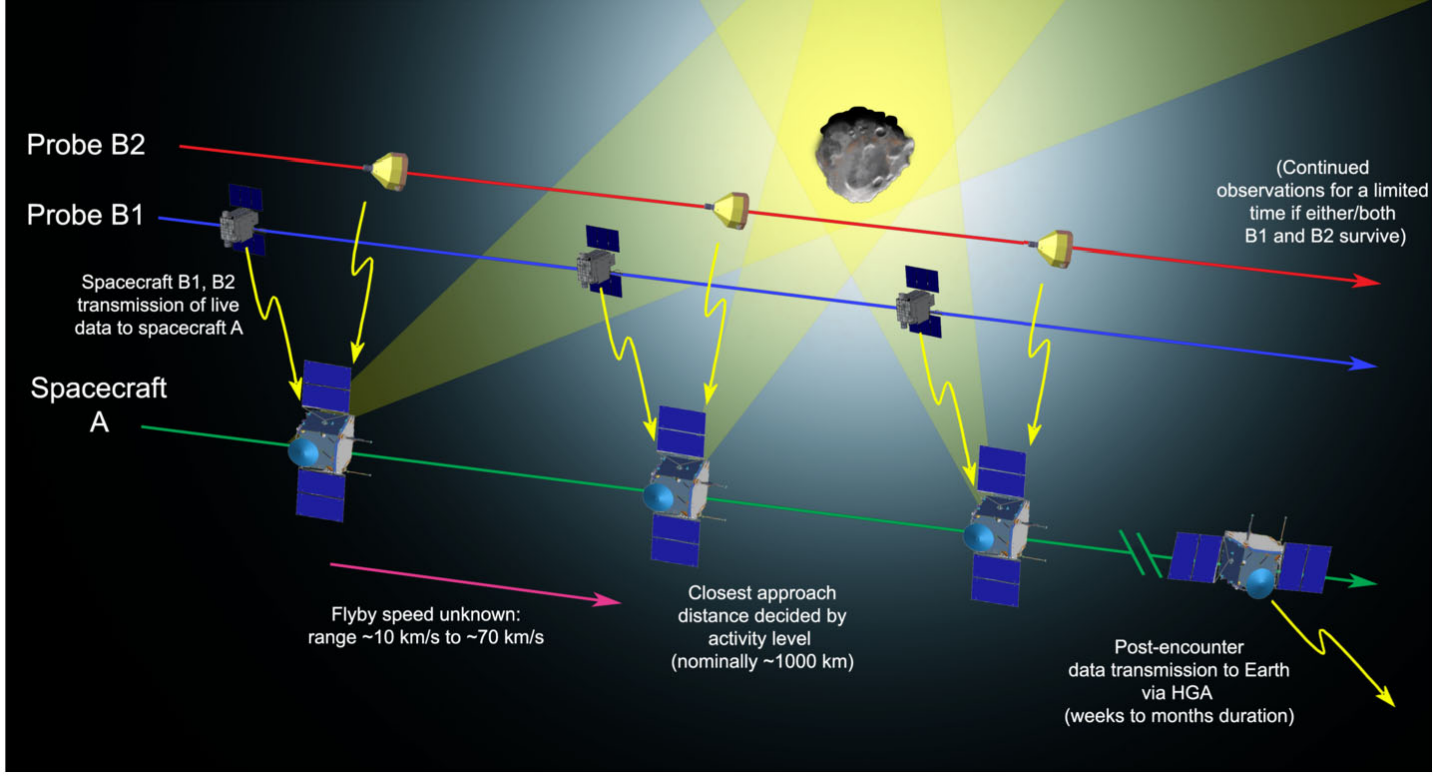
Sketch of the Comet Interceptor flyby, not to scale. Spacecraft A will pass furthest from the nucleus, with probes B1 and B2 passing closer. Both probes will relay their data in real time to be stored on spacecraft A for later transmission

The mission aims to intercept a long-period comet, LPC, ideally a dynamically-new comet, DNC, one of a subset of LPCs that is approaching the inner Solar System for the first time, or even an interstellar body. The intercept will involve a close-approach flyby scenario using three elements: a mother spacecraft, spacecraft A, and smaller probes named B1 and B2 that are carried as payloads until the flyby, and delivered to different flyby trajectories. This will allow the gathering of remote and *in situ* multi-point observations of the comet and its coma (Fig. 1).

Comet Interceptor follows the successful history of European exploration of comets, following in the footsteps of ESA’s Giotto and Rosetta missions, as well as the substantial contribution of scientists from European countries to the two Soviet-led VEGA missions. A preliminary description of the mission as proposed to ESA was published by Snodgrass and Jones (2019). In this work, we describe the scientific rationale for the mission, as well as the mission itself, in much greater depth. This includes a description of the project’s mature payload and mission design as they stand shortly after mission adoption.

## Scientific Context

### Introduction

While the Giotto flyby of 1P/Halley was the first mission to provide good resolution images of a cometary nucleus, the Rosetta rendezvous was conceived, e.g. Glassmeier et al. (2007), as the first mission to monitor the changing activity of a cometary nucleus before and after perihelion, which it achieved (Taylor et al. 2017). Both missions revolutionized cometary science. Rosetta showed that the composition of comet 67P/Churyumov-Gerasimenko (referred to as 67P) is rich in organics and has a different water isotope ratio to Earth’s (Altwegg et al. 2015); that its surface structure and morphology (Sierks et al. 2015) are controlled by seasonal activity (Hässig et al. 2015), with starkly contrasting areas dominated by erosion and by dust fall-back (El-Maarry et al. 2015, 2016); and that the comet’s inner coma is highly dynamic, changing in time and space, e.g., Della Corte et al. (2015), Feldman et al. (2015), Lee et al. (2015). Rosetta also raised important new questions:

- What properties are primordial, and reflect the process of comet formation, and what are evolutionary features?
- How do these properties control cometary activity?
- Are the differences in composition seen in the coma and solar wind interaction spatial or temporal in origin?

We detail these problems in the following Sects. 2.2 and 2.3, divided into two themes that address Nucleus and Coma science, respectively, and present the Comet Interceptor mission to address them by making unique multi-point measurements at a much more pristine type of comet. The exact sources and mechanisms driving activity in comets remain a puzzle after Rosetta. Due to the flyby nature of the mission, Comet Interceptor will not be able to monitor changes in activity. However, detailed observations of the surface and the coma will be combined to address this point (i.e., potentially linking the coma structures to nucleus surface features). These are described within Sect. 2.3, in the Coma theme, below.

### Theme 1: Comet Nucleus Science

#### Introduction

This science theme focuses on the nucleus, looking to answer the following questions. What are the:

- surface composition,
- shape,
- morphology, and
- structure

of the target object?

These questions can only be answered by *in situ* (as opposed to remote sensing from the Earth) measurements, in particular given the relative size of the nucleus with respect to the coma which hides the nucleus for observations from Earth and near-Earth based observatories.

Characterisation of the nucleus by Comet Interceptor will provide information on its bulk properties (shape, rotation rate, surface structure, etc.), which will in turn provide constraints for surface features’ formation timescales. It is unknown whether there might be craters, depressions, layers, regolith, or boulders present – these could provide unique insights into early surface evolution. Do primordial small bodies display a singular primordial surface type, or do they show surface diversity at different size scales? Any impact crater population might provide an unmodified record of early bombardment in the Solar System, as few impacts are expected in the OC. This will give a unique insight into the early Solar System’s accretion processes and the characteristics of primordial small planetesimals.

All comets already visited by spacecraft have undergone shape changes through sublimation and specific surface evolution processes during repeated perihelion passages. Recent results suggest that bi-lobate nuclei are common (e.g., Giotto, Rosetta, and other Jupiter Family Comet (JFC) missions). A comparison between a Long-Period Comet, LPC, and New Horizons studies of 486958 Arrokoth will be particularly instructive, as both these volatile-rich bodies are largely unaltered since formation, but have likely formed and subsequently resided in different Solar System contexts. An LPC (and even more-so a Dynamically-New Comet, DNC) would have experienced few changes due to insolation in recent times, when compared to JFCs, but could have had significant processing earlier in its lifetime. Characterisation of the difference and similarities will be invaluable, examining surface morphology and comparing to comets imaged by previous missions. In addition to this, composition measurements of a primordial surface, in comparison with Rosetta results, may tell us about chemical processing as comets evolve.

#### The Origin of Different Classes of Comet Nuclei

**Fig. 2:**
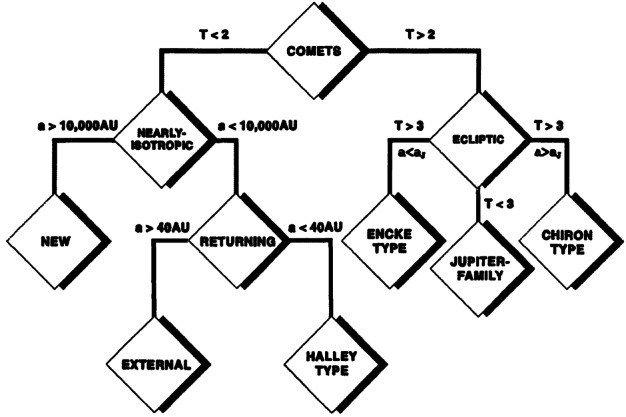
Classifications of comets by Levison (1996). T refers to the Tisserand parameter, whilst parameter $a$ refers to orbital semi-major axis

Comets of all kinds formed in the outer part of the Solar System’s protoplanetary disc, where ices could condense, and the giant planets also formed. As these planets settled into their final orbits, the small bodies in this region were scattered to form the various comet reservoirs that we know today: the Scattered Disc of the Kuiper Belt (KB), the source of low-inclination JFCs; the OC at the edge of the Solar System, which supplies the population of LPCs; and the recently identified probable reservoir of icy bodies in the main asteroid belt that occasionally show activity as Main Belt Comets. The taxonomy of Levison (1996) splits comets into low-inclination ‘Ecliptic’ comets from the KB, of which JFCs are the dominant subset, and ‘Nearly Isotropic Comets’ (NICs), which can have any inclination, including highly retrograde orbits, from the OC (Fig. 2). NICs are subdivided into the LPCs (orbital periods >200 years) and the Halley Type Comets (HTCs) with shorter periods. There is some debate on the origin of HTCs, with competing models suggesting that these can also come from the Scattered Disc (e.g., Levison et al. 2006; Wang and Brasser 2014; Fernández et al. 2016, and Nesvorný et al. 2017). LPCs can be further divided into DNCs and returning comets, depending on whether or not a previous perihelion distance that an object had was thought to be within the planetary region.

Planetary systems naturally scatter and eject most of the planetesimals that they form from their primordial discs of dust and gas. These planetesimals travel the Galaxy as interstellar objects (ISOs).

It was thought that the OC was primarily populated by bodies that formed near to Jupiter and Saturn, while the objects that would populate the KB formed more or less *in situ* at their current distances from the Sun (e.g., Dones et al. 2004; Duncan and Levison 1997), but modern thinking suggests a broader mixing of comets from different original locations in the protoplanetary disc being scattered to the various reservoirs (e.g., Dones et al. 2015). Results from the Stardust mission showed that material that must have formed in the warmer regions in the inner Solar System was incorporated into comets, suggesting widespread mixing within the disc (Brownlee 2014). It is therefore likely that all Solar System comets have broadly similar initial properties (although perhaps differing in composition, especially at an isotopic level, depending on where the majority of their component ices condensed from gas; see Sect. 2.2.4 and Rubin et al. 2020 for details). What we can expect to be different between JFCs (and perhaps HTCs) and LPCs is the degree to which they have subsequently evolved: the more distant OC stored comets in a colder environment (∼10 K vs. ∼40 K in the KB; Weissman et al. 2020), and LPCs (and especially DNCs) enter the inner Solar System directly from this cold reservoir, while JFCs have evolved through a period (${\sim} 10^{4}$ years) in the Centaur region (Volk and Malhotra 2008), with orbits between the giant planets, where significant activity can be expected to modify at least the surface layers. As such, even a ‘new’ JFC can be expected to be significantly modified from its primitive state, while LPCs should retain largely similar properties from the time that they were first ejected into the OC (discussed in more detail in Sect. 2.2.5).

The motivation for a mission to a less evolved comet is clear: new comets retain, to some degree, the properties of the building blocks of planets, and are at least highly primitive bodies, essentially unaltered since their ejection into their respective reservoirs, expect for cosmic ray processing, e.g. Garrod (2019). How much processing comets underwent during their formation in the disc, prior to ejection, is the subject of considerable debate (see, e.g., Weissman et al. 2020) and another reason to visit a comet from the OC, which retains the surface properties from the last interactions it had before ejection. There are two main theories of comet formation still under consideration following Rosetta, both of which are supported by evidence from the mission, but neither is entirely satisfactory. These are hierarchical accretion (e.g., Davidsson et al. 2016) and accretion of ‘pebbles’ by streaming instabilities (e.g., Blum et al. 2017). Both make predictions for the size scale of typical constituent building blocks of comets, and both sets of scale in features can be found in Rosetta data. Whether or not these features are primordial or evolutionary is the key question to advance this debate. There is also considerable interest in the question of how many collisions cometary nuclei have undergone, and the effects that these collisions would have on the properties of their ices, focussing on the period before the comets were ejected from the disc to their reservoirs. While the OC is so vast that collisions between bodies after ejection are virtually impossible, Jutzi et al. (2017) and Schwartz et al. (2018) both show that collisions large enough to alter the bulk shape of nuclei (perhaps forming the typical bilobed structure seen in many comets) were almost impossible to avoid in the disc prior to ejection. Investigating a comet that has not been significantly further altered since its last pre-ejection collision would be of great value to better constrain these models, and reveal how much these collisions locally alter material, compared with the (supposed) unaltered nucleus ices further from the collision site(s).

#### Bulk Properties

**Fig. 3:**
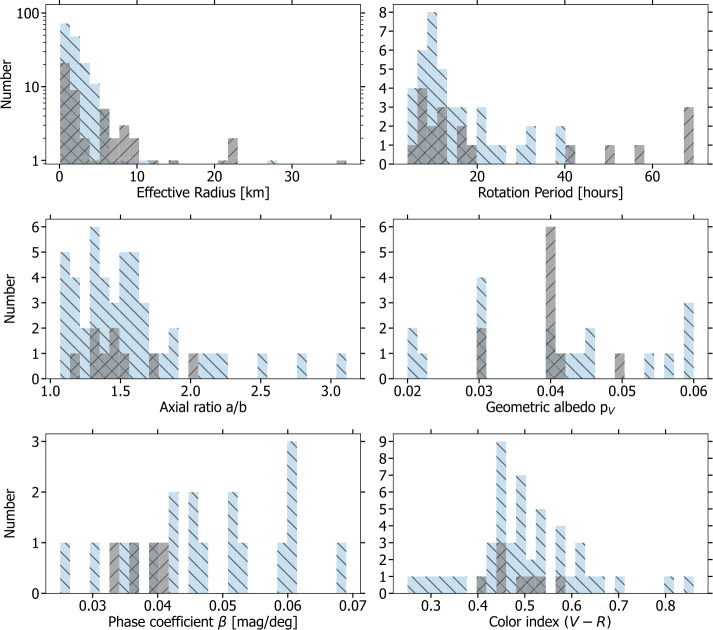
Histograms showing the range of observed comet properties (JFCs in blue and Halley-type/LPCs in grey hashing). The figure provides an overview of the range of measured values and the sample size of each parameter. In case a comet has multiple measurements of the same property, the most recently reported sufficiently precise measurement is displayed. The effective radius histogram is limited to thermal-IR measurements from Fernández et al. (2013) and Bauer et al. (2017). The axis ratios plotted are lower limits for all comets except for those visited by spacecraft. Adapted from Knight et al. (2023)

The current understanding of the bulk properties of comet nuclei is a result of both remote telescope observations and spacecraft visits. Spacecraft *in situ* observations provide precise measurements of the physical properties of individual nuclei. On the other hand, telescopes both in the optical and in the thermal infrared allow the coarse characterization of numerous nuclei, therefore enabling population studies. One of the easiest to constrain nucleus properties is size. Since the effective radius of comet nuclei (the radius of a sphere having the same volume as the comet nucleus) can in principle be derived from single-epoch observations, the sizes of over 200 comets have been determined (Knight et al. 2023). This large database reveals a broad diversity of comet sizes: from hundreds of metres to a few tens of km (see Fig. 3) and has been used to derive the size-frequency distribution (SFD) of comets. The SFD of comet nuclei is believed to bear evidence of the processes involved in their formation and subsequent collisional and/or activity-driven evolution. De-biased Comet SFD studies show interesting trends indicating that the average size of LPCs is larger than that of JFCs (Bauer et al. 2017). Yet important questions such as whether there is a paucity of objects smaller than 2 km (see Bauer et al. 2017) and whether comets are a collisionally evolved population remain unresolved (see Weissman et al. 2020).

Another parameter broadly used to analyse the formation and evolution of comet nuclei is shape. The shapes of six nuclei have been studied in detail from *in situ* observations. The observations from the Rosetta mission to 67P stand out with their unprecedented resolution, which enabled a global 3D shape model with resolution down to metre scale (Preusker et al. 2017). In at least eight exceptional cases in which the comets approached the Earth sufficiently and could thus be observed by radar, shape models could be constructed (see Knight et al. 2023). For other comets, however, nucleus shapes can only be studied in terms of elongation. Measuring the peak-to-peak amplitude of the rotational lightcurve, $\varDelta $m, provides a lower limit of the axial ratio of a/b of the comet nucleus. In principle it is possible to derive convex shape models of comet nuclei, provided that the comets are observed at a wide variety of different observing geometries, but this technique has so far only been applied to 67P in preparation for Rosetta (Lowry et al. 2012). Compiling elongation estimates from the different methods, Kokotanekova et al. (2017) determined a median axial ratio of a/b = 1.5 for JFCs, similar to the previous estimates from Lamy et al. (2004) and Snodgrass et al. (2011). As it can be seen in Fig. 3, the measured minimum a/b spans a range of ∼1.0 to >3.0. The largest known axial ratio belongs to 103P (Thomas et al. 2013) which is one of the four comet nuclei visited by spacecraft that has a bilobate shape (the others are 1P Keller et al. 1986, 19P Britt et al. 2004; Oberst et al. 2004 and 67P Sierks et al. 2015). Moreover, the radar observations of 8P are also best modelled by assuming a contact binary shape (Harmon et al. 2010). This noticeable overabundance of highly elongated/bilobate objects in comparison to other small-body populations suggests some important difference in formation and/or evolutionary processes, which has not yet been fully explained. Whether or not the first LPC nucleus to be imaged *in situ* also shows a contact binary morphology will provide an important constraint on this question, and point to whether it is more likely attributable to a formation or an evolutionary process.

Rosetta’s observations were also the first to enable the characterization of 67P’s opposition effect; the non-linear increase of its phase function close to $\alpha = 0^{\circ}$ (Fornasier et al. 2015; Masoumzadeh et al. 2017; Hasselmann et al. 2017). Few of the other comets visited by spacecraft, or observed remotely from the ground, have been observed close to opposition and all other comet phase curves are well described by linear phase functions. It is possible that the phase function slope at moderate phase angles ($\alpha $ ${\sim }5\text{--}60^{\circ}$) can be used to reveal the level of erosion of the comet surface (see Longobardo et al. 2017; Kokotanekova et al. 2018; Vincent 2019). It remains to be seen if this also applies to LPCs, with very different surface evolutions, as all phase function data so far is on JFCs.

It is important to note that the average colours of JFCs, LPCs and dormant comets are indistinguishable within the uncertainties for each class (Jewitt 2015). Finally, Rosetta has revealed that the surface spectrum of 67P changes as the comet passes through perihelion due to the comet’s seasonal water-ice cycle: the nucleus’ mean spectral slope changed by ∼30% or 50% (Fornasier et al. 2016; Filacchione et al. 2020). Polarimetric studies of comet surfaces have not yet been performed during a spacecraft visit. There are only two JFCs for which polarisation studies from the ground have been published, 2P/Encke (Boehnhardt et al. 2008) and 209P/LINEAR (Kuroda et al. 2015). These data are too limited to extract any general conclusions about the bulk polarisation properties of comet nuclei besides pointing out the similarities of these two comets and known dark asteroids (Kiselev et al. 2015).

Thermal properties dictate the temperature distribution throughout the nucleus, and are thus key to describe physical and chemical processes occurring in response to solar illumination. The thermal inertia of a comet nucleus, for instance, drives its ability to adapt its temperature to a change in local insolation. A material with a large thermal inertia takes longer to adapt its temperature to changing illumination conditions compared to a material with low thermal inertia. Low thermal inertia – and therefore low thermal conductivity – means that the interior of nuclei remains cold when the surface is heated by the Sun, and can therefore retain more volatile ices (i.e., can be more ‘pristine’). Estimates for comets made from spatially unresolved observations of the nucleus allowed a value lower than $50\,\text{ J}\,\text{K}^{-1}\,\text{m}^{-2}\,\text{s}^{-1/2}$ to be derived. Owing to spacecraft flybys in the 2000s, thermal inertia has since been derived from radiance measurements, from which temperature can be inferred, on the surface with spatially resolved maps of comets 9P, 103P and 67P. They point toward a low thermal inertia, between 50 and $200\text{ J}\,\text{K}^{-1}\,\text{m}^{-2}\,\text{s}^{-1/2}$ for 9P (Davidsson et al. 2013), and less than $250\text{ J}\,\text{K}^{-1}\,\text{m}^{-2}\,\text{s}^{-1/2}$ for 103P (Groussin et al. 2013). The suite of instruments on-board Rosetta indicates that 67P’s thermal inertia is between 5 and $350\text{ J}\,\text{K}^{-1}\,\text{m}^{-2}\,\text{s}^{-1/2}$ (Leyrat and Blain 2015). It varies across the surface (Leyrat and Blain 2015), perhaps due to variations in material properties such as density or porosity, between consolidated and unconsolidated terrains. Additional estimates in the near-subsurface were possible, down to 1 and 4 cm below the surface: they point to a thermal inertia of the order of 10 to $60\text{ J}\,\text{K}^{-1}\,\text{m}^{-2}\,\text{s}^{-1/2}$, and lower than $80\text{ J}\,\text{K}^{-1}\,\text{m}^{-2}\,\text{s}^{-1/2}$ (Gulkis et al. 2015; Choukroun et al. 2015; Schloerb et al. 2015; Marshall et al. 2018). Finally, the MUPUS measurement at the landing site of Philae suggests a local thermal inertia of the order of $120\text{ J}\,\text{K}^{-1}\,\text{m}^{-2}\,\text{s}^{-1/2}$ (Groussin et al. 2019). These thermal inertia estimates depend strongly on the model used to derive them: they should be taken only as an indication that cometary material close to the surface has a low thermal inertia.

Indeed, temperatures are not directly measured with remote sensing instruments, but rather the infrared or sub-mm flux is detected, or the brightness temperature is measured. The kinetic temperature, which gives information on thermal properties of a surface, must be retrieved through models. Measurements in the near infrared can be contaminated by reflected solar radiation (Keihm et al. 2012). Instruments detect a nonlinear average of potentially very different temperatures in the field of view, with large and small-scale topographic features and perhaps compositional heterogeneities. On Rosetta, MIRO measurements were affected by both the thermal and the optical properties of the material, which made the interpretation challenging. A yet larger caveat comes from the lack of a thermal infrared instrument on the Rosetta payload. Lower limits for the temperature derived from VIRTIS-M were effectively restricted to the dayside of the nucleus (Tosi et al. 2019). Kinetic temperatures over complete diurnal cycles and for the same layer could not be retrieved, and thermal inertia maps were derived with large error bars (Groussin et al. 2019). Resolved images of both the day and night sides in the thermal infrared are required to put stronger constraints on thermal models of cometary nuclei.

#### Morphology of Nuclei

**Fig. 4:**
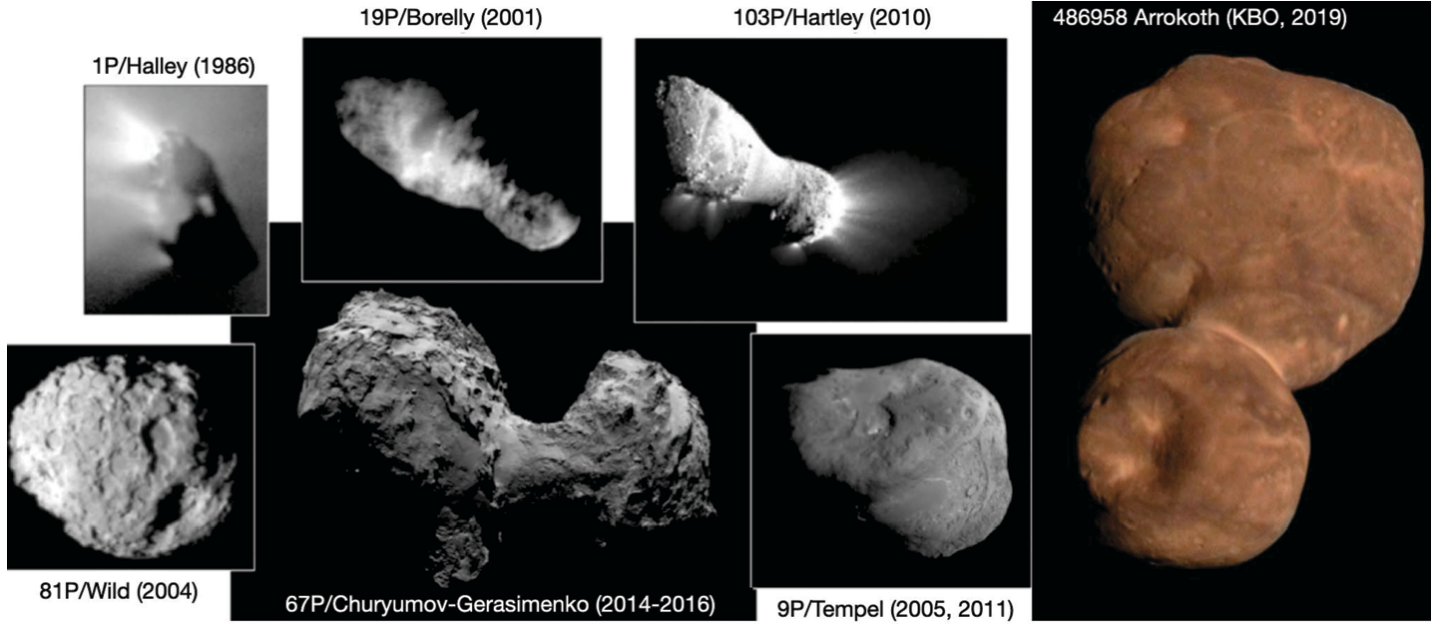
A subset of the cometary nuclei that have been visited by spacecraft and on the right an image of Arrokoth, a Kuiper Belt Object, which is a member of the Cold Classical population which is not believed to be a significant source of JFCs. Objects are not shown to scale

As cometary surfaces are almost impossible to resolve in Earth-based observations, our information about surface morphology of comets is based on flyby or orbital missions (with Rosetta currently the only mission to orbit around a comet for a prolonged amount of time). Figure 4 shows the shape and general morphology of all of the comets that have been imaged *in situ*. It is useful to draw comparisons between the morphologies observed on comets (mostly JFCs) and Arrokoth, the cold classical KBO visited by NASA’s New Horizons Mission in 2019. Whereas Arrokoth signifies a nearly primordial body characterised by low colour diversity, uniform textures, lack of topographical complexity, and a general lack of putative impact craters, circular features overall, or evidence of tectonics (McKinnon et al. 2020), comets that have spent a significant amount of their life in the inner Solar System display clear evidence of surface evolution and overall weathering as is demonstrated by the higher degrees of surface roughness, topographical complexity, morphological diversity of terrains, and presence of smooth regions suggestive of weathered and eroded fine-scale materials (e.g., Keller et al. 1986; Britt et al. 2004; A’Hearn et al. 2005, 2011; Veverka et al. 2013; Sierks et al. 2015; Thomas et al. 2015).

**Fig. 5:**
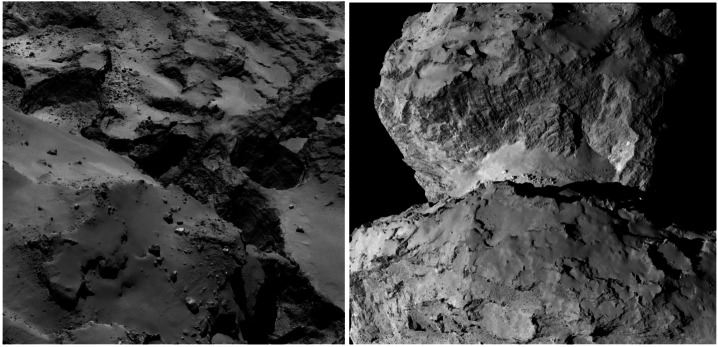
Pits on the surface of comet 67P/Churyumov-Gerasimenko (left). Hathor cliff above the ‘neck’ region between the two lobes of 67P (right). ESA/Rosetta/MPS for OSIRIS Team MPS/UPD/LAM/IAA/SSO/INTA/UPM

Rosetta demonstrated how activity caused by seasonal sublimation of volatiles (mostly water-ice but also other types of ice), as comets cross the snow-line during the perihelion part of their orbit, can account for various fine-scale morphology on cometary surfaces, with clear evidence of seasonal evolution, e.g., El-Maarry et al. (2017, 2019). However, it is unlikely that major landscape evolution occurs that way, at least on seasonal scales (El-Maarry et al. 2017): seasonal erosion causes changes on scales of order 1–10 m, while there are pits (Fig. 5, left) approximately 100 m in diameter, and the cliff (Fig. 5, right) above the ‘neck’ region between the two lobes of 67P is around a kilometre high.

Therefore, we can treat or consider JFCs, and KBOs such as Arrokoth, as two opposite end-member points in a spectrum of bodies in varying stages of evolution. Visiting a more pristine LPC would add a pivotal midpoint across that evolutionary path that can further explain how primordial KBOs transition to heavily evolved comets, and at which stage major landscape processes occur. JFCs go through a transitional “Centaur” phase as their orbits dynamically evolve to shorter orbits that place them in the inner Solar System. It is possible that comets undergo substantial changes during that time as hypervolatiles can sublimate beyond the water ice snowline. However, the degree of that change is currently unknown. A major question that can be answered by Comet Interceptor is where would LPCs, or generally comets that have not gone close to the Sun many times in their lifetime, place in that evolutionary path? Would they be closer in morphology to KBOs, or would they have developed some morphological and textural variety already during their early formation stages (see Sect. 2.2.6 below)? Do they show evidence for collisions before their ejection to the OC, and if so, what record do they retain of the primordial impactor population (e.g., crater size distribution)? Therefore, the possibility to encounter and investigate either an LPC, or especially a DNC, would offer more insights to the evolutionary path of comets and the conditions in the early Solar System more generally.

#### Composition of Nuclei

Most of our knowledge on the composition of comets comes from measurements (either *in situ* by spacecraft, or remotely via spectroscopy) of the gas coma, which is discussed in detail in the coma science section below (see Sect. 2.3). Broadly, we understand the composition of nucleus ices indirectly, by working backwards from sublimated gasses in the coma, or from the products of further gas phase photochemistry. However, direct measurement of the nucleus composition is of great importance to comet science:

- to discover the starting point for these sublimation/ chemistry models,
- to understand how (in)homogeneous the nucleus is,
- and to assess the composition of the original building blocks of comets (and planets) independent of our (lack of) understanding of evolution and activity processes.

**Fig. 6:**
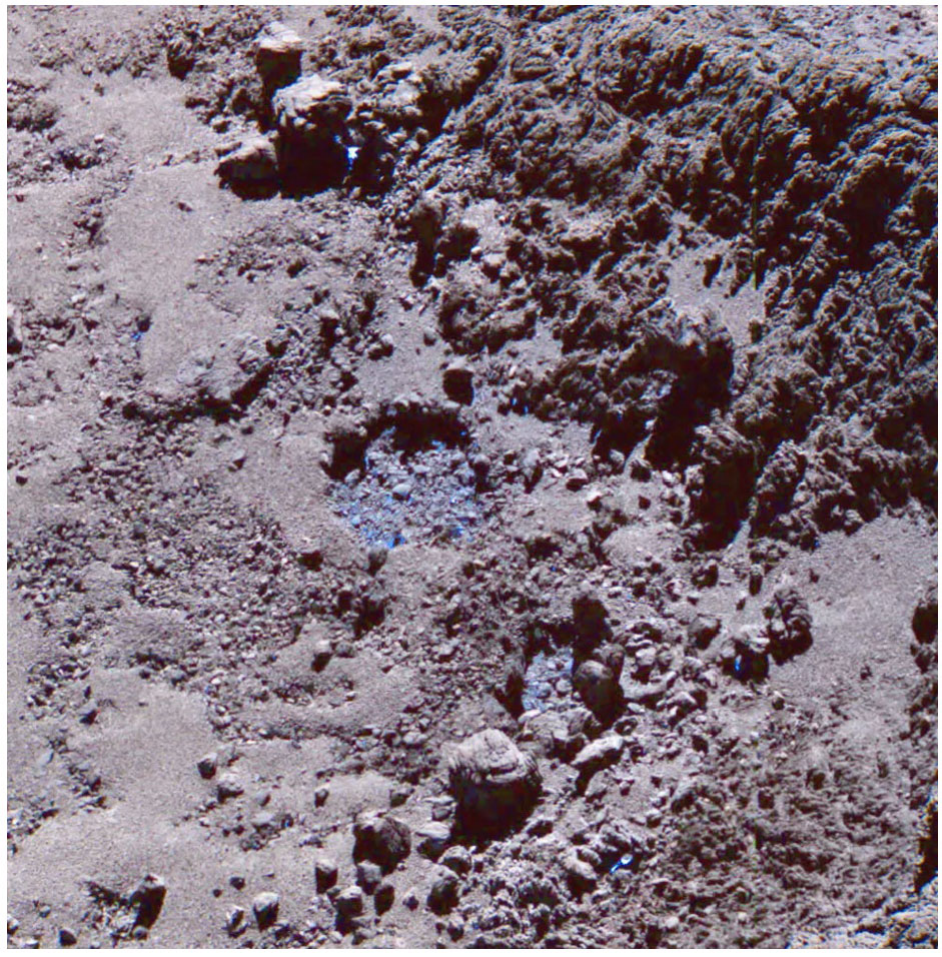
A water ice-filled depression on the surface of 67P’s nucleus. This image is a false-color composite, where the pale blue patches highlight the presence and location of water-ice. ESA/Rosetta/MPS for OSIRIS Team MPS/UPD/LAM/IAA/SSO/INTA/UPM/DASP/IDA

We are limited to measurements of the surface composition, as the only attempt to directly measure the interior composition of a comet nucleus, the SD2 drill on the Philae lander, failed to collect a sample (Boehnhardt et al. 2017). Mass spectroscopy results for surface dust returned by Philae were inconclusive, but did show a high proportion of organic compounds (Boehnhardt et al. 2017), consistent with remote sensing measurements. As discussed in Sect. 2.2.3 above, spectroscopy, both unresolved from telescopic observations and resolved from spacecraft encounters, reveals comet nuclei to be largely featureless in the visible and near infrared, with reddish slopes (but shallower than many KBOs) and very low albedo (e.g., Quirico et al. 2016). A broad 3.2 μm absorption was identified by the Rosetta VIRTIS instrument at 67P, which has been interpreted in a variety of ways, including salts, organic compounds, and/or silicates (Poch et al. 2020; Raponi et al. 2020; Mennella et al. 2020). Features due to water ice are, perhaps surprisingly, limited in their presence, but detected at comet 9P (Sunshine et al. 2006). They are only seen in Rosetta data in localised spots (Barucci et al. 2016; Fornasier et al. 2023), where fresh subsurface layers (e.g., Fig. 6) have been uncovered (e.g., by cliff collapse; Filacchione et al. 2016a), or as short-lived frosts deposited during the comet night time (De Sanctis et al. 2015)). A region of CO_2_ ice was also identified at 67P (Filacchione et al. 2016b). Nuclei are mostly too faint to be detected at shorter and longer wavelengths from Earth-based observations, but UV spectroscopy from the Alice instrument on-board Rosetta revealed a featureless blue slope (Feaga et al. 2015). At sub-mm wavelengths, Rosetta/MIRO could only constrain nucleus thermal emission, not give compositional information. Unresolved mid-infrared spectroscopy with the Spitzer space telescope shows broad features attributed to silicates, with similarities to D-type asteroids and Jupiter Trojans (Kelley et al. 2017). Spacecraft observations, made *in situ* at these wavelengths, have yet to be attempted, but are a promising direction to take. These wavelengths contain a wide array of features seen in common minerals and organic ices, and could reveal both composition and its variation across the nucleus surface, in resolved spectroscopy and/or imaging.

#### Evolution of Cometary Nuclei

Both the mechanical and thermal processing of comet nuclei (Weissman et al. 2020) occur during distinct phases of a nucleus’s life, e.g.:

- during formation and early evolution in the protoplanetary disc.
- during the long period of storage in the comet reservoirs.
- and as an active comet upon return to the inner Solar System.

**Fig. 7:**
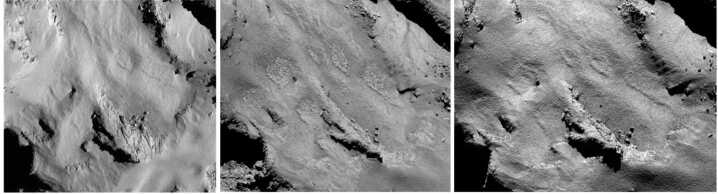
Surface morphology changes due to fall-back of dust on the surface of 67P/Churyumov-Gerasimenko. ESA/Rosetta/MPS for OSIRIS Team MPS/UPD/LAM/IAA/SSO/INTA/UPM

The active comet phase is the one that is best understood, following the results from Rosetta, as the evolution of the nucleus is driven entirely by the comet’s activity and therefore by the energy it receives from the Sun. Erosion of the surface has long been understood as a consequence of sublimation of nucleus ices, with metres of the surface lost, on average, per perihelion passage (e.g., Whipple 1950; Britt et al. 2004; Veverka et al. 2013; Keller et al. 2015). However, a surprising result from the Rosetta mission was the importance of fall-back of material lifted into the inner coma, with some areas of the nucleus blanketed by deep layers of fine material (e.g., Thomas et al. 2015; Marschall et al. 2020; Cambianica et al. 2021). In models of the nucleus consisting of pebbles, fall-back of decimetre-sized chunks leads to both surface morphology changes (Fig. 7) and evolution of near-surface composition, as subsequent activity from the fall-back material is driven by water ice retained within these ‘chunks’, which have a lower abundance of the more volatile ices whose activity lifted the chunks from other areas in the first place (Fulle et al. 2020a). The many cycles of activity seen by short period comets mean that their surfaces have undergone significant evolution, while an LPC encountered at 1 au will have relatively little recent evolution, depending on where exactly its activity began on the inbound leg (models range from 35 to 85 au; Jewitt et al. 2021; Fulle et al. 2020b), and on model-dependent levels of erosion and fall-back during this time.

The earliest phase in comet evolution, which the nuclei spent in their formation zone at 5–30 au, could last anywhere from 2.5 to several hundred million years (various models have different timing for the instabilities in the giant planet orbits that scattered comets into their reservoirs, e.g., Nesvorný et al. 2018; Morbidelli et al. 2018; Pirani et al. 2019, 2021). The OC could not have been populated while the Sun was still in its embedded star cluster phase – whose duration was perhaps a few Myr (e.g., Adams 2010; Pfalzner and Vincke 2020; Parker 2020) – because the Sun’s tidal radius was too small (Tremaine and Dones 1993; Wyatt et al. 2017). When it comes to the survival of volatile species, this phase is significant: Davidsson (2021) reports that nuclei in the protoplanetary disc with diameters ranging from 4 to 200 km can lose all their condensed CO ice on short timescales (smaller than the minimum time to eject them in the reservoirs), through a combination of protosolar and long–lived radionuclide heating. This may have been avoided if cometesimals formed late enough (Davidsson et al. 2016), or in a region of the disc with relatively low abundance of radionuclides, which has been suggested by some observations (e.g., analysis of four Stardust samples indicate that 81P never contained any appreciable ^26^Al; Levasseur-Regourd et al. 2018). The drivers of evolution in this phase can also be expected to include collisions (Jutzi et al. 2017; Schwartz et al. 2018), although models of the timescales for CO-driven cometary activity suggest that erosion of ice-rich cometesimals in this early environment could dominate evolution over collisions (Fulle et al. 2020b).

Subsequently, and because the time of residence in the OC or the KB is very long, a significant fraction of the bulk of most comet nuclei can be affected by superficial heat sources, even if the thermal diffusivity is extremely low. Davidsson (2021) reported that, for objects large enough to have any pure condensed CO ice remaining in their bulk when they are ejected in their reservoirs, the long-term survival of supervolatile ices largely depends on whether nuclei are scattered in the OC, or in the KB. Indeed, objects reaching the OC could get subsurface temperatures low enough for CO gas, if diffusing from the deep interior, where it was heated due to radiogenic decay, to condense near the surface. In the KB, the equilibrium temperature ranges from 30 to 50 K, so that, for objects typically smaller than 4 km, all hypervolatiles initially present as pure ices should sublimate during the time of residence in this reservoir, even without any radiogenic heating (De Sanctis et al. 2001; Choi et al. 2002; Jewitt 2004; Davidsson 2021).

We emphasize again that, for OC comets, the thermal processing prior to the injection in this reservoir does not guarantee that a nucleus stored there can preserve a pristine inventory of hypervolatiles. In terms of internal structure expected for OC comets, Davidsson (2021) suggests that any nuclei exposed to the proto-Sun’s intense heating would lose not only hypervolatiles condensed as pure ices, but also CO_2_, down to a depth of ∼30 m. In addition, partial crystallization could occur in the upper ∼200 m. As a consequence, the near-surface layers of a comet nucleus may be significantly processed: even DNCs cannot be completely pristine, and some might have lost a significant amount of hypervolatiles prior to their scattering into the OC. In addition, Stern and Shull (1988) suggest that up to 20% of comet nuclei stored in the OC could have been heated to at least 30 K down to several dozen metres below the surface, due to the passage of luminous stars during the history of the Solar System. Most of them may have been heated to 45 K in the uppermost 1 m-layer due to stochastic supernovae events. This would lead to the formation of a surface layer depleted in hypervolatiles. Stern (2003) further reports that passing stars and supernovae heating events could modify the primordial composition of comet nuclei down to 5 to 50 m (for heating due to passing stars), and to 0.1 to 2 m (for heating due to supernovae events).

However, an extremely significant difference between JFCs and DNCs coming from the OC lies in their subsequent orbital evolution, which brings them to the orbits on which we observe them. Indeed, an intermediate evolution phase exists almost exclusively for Centaurs, of which a fraction become JFCs, during which comet nuclei are perturbed into their final orbit through a chaotic orbital evolution in the giant planet region. Processing during this phase intensifies, due to increasing equilibrium temperatures, and close passages to massive planets. Because the time spent in the giant planet region is significant (typically 10 Myr, Levison and Duncan 1997; Tiscareno and Malhotra 2003) the resulting processing is also substantial. This phase is non-existent for nuclei coming from the OC, which come from this reservoir to the inner Solar System in a more rapid and direct pathway.

Huebner et al. (2006) discussed the outcomes of such different injection types, which may result in extensive changes of the internal composition and structure of comets. Similarities between the structures resulting from the two types of orbital evolution are:

1. Surface temperatures, driven by the surface heat balance, and
2. Water and CO_2_ gas production, which are controlled by erosion, keeping both water and CO_2_ ice close to the surface.

However, the significant differences resulting from the two orbital evolutions are mostly related to the location of the CO sublimation front (and hypervolatiles in general), and the amorphous/crystalline ice interface. The CO sublimation front for JFCs should be located hundreds of metres below the surface, while it remains very close to the surface for DNCs. In terms of activity, this is reflected in the fact that CO production is typically continuous for JFCs, or at least with a pattern which does not follow the water emission, and the production rate for the JFC nucleus is significantly smaller (ten times lower in their example) than for a DNC. Water and CO_2_ sublimation remain, however, characterized by peak emissions towards the perihelion of each orbit. The simulations reported in Huebner et al. (2006) also showed the formation of a transient dust mantle, which was destroyed when the last orbital change occurred.

In conclusion, any comet nucleus coming from the OC would be significantly less altered and more informative of the processes that shaped the Solar System in its early phases, when compared to the JFCs explored in space thus far.

### Theme 2: Comet Environment Science

#### Introduction

This theme focuses on the coma, looking to answer the following questions:

- What is the composition of the gas and dust in the coma?
- How does it connect to the nucleus (i.e., how does cometary activity work)?
- What is the nature of its interaction with the solar wind?

These topics are addressed through observations of the gas, dust and plasma that are described in the subsections below. They are linked to each other, and also to the studies of the nucleus described in the previous section, through the processes of cometary activity; ices sublimating to gas, and lifting dust, followed by the decoupling of these and the photochemistry that generates the products we observe on large-scales. Data from telescopic observations and previous space missions have given us clues to understand all of these steps, but many puzzles remain. Measuring the composition and distribution of volatile species in the coma will help in understanding the activity processes of an LPC compared with the more evolved objects visited to date:

- What are the relative abundances of molecules of high volatility such as CH_4_, CO and CO_2_ with respect to water in an LPC versus JFCs (67P) and HTCs (1P) at similar heliocentric distances?
- Is there evidence for or against hyperactivity, i.e., significant activity being driven by sublimation from icy ‘chunks’ in the coma, as seen at 103P by EPOXI?
- Are there differences in isotopic composition, e.g., in D/H, and other species if sufficiently abundant?

For each of these questions, Comet Interceptor will test whether or not the phenomenon is evolution-related, and will inform the interpretation of ground-based observations of other comets. Comet Interceptor will also perform unique observations of the coma from three different positions simultaneously, due to its multispacecraft configuration. This will allow gas, dust, and plasma distributions or boundaries to be described in a 3-D way. This was not possible with previous missions, which sampled only a single location at a time, and will allow separation of spatial and temporal variations. The multipoint *in situ* plasma measurements will be complemented by Energetic Neutral Atom (ENA) observations, which may provide a more continuous observation of the variability of the solar wind, giving a clearer picture of which plasma variations are due to external influences.

#### Bulk and Isotopic Composition, and Distribution of Gas and Dust

The bulk of our knowledge of the composition of comets comes from studying the gas coma. Remote observations give a comparatively less detailed picture but of a larger number of comets, while *in situ* measurements with mass spectrometers, in particular from Rosetta, have revealed a wealth of detail of just a few. Unfortunately, there have not been any good opportunities yet to link these approaches. Although a campaign of remote observations did support Rosetta (Snodgrass et al. 2017), 67P was not particularly bright around its 2015 perihelion and could not be studied with high resolution spectroscopy, for example. The comet also presented a poor observation geometry, being located on the other side of the Sun with respect to Earth. Much of our knowledge of comet composition (especially on an isotopic level) from telescopes is based on LPCs, which are often more active and brighter. A mission to a bright LPC would present an opportunity to compare high resolution spectroscopy at a range of wavelengths with *in situ* mass spectrometry measurements, calibrating our understanding of the much wider observed population. Modern infrared and sub-mm facilities, such as the ESO Very Large Telescope and the other 8–10 m class telescopes, and the Atacama Large Millimetre/submillimetre Array, which did not exist at the time of Giotto, are suitable for observing molecular species thought to be directly released from the nucleus.

It is well established that within about 3AU from the Sun the main driver of cometary activity is the sublimation of water ice. At larger distances, more volatile species like CO or CO_2_ are likely to play a major role (e.g., Meech et al. 2017). There remains a lot of uncertainty about where and how the transition between driving species takes place, and whether or not there are differences in this between new and returning comets. It has long been observed that DNCs tend to be brighter (more active) than periodic comets (the ‘fading problem’: Oort 1950; Dones et al. 2004); the most significant difference between DNCs and returning comets appears to be greater activity at larger distances in the former population, which implies different driving species (Meech and Svoren 2004). Comet Interceptor will measure the absolute and relative densities of the main neutral volatiles (H_2_O, CO, and CO_2_) along the flyby trajectory, allowing us to derive production rates from these measurements, and show which is dominant in an LPC at ∼1 au.

**Fig. 8:**
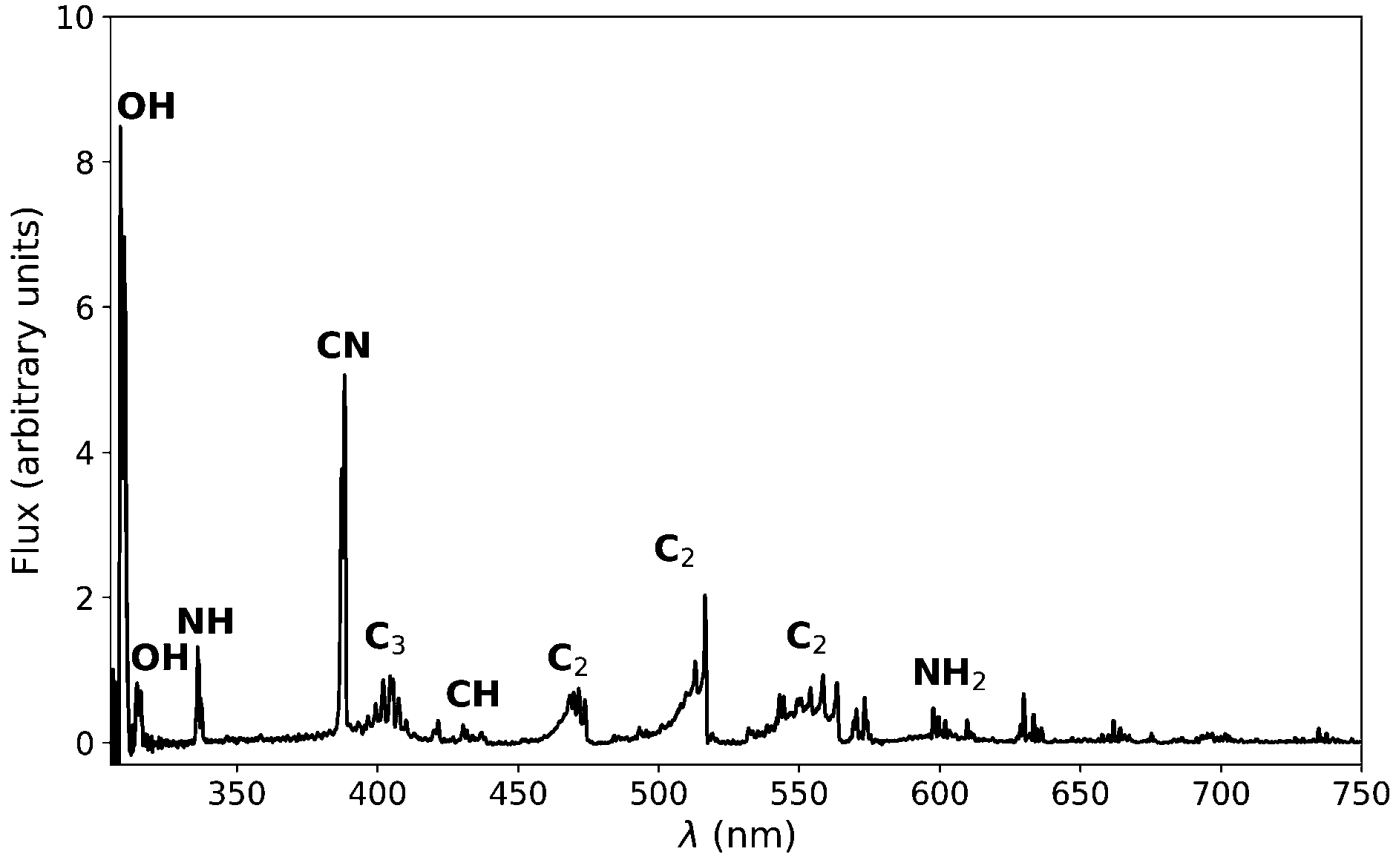
Typical comet spectrum from ground-based visible observations, with key emission features marked. Major components such as water or CO_2_ are not observable and require space missions to be characterised. (Image courtesy of C. Opitom)

Although ground-based composition observations do not rival the comprehensive information that can be provided by *in situ* spacecraft measurements, remote observations still provide extremely valuable data on bulk composition (e.g., Fig. 8). Differences of composition between JFCs and more pristine comets coming from the OC are starting to emerge from ground-based observations, with the most highly volatile species (such as CO, CH_4_, C_2_H_6_, and C_2_H_2_) being depleted in JFCs compared to OCCs (Dello Russo et al. 2016). However, this has not been confirmed *in situ*. Ground-based observations of highly volatile molecules in infrared/radio domains are rarely simultaneous: for example, CO in the M-band and other organics in the L-band are not observed simultaneously, although most species are usually observed with H_2_O, or its proxy OH, to derive mixing ratios with respect to H_2_O. This makes the measurement and comparison of relative abundances difficult in some cases, especially for data taken by old-fashioned infrared spectrometers. A large dispersion of mixing ratios observed in ground-based observations might be partially explained by such non-simultaneous observations. Comet Interceptor will provide an unprecedented opportunity to measure gas composition *in situ* and compare the abundance of molecules of high volatility such as CO, CH_4_, C_2_H_6_, and CO_2_ with respect to water in an LPC versus JFCs (67P) and evolved HTCs (1P).

Aside from the volatiles mentioned above, Comet Interceptor will make detailed compositional measurements that are only possible *in situ*. The Rosetta spacecraft revealed the presence of complex and diverse organic molecules in the coma of 67P, including key species for prebiotic chemistry, some being observed in the coma of a comet for the first time (see Altwegg et al. 2017a). These organics cannot be detected through ground-based observations with current technology, preventing us from assessing the variation of their abundances between comets and, in particular, between primitive LPCs and processed JFCs. Obtaining an inventory of (complex) organic molecules and other species important in prebiotic chemistry in a primitive LPC will delve further into the role of comets in transporting organic matter to the early Earth (Marty et al. 2016). A surprising result of Rosetta was the abundant molecular oxygen observed in the coma (Bieler et al. 2015b), while circumstantial evidence for O_2_ was also found in comet 1P (Rubin et al. 2015). Several formation mechanisms have been discussed, from radiation of water ice and cold temperature chemistry in the ISM, to various *in situ* formation mechanisms. Comet Interceptor will assess how ubiquitous O_2_ is in comets: a more pristine LPC or DNC target will narrow down possible formation mechanisms (cf. Luspay-Kuti et al. 2018). Evidence of ammonium salts in 67P found by Rosetta (Altwegg et al. 2020) was also unexpected and may change the traditional view of species parentage and how molecules are stored in cometary ices; for example, NH_4_CN (if present in the coma) can produce NH_3_ and HCN, which were previously believed to be “parent” molecules released directly from the nucleus.

Isotopic ratios, being very sensitive to physico-chemical conditions, provide crucial information on the provenance of cometary material, and therefore provide information for comet and planet formation models. For instance, the D/H ratio in cometary water has been used to infer whether or not comets could be a source of the water on Earth (Hartogh et al. 2011). To date, all comets with known D/H either exhibit a terrestrial ratio or an elevated ratio (Altwegg et al. 2017b). A recent study suggests two distinct sources of water with different D/H: one source on the surface of the nucleus and the other in the form of sublimating icy grains in the coma (Lis et al. 2019). Fulle (2021) models these observations with ‘pebbles’ of different ice content. Depending on the type of the activity of the target comet, Comet Interceptor may obtain measurements of both reservoirs during the flyby. The D/H ratio of a less evolved object may furthermore give some insight into outgassing-related fractionation processes. These measurements will also help in interpreting differences or similarities between remote and *in situ* D/H measurements, in particular if simultaneous measurements can be made from the ground, which is likely to be the case if Comet Interceptor’s target is a relatively bright LPC, as expected. Measurement of the ^18^O/^16^O in H_2_O by Rosetta revealed an enrichment in 18O compared to the terrestrial value (Altwegg et al. 2019). Models of chemical evolution in the protoplanetary disc or natal molecular cloud of our Solar System do not yet explain these results, and demonstrate the value of isotopic measurements in furthering our understanding of the topic of planetary system formation in general (Hily-Blant et al. 2017; Wirström and Charnley 2018; Furuya and Aikawa 2018). Even though D/H is the primary objective, if the target is sufficiently active, Comet Interceptor will investigate isotopes in other species including, e.g.: ^18^O/^16^O in H_2_O, ^13^C/^12^C in CO_2_, and ^34^S/^32^S in OCS and CS_2_.

Many pre-solar signatures have been observed in the isotopes of volatiles in 67P (Hoppe et al. 2018). Similar bulk abundances of volatile molecules were observed in Comet C/1995 O1 Hale-Bopp and 67P, and within objects in the ISM (Bockelée-Morvan et al. 2000; Drozdovskaya et al. 2019). The detailed *in situ* measurement of an LPC or a DNC will probe the potential locations of origin of its ices at a molecular, elemental, and isotopic level.

##### Spatial Distribution and Structures of the Neutral Gas Coma

**Fig. 9:**
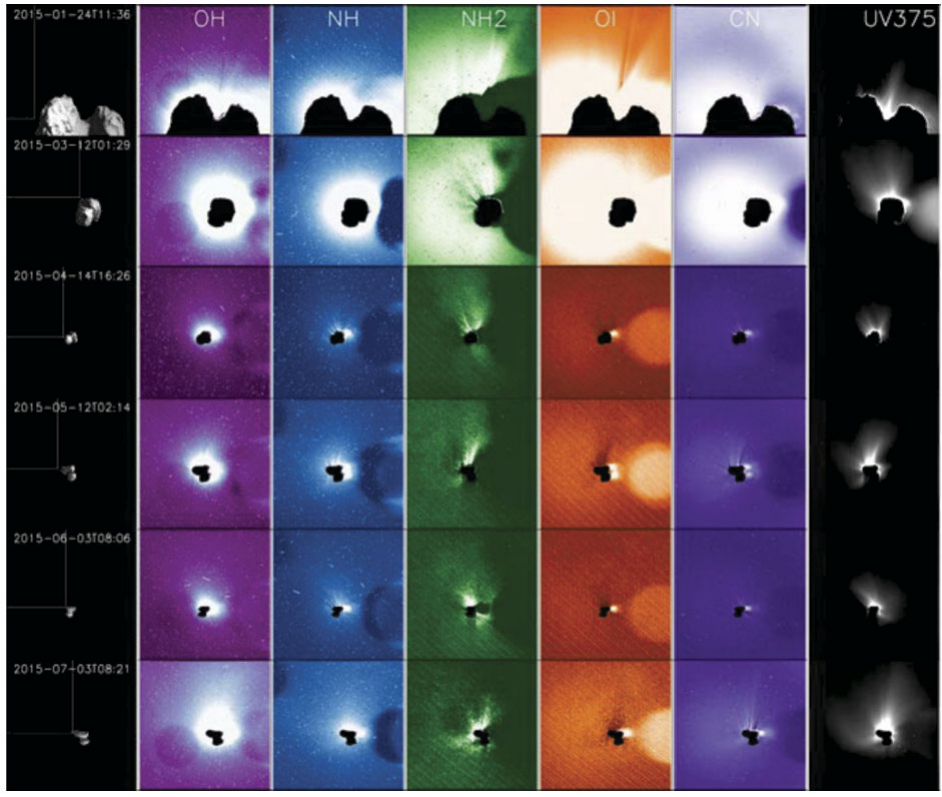
Images showing different morphology of gas jets of different species from Rosetta/OSIRIS (from Bodewits et al. 2016)

The spatial distribution of volatiles in the coma is of particular interest as it can provide information on how the ices are distributed in the nucleus. However, this aspect is a difficult issue to address. Ground-based observations only allow mapping of the distribution of volatiles on very large scales, missing the crucial transition region between the nucleus and the inner coma. As Rosetta orbited the comet for an extended period, it built up maps of coma composition of mother and daughter species at much smaller scales above different areas (Fig. 9). Rosetta confirmed that comets have heterogeneous comas dominated by large fluctuations in composition, often linked to diurnal and seasonal variations in the major outgassing species such as H_2_O, CO_2_, and CO (e.g., Hässig et al. 2015). A general large-scale anticorrelation between H_2_O and CO_2_ was observed by Rosetta (e.g., Mall et al. 2016; Migliorini et al. 2016). The gas density in the coma is strongly affected by nucleus concavities and sun illumination conditions, even when the distribution of ices on the nucleus surface is quite uniform (Bieler et al. 2015,b).

Separate but simultaneous measurements from spatially distributed sub-spacecraft will provide snapshots of different coma regions at the time of flyby. This method actually has advantages over Rosetta’s approach, as it will allow, for the first time, separation of spatial and temporal variations. Interpretation of Rosetta results instead requires complex models to understand how the comet changed with time and space, because measurements of different coma areas were taken at different places and times along Rosetta’s orbit. The unique multi-spacecraft architecture of Comet Interceptor will allow the addressing of important questions on how dynamic effects in the coma relate to each other: Which are due to the changing position of the spacecraft relative to the nucleus, and which to the comet’s time-varying behaviour? (cf. Hansen et al. 2016).

##### Dust Coma

The refractory component of the comet coma is referred to as ‘dust’. It is made up of minerals and organic components lifted from the surface with sizes ranging from sub-micron to metre-scale chunks. The properties of the dust are important to understanding comet composition and formation. For example, the similarity in size of the dominant particles in 67P’s coma and those predicted to form planets via streaming instability was used to argue in favour of that model, assuming that particles reflect the original size distribution (Blum et al. 2017). Measuring the dust size distribution down to nm size in an LPC will provide some information towards testing the universality of this assumption: if it is similar to JFCs it would imply a link with primordial dust distribution, and support streaming instability models, whereas a difference would imply that comet material has been processed at μm scales and that dust properties reveal evolutionary processes.

Whether or not solid material lifted from the comet’s surface also contains ice is important for our understanding of activity processes, as sublimation from dust in the coma could form a distributed source of water or other gasses. Rosetta found that little water is provided by sublimation from particles beyond a few nucleus radii, so there is no significant distributed source for water at 67P. That does not preclude distributed sources for minor constituents: for instance, evidence has been found for a distributed source for the hydrogen halides (De Keyser et al. 2017) and some of the lesser volatiles including organics (Altwegg et al. 2016, 2017a). This is in stark contrast to the earlier EPOXI flyby of comet 103P (A’Hearn et al. 2011) and remote observations of the innermost coma of comet 73P/Schwassmann-Wachmann 3, where a significant fraction of the outgassing occurred from a distributed source of icy grains (Fougere et al. 2012, 2013). Still, assessing the existence of a distributed source is difficult, even during a fast flyby where one can consider gas and dust production to be basically constant: cometocentric longitude, latitude, and distance all change simultaneously, and therefore it is not straightforward to extract purely radial density profiles from which one can ascertain the existence of a distributed source. To detect and identify distributed sources, it is necessary to scan a very large range of radial distances and, simultaneously, to assess the dust and gas abundances at a minimum of two points in the coma: again, the multi-point architecture of Comet Interceptor will give it a unique advantage in addressing this question. Constraining the dust-to-ice ratio in the coma (individually for icy particles, or in bulk by comparison of dust and gas production rates), and by inference in the nucleus, will also provide information for planet formation models. The question of how much ice is present in larger chunks in the coma is a critical parameter to understand the overall dust-to-ice ratio within a comet, which is a very important number to constrain the properties of the formation location of the comet, and one that is still the subject of intense debate following Rosetta (see, e.g., Fulle et al. 2019; Choukroun et al. 2020). Measurements at a less evolved LPC would be very valuable to understand this intrinsic ratio.

##### Dust Reflectance Properties

**Fig. 10:**
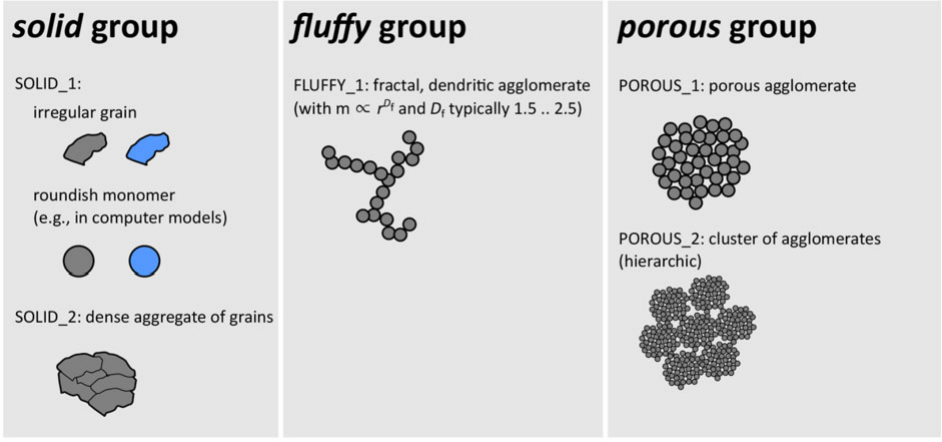
Morphology of different types of dust particles, from summary by Güttler et al. (2019)

Ground-based observations of the intensity, colour, and degree of linear polarisation of light scattered by cometary dust particles are used extensively for retrieving information on their physical properties, including their morphology and structure, as defined by Güttler et al. (2019) (Fig. 10). However, they are usually limited to certain observational geometries. Observations from spacecraft offer the opportunity to sample a wider range of geometries at a single time, from within the comet’s coma. The analysis of the Rosetta dataset has exposed challenging contradictions between the properties of cometary dust as modelled from ground-based and from *in situ* observations. The Rosetta OSIRIS camera system provided unique observations of the intensity of light scattered by dust within 67P’s coma (Bertini et al. 2017). The observed phase functions show a peculiar U-shape with a minimum at a phase angle around $100^{\circ}$ (compared with the minimum at ${\sim} 55^{\circ}$ in the Halley-Marcus function. Those data would indicate the presence of large grains. Further, ground-based observations of the degree of linear polarisation of 67P show a negative polarisation branch (NPB) at small phase angles and a maximum observed DLP (Degree of Linear Polarisation) of ∼8% at a phase angle of $32^{ \circ}$ obtained after the 2015 perihelion (Myers and Nordsieck 1984; Chernova et al. 1993; Hadamcik et al. 2016). Micron-sized particles may reproduce the shape of the DLP curve, but certainly not the U-shaped OSIRIS phase function The major challenge is reconciling conclusions about the properties of cometary dust obtained from the analysis of both datasets, which demands a common framework to interpret consistently all the datasets available (Moreno et al. 2018; Markkanen et al. 2018; Levasseur-Regourd et al. 2019; Muñoz et al. 2020).

Rosetta did not provide *in situ* polarimetric observations, but these were obtained by Giotto’s HOPE (Halley Optical Probe Experiment) instrument, which took observations along the line-of-sight of the spinning spacecraft at different wavelengths free of gas emission. These allowed an estimate to be made of the density of the cometary dust, which was found to be low (about 100 kg m^−3^), and of its geometric albedo (0.04, matching that of the nucleus). The polarisation was highest when crossing dust jets and lower in the inner coma region. During the Giotto Extended Mission flyby of 26P/Grigg-Skjellerup, HOPE hinted at the presence of pebbles ejected from the nucleus. Since then, polarisation imaging of active comets have confirmed the existence of lower polarisation in the innermost coma and increased polarisation along large-scale dust jets. Interstellar comet 2I/Borisov presented unique polarimetric features: the polarisation increased steeply with phase angle, reaching values substantially higher than typically measured in small Solar System bodies (Bagnulo et al. 2021). These polarimetric properties distinguish 2I from dynamically evolved objects such as JFCs, and suggest that 2I was a highly pristine object, with a coma probably characterised by relatively small-sized aggregates. This also suggests that detailed *in situ* polarimetric observations at a DNC or LPC will be very valuable in revealing differences in dust properties between pristine and evolved comets.

##### Dust Structure and Distribution

Numerical simulations based on the reflectance properties described previously conclude that dust particles are irregular, porous, built of smaller grains and possibly fractal in nature. Additionally, experimental simulations under different conditions (including microgravity) suggest the presence of fluffy aggregates with comparable amounts of minerals and organics (Levasseur-Regourd et al. 2015). This agrees with the results from Rosetta’s *in situ* sampling, in particular microscopic imaging of grains by the COSIMA (Langevin et al. 2020; Kimura et al. 2020) and MIDAS instruments (Mannel et al. 2019).

*In situ* measurements (typically measuring dust flux from momentum transferred in collisions with a target on the spacecraft) naturally sample only a single coma location at a time. Rosetta’s GIADA measured grains’ cross-sectional area, velocity, and impact momentum. It could therefore separate fluffy and compact particles. The instrument also characterized the submicrometre- to micrometre-sized dust mass flux at 67P, finding a differential size distribution index of ${\approx} -3.0$, which confirms that particles of size ≥0.1 mm dominate the dust coma cross-section during that comet’s entire orbit (Della Corte et al. 2019). On flyby missions, the velocity of impacting particles is dominated by the relative spacecraft velocity to the comet, which is well known in all cases, so particle masses can be directly inferred from momentum sensors, as achieved, for example, by instruments on the Giotto and the Stardust mission (the latter also delivering a sample of the more solid coma grains to Earth). Comet Interceptor will be the first mission to make such measurements at two coma locations simultaneously, which will be very valuable for understanding the evolution of particles (e.g., any evidence of fragmentation) flowing away from the nucleus.

The spatial dust distribution within the coma is important for understanding activity, i.e., how, and where the dust flows away from the nucleus (see next section), and can be divided into the inner coma, larger scale coma, and tails. Both remote sensing and *in situ* measurements reveal significant variation in the population in different regions. There are broad outflows and more collimated jets, which appear to be controlled by nucleus topography in the inner coma, but are also visible on 1000s of km scales in ground-based imaging. The large-scale features have yet to be conclusively linked to the inner coma structures and nucleus. At the very largest scales, dust is swept into the characteristic dust tail, where differences in acceleration due to solar radiation pressure differentiate the material largely by particle size – models of tail morphology can therefore be used to place constraints on particle properties.

The fast flyby will provide an instantaneous snapshot of the nucleus and coma. However, depending on the comet’s spin state, pre- and post-closest approach observations will contribute additional information on the diurnal evolution of the near/far environment. Such observations, even with an unresolved nucleus, will be extremely valuable to characterize the gas and dust distribution, as well as the activity of different regions at multiple local times.

#### Activity

**Fig. 11:**
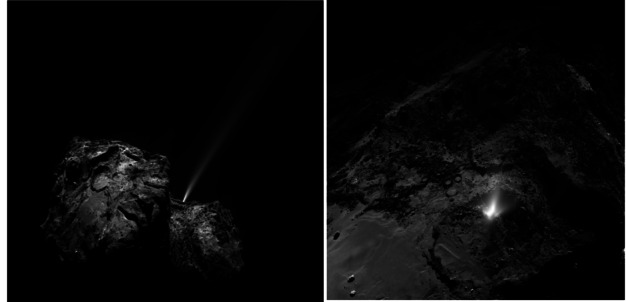
Outburst on the surface of 67P/Churyumov-Gerasimenko observed on 3 July 2016 (left) and on 29 July 2015 (right). ESA/Rosetta/MPS for OSIRIS Team MPS/UPD/LAM/IAA/SSO/INTA/UPM

**Fig. 12:**
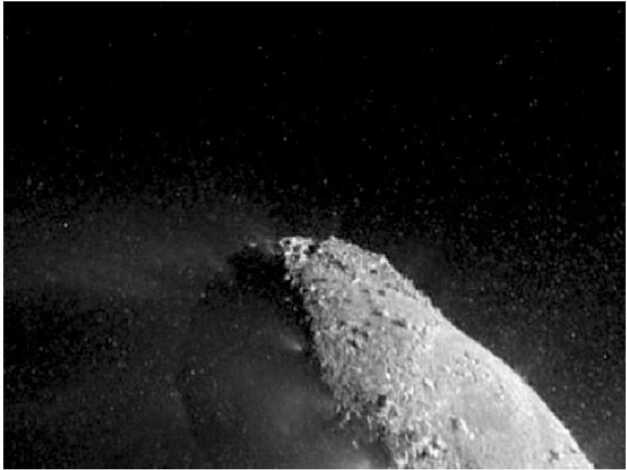
Image from NASA’s EPOXI mission shows part of the nucleus of Comet 103P/Hartley 2. The sun is illuminating the nucleus from the right. A distinct cloud of individual particles is visible, gas release from which is responsible for the high apparent activity level given this nucleus’s size. Image Credit: NASA/JPL-Caltech/UMD

Cometary activity is a complex process: the exact sources and mechanisms driving activity in comets remain a puzzle, e.g. Keller and Kührt (2020). Rosetta revealed that cometary activity, jets, and outbursts (Fig. 11) are linked to distinct morphological nucleus features (Vincent et al. 2015, 2016). Working activity models have been recently provided in case of pebble-made nuclei (Fulle et al. 2020a,b; Gundlach et al. 2020; Fulle 2021; Ciarniello et al. 2021, 2022). The pebble model is however debated in the community, as the existing data is not sufficient to fully assess its validity. By characterizing an LPC, in particular a DNC, from a close distance, Comet Interceptor will obtain unique observations of dust and gas release from a type of object not studied before and provide a test case to evaluate all current cometary activity models. The surface of a DNC on its first approach to the Sun is expected to be little processed and Comet Interceptor will allow us to assess how this impacts activity. If activity can be attributed clearly to local areas in a DNC this would offer strong evidence that they are due to primordial shape or composition heterogeneities, and not evolution-driven features, as no processed surface crust is expected. Evidence for or against hyperactivity – significant activity being driven by sublimation from icy ‘chunks’ in the coma (Fig. 12) – would test also whether or not this phenomenon (seen in 103P but not in 67P) is evolution-related, and may be important in the interpretation of ground-based observations of other DNCs. Typically, the distribution of coma gas and dust structures in the near environment and their nucleus source locations are inferred from the spatial tracking of jet-like emissions across multiple images. The combined motion of the spacecraft and rotation of the nucleus usually provide sufficient geometric variation to reconstruct the jets’ 3D shape. This method, however, assumes that tracked features do not evolve between images, which may not always be the case. By providing multiple views of the same features from different angles at the same time, Comet Interceptor will remove this uncertainty and allow for a more precise mapping of apparent active regions.

The most obvious observational evidence of nuclear activity is the collimated dust jets ejected from the surface. There is strong complementarity between *in situ* and ground-based imaging of coma structures, as was shown for 67P, where the analysis of ground based data revealed the presence of structures originating from certain latitude/longitudes on the nucleus (Vincent et al. 2010, 2013; Lara et al. 2011; Marschall et al. 2016). This allowed the nucleus’s rotational period to be derived, based on previous apparitions, before the spacecraft arrived. These first results related to 67P’s spin period and activity were confirmed by Rosetta, although the mission’s findings go beyond the classical view of active areas on a nucleus surface producing jet-like features. Comet Interceptor’s three viewpoints will provide the data necessary to link nucleus surface activity and topography with the large dust structures in the coma. Additionally, ground-based observations of the target’s activity should be plentiful and high signal-to-noise, for a relatively bright LPC target; these will complement and connect to the multi-point spacecraft measurements.

#### Plasma Environment and Interactions

The neutral gas released from a comet eventually becomes partially ionised through exposure to solar UV radiation, and/or energetic particle impact bombardment. Ions born close to the nucleus interact with the cometary neutrals and, for a high enough outgassing, a rich range of chemical reactions can take place, producing new ion species, e.g. Beth et al. (2020). These fresh cometary ions come under the influence of the heliospheric magnetic field, join, as pick-up ions, the solar wind flow through and past the comet, and are carried downstream, forming the visible ion or plasma tail.

##### Origin and Overall Structure of the Solar Wind Interaction Region

The ion cloud around a nucleus presents an obstacle to the solar wind (Alfven 1957; Biermann et al. 1967). A comet’s plasma environment is heavily dependent on the number of cometary ions in the coma, and therefore the gas production rate. As the cometary pick-up ions are incorporated into the solar wind – a process known as mass-loading – the flow around the comet changes, which modifies the cometary and solar wind plasma. In the cometary reference frame, pickup ions are initially almost at rest, there is thus a large discrepancy in velocity between the two flows. Mass loading simultaneously slows the solar wind and accelerates cometary ions. A bow shock forms where the solar wind adjusts abruptly to the comet’s presence. This also has consequences for the magnetic field, as it is largely frozen into the flow, and starts to drape around the obstacle.

##### The Value of Multi-Point Measurements

A multi-spacecraft encounter would provide a significant advantage over previous missions in that it would sample different paths through the comet-solar wind interaction region. This includes both the largest scales, beyond cometocentric distances explored by Rosetta, and the smaller scales, nearer the nucleus. Rosetta showed how strongly structured and dynamic the cometary plasma environment is, and how different processes affect different parts of the comet magnetosphere for different activity levels. Charge separation, shocks, and other boundaries, as well as the excitation of waves and plasma heating, all affect the exchange of energy, momentum, and mass on multiple scales simultaneously. This in turn is important for processes such as the sputtering of the nucleus surface and coma dust with energetic solar wind and cometary ions (Wurz et al. 2015), for the excitation of coma constituents and associated emissions (Galand et al. 2020), for coma chemistry, and for the formation of the comet ion tail and its rich structure. Rosetta lacked a larger scale overview and understanding of the 3D structure of the induced magnetosphere and magnetosheath. Earlier flyby missions, such as Giotto, had the opposite issue: they provided measurements over very large scales, but only along a single path.

**Fig. 13:**
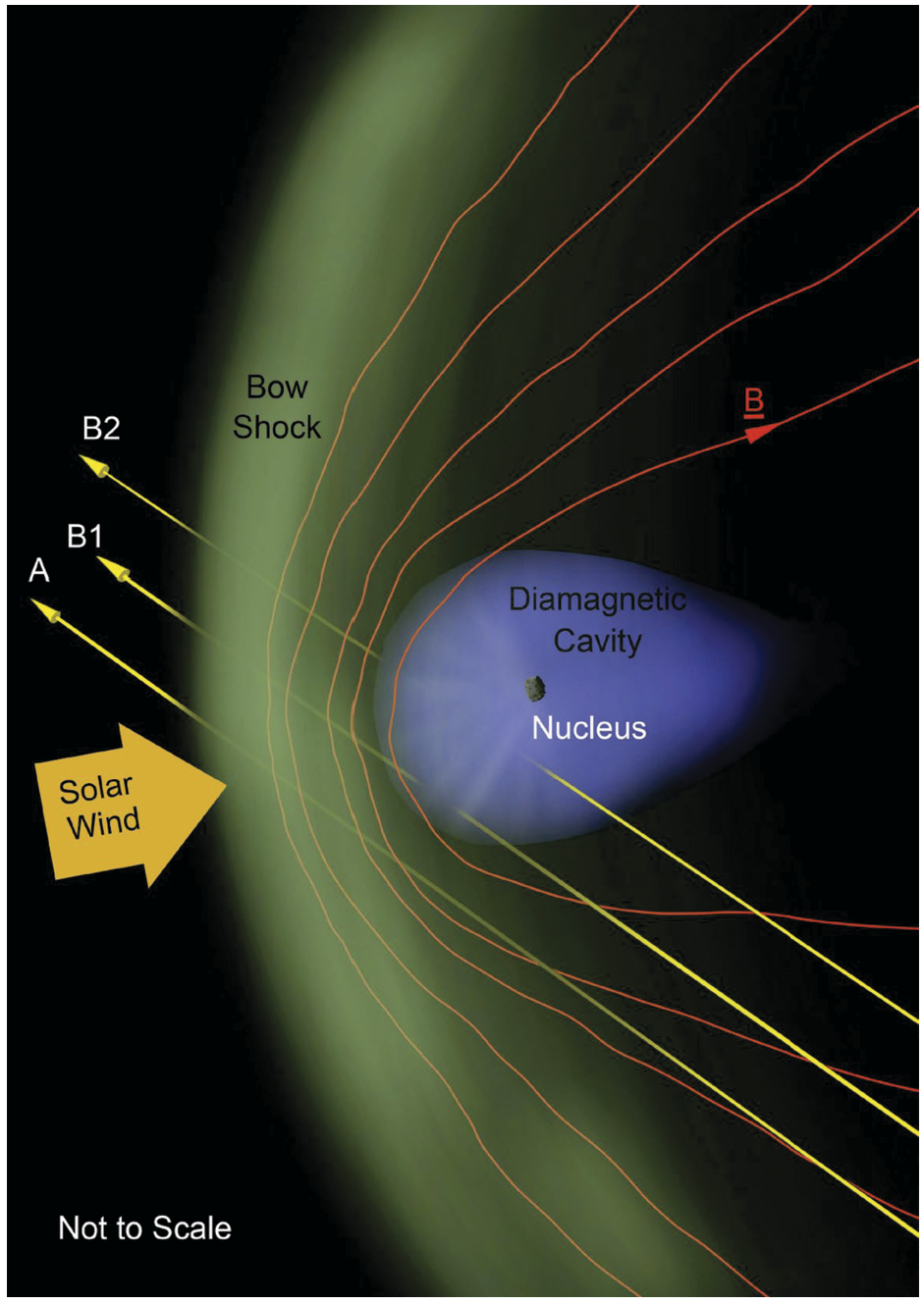
Multi-point measurements will determine the scale and shape of several structures in the comet-solar wind interaction. The time at which each of the three spacecraft/probes cross (or do not cross) the bow shock (green) and diamagnetic cavity (blue) will determine their shapes and scales. The magnetic field (red) will also be probed using magnetometers on all the three platforms

With Comet Interceptor we therefore expect to observe, along multiple chords (Fig. 13), a shock, the diamagnetic cavity, strong electron heating, penetrating solar wind ions, energetic cometary ions moving towards the nucleus, and strong wave excitation. These measurements are needed to assess the relative importance of different mechanisms and to understand how the solar wind affects the comet environment for different types of objects and levels of comet activity. The 3D picture is needed to distinguish between different solar wind-coma interaction models. This can only be properly investigated with a multi-spacecraft mission, in order to reduce the spatio-temporal ambiguities introduced by potentially changing solar wind conditions during the flyby.

##### Specific Features of Interest

Bow shocks haves been observed in several comets and modelled extensively, e.g., Koenders et al. (2013). In general, this shock moves outwards in the upstream solar wind, away from the nucleus, as the neutral gas production rate increases, with standoff distances up to millions of km upstream at high gas production rates (as for 1P). At low production rates, the critical point is never reached, and no bow shock forms. For example, Giotto’s 1992 flyby of 26P revealed a bow shock during the outbound trajectory, but not inbound (Coates et al. 1997). Instead, it detected a bow wave (e.g., Scarf et al. 1986), a more gradual field increase rather than the jump-like classical shock. This was attributed to the low Mach number of the mass- loaded solar wind flow. At Halley, the bow shock as observed by Giotto outbound was quasi-parallel (Neubauer et al. 1990). At 67P, Rosetta’s trajectory did not allow for the detection of a bow shock or wave. At lower gas production rates, a feature interpreted as an infant bow shock, a highly asymmetric structure behaving like a shock and confined to one side of the interaction region (Gunell et al. 2018) was observed in the plasma environment. Spectral breaks in the pick-up ion energy distributions have furthermore been interpreted as indirect proof of a bow shock far upstream of the spacecraft (Alho et al. 2019, 2021). Modelling also revealed that solar wind charge exchange, as well as asymmetric nucleus outgassing plays a major role in the dynamics, width, and extent of the shock (Simon Wedlund et al. 2017; Alho et al. 2021). As a consequence, measuring the specifics of the shock (or wave) at different locations, or finding that there is a shock- like feature at one spacecraft and a wave- like behaviour at another, brings invaluable information on the solar wind-comet interaction. Depending on the flyby velocity and measurements’ sampling rate, unprecedented multi-point information could be obtained on the shock’s internal structure.

The draped magnetic field threads through the comet’s coma and ion tail. However, the solar wind magnetic field varies in both strength and direction. Therefore, layers of magnetic field with different directions are embedded in the induced magnetosphere; structuring referred to as “nested draping”. This has been observed during the fast flybys of comet 1P, for which a tentative model of how the magnetic field lines on either side of the nucleus are connected could be made (Raeder et al. 1987). Performing a flyby with magnetometers on three spacecraft at different distances will allow the first reconstruction of the shape of the draped magnetic field. Three chords through the upstream induced magnetosphere would deliver three lines of magnetic field strength and direction that can be integrated into a whole. If the flyby is slow, dynamic draping can also be observed, first shown by Rosetta (Volwerk et al. 2019). When the convection velocity of the magnetic field is faster than that of the spacecraft past the comet, then the time variation of the magnetic field between the spacecraft can show how the nested draping is moving towards the comet. It was also shown that the magnetic field draping is not confined to the solar wind magnetic field plane, but can be shifted (Koenders et al. 2016).

In strongly outgassing comets, the solar wind-comet interaction forms a region immediately surrounding the nucleus called the diamagnetic cavity. This cavity is devoid of a magnetic field and prevents the solar wind’s full penetration into the region closest to the nucleus. First observed in an artificial comet (through a barium release in the solar wind just outside the Earth’s magnetosphere (Bernhardt et al. 1987), this cavity was also found at 1P by Giotto, extending up to 4000 km from the nucleus. At 67P, Rosetta repeatedly detected the cavity, which extended only a few 10s to 100s km from the nucleus. The size of this magnetic field-free region depends on the activity of the comet, and with 67P being much less active than 1P, the cavity size was accordingly much smaller. Due to the long residence time of Rosetta at 67P, it was found that the cavity boundary is very dynamic, moving in and out but, with only one spacecraft only limited information on the boundary could be obtained.

The exact mechanism that sustains the diamagnetic cavity is not understood and more measurements are therefore needed; its boundary has been observed only at 1P and 67P. The plasma in the cavity is much quieter than outside it and is more dominated by cold electrons (Odelstad et al. 2018). The stability of the boundary appeared to differ between the two comets (Neubauer 1987; Goetz et al. 2016), and only rough estimates of the boundary velocity could be derived.

The boundaries between different regions are asymmetric and highly variable in both space and time. They depend on both the comet’s activity and variations in upstream solar wind conditions. Edberg et al. (2016b,a), Hajra et al. (2018), and Goetz et al. (2018) showed that drastic changes occurred in the plasma at 67P, when interplanetary coronal mass ejections or co-rotating interaction regions passed the comet. To advance our understanding of the physics of these interactions, we need to go beyond what was possible with previous missions, all of which could only measure at one location at one time, by measuring at multiple positions simultaneously, allowing the separation of spatial and temporal changes. Whether one, two, or all three of the Comet Interceptor platforms will enter the diamagnetic cavity, will on its own give information on the cavity’s size and shape, as well as indications of its dynamics, and will be expanded upon by other measurements.

Solar wind charge exchange produces cometary ions as well as energetic neutral hydrogen and helium that can be observed remotely through ENA imaging (Simon Wedlund et al. 2016, 2017, 2019c,a,b): their presence testifies to the efficiency of energy and momentum transfer between the solar wind and the cometary coma. Observations of ENAs are also useful to characterise plasma interactions in the region where the solar wind reaches deepest into the neutral atmosphere (Ekenbäck et al. 2008), possibly partaking in the sputtering of the nucleus’s surface (Nilsson et al. 2015). Ion chemistry within the coma also depends on neutral gas composition and, therefore, ion observations can shed light on the bulk composition of the nucleus, and the still poorly understood complex chemical and photo-ionisation reactions in the coma (Haeberli et al. 1995; Fuselier et al. 2016; Heritier et al. 2017; Beth et al. 2017).

Waves take on an important role in the cometary plasma environment, transferring energy across boundaries and heating particle populations through wave-particle interactions. The plasma environment of a comet is a complex mix of ions of different species and origin and relative velocities, electrons of different temperatures, neutral molecules and dust particles of different sizes and charge states. Solar wind interactions with the cometary plasma gives rise to instabilities that drive waves of various kinds, including ion-cyclotron and/or mirror-mode waves, e.g., Mazelle et al. (1991), harmonic waves created by the ion- Weibel instability (the “singing comet” waves found by Rosetta; Weibel (1959), Richter et al. (2015), Meier et al. (2016), Glassmeier (2017)), lower hybrid waves, e.g., Karlsson et al. (2017), and ion acoustic waves, e.g., Gunell et al. (2021). It is, however, not clear in which region of the coma these waves are present and how they depend on comet activity. Through multipoint measurements in the coma one can determine, in principle, the temporal and spatial development of the waves. Going from single spacecraft to multi-spacecraft observations thus enables new insights into both the physics of the waves themselves and how they affect boundaries and the surrounding plasma.

**Fig. 14:**
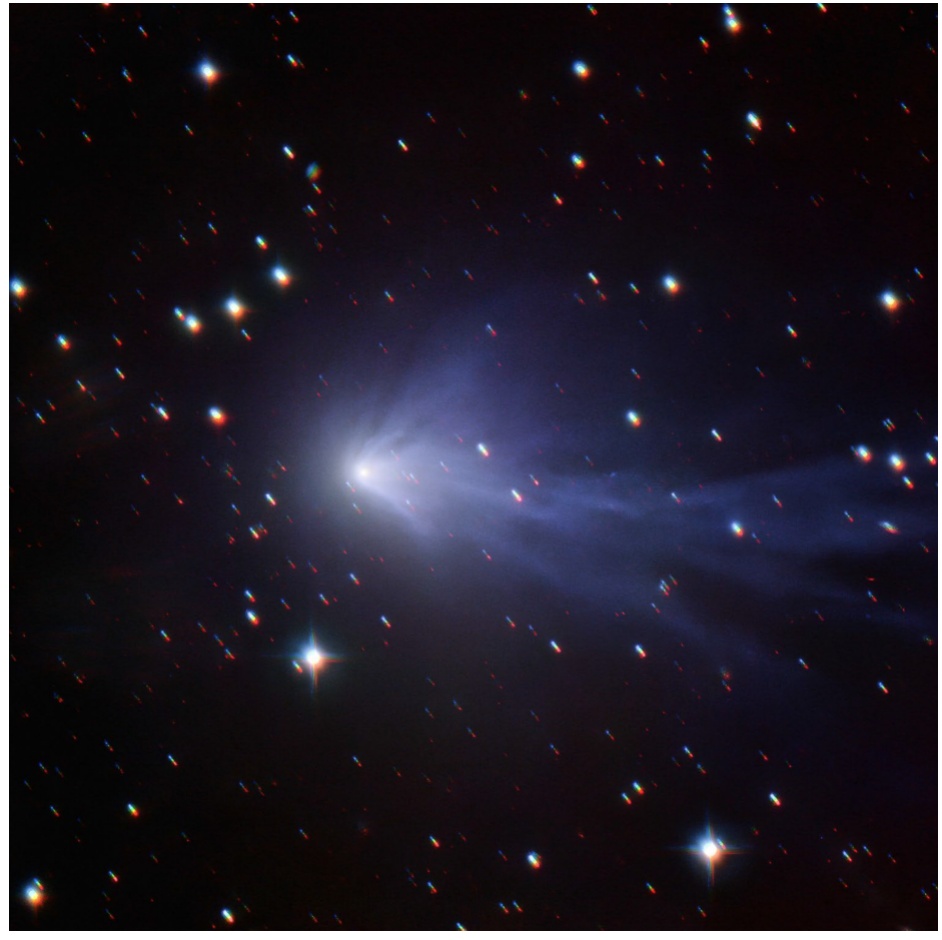
The highly-structured ion tail of Comet C/2016 R2 (Pan-STARRS). This particular comet was an atypical dust-poor LPC (e.g. Biver et al. 2018) which reached perihelion at 2.6 au from the Sun. Image: ESO, under license Attribution 4.0 Interactional (CC BY 4.0)

The comet-solar wind interaction region can also be observed remotely, due to resonance fluorescence processes occurring in common ions such as CO^+^ and H_2_O^+^. The structures observed in the ion coma and tail reveal the spatial distribution and motion of cometary ions, e.g., Fig. 14. Coma and near-tail ions could be observable with Comet Interceptor’s cameras, providing complementary observations of ion structures and their dynamics, especially during the approach to the comet. The rate of motion and relative densities of these structures can be compared to the *in situ* observations, providing ground truth data for remote plasma observations from Earth.

## Scientific Objectives and Requirements

### Overview

In Sect. 2, we summarized the scientific context of the Comet Interceptor mission. The many unanswered questions regarding the potential targets provide the strong rationale for a multi-platform mission to an LPC, ideally a DNC, together with great advances in the understanding of solar wind interactions with comets in general. Here we identify the key objectives to be addressed by the mission, and the measurements that are needed to achieve them.

The multiple spacecraft approach of Comet Interceptor means that its target comet will be observed from different angles during the flyby, building up a 3D picture of the nucleus, coma, and interaction with the solar wind. This will allow the separation of differences in time (e.g., due to the changing activity of the comet) and in space (e.g., due to inhomogeneities in the outgassing pattern). Such a separation has not been possible in any previous flyby mission, or even with Rosetta, which could only sample one location in the coma at a time.

**Fig. 15:**
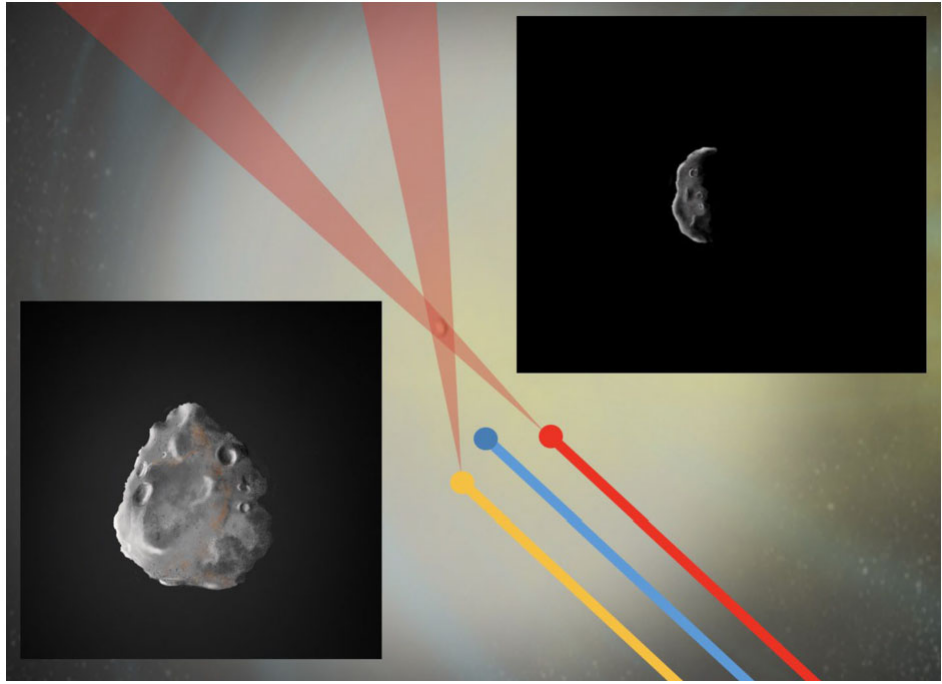
The remote sensing instruments aboard all three platforms will return complementary views of the nucleus from different directions. Inset images show representative complementary views of the single nucleus from two of the three spacecraft platforms

Concerning nucleus science, Comet Interceptor will measure the size, shape, and rotation rate of the target comet nucleus. It will return resolved images of the surface that reveal its morphology. The nucleus composition will be constrained directly via remote sensing observations (imaging, spectroscopy, and polarimetry). Furthermore, Comet Interceptor will measure directly, for the first time at a comet, nucleus thermal properties via thermal infrared imaging. Visible and near-infrared images of the nucleus will be returned from three different viewpoints, as illustrated in Fig. 15: from the main spacecraft (A) and both probes. The highest resolution images should have a resolution of ∼10 m / pixel, comparable to previous comet flyby missions, allowing direct comparison of an LPC with the more evolved short period comet nuclei imaged previously.

Data will be captured through the flyby from a wide range of phase angles, further allowing the reflectance properties of the nucleus (and different resolved regions on it) to be assessed. The variety of viewpoints, and the possibility to constrain the unilluminated portions of the nucleus through a combination of thermal imaging and imaging of its silhouette against the background coma, will give good constraints on the size and shape of the entire nucleus, even if, due to the flyby nature of the mission, detailed imaging can only be returned from one side. The nucleus rotation rate will be constrained by both resolved imaging and images of the unresolved nucleus in the days before and after the flyby.

A mission to a relatively pristine LPC will be an important advance in cometary science as a whole, as direct measurements of the coma composition can be related to nucleus ices with a very different processing history, and the distribution of active areas can be studied. Mapping the distribution of neutral gasses in the coma will give information on bulk composition and nucleus inhomogeneity and will probe coma chemistry. Measuring the composition of the coma at different distances from the nucleus will provide information for coma chemistry models. Remote sensing of the large-scale distribution of different species will be combined with *in situ* sampling to derive production rates of the individual volatile species for the time of the flyby (for comparison to ground-based observations). Identification of parent and daughter neutral and ion species will enable an assessment of their relationship in the coma. All of these individual elements will combine to give a comprehensive picture of the similarities and differences between comets with different evolutionary histories. The spacecraft measurements are critical: while we have ground-based observations of many comets of different classes, it is unclear what differences are evolutionary versus inherent. Comparison of the direct inner coma measurements at an LPC with previous JFC missions, in particular Rosetta, will help disentangle coma processes and long-term evolutionary trends.

Comet Interceptor will detect small-scale structures within the coma, <1 km in scale for a slow flyby, through *in situ* measurements of dust flux and gas density, along with remote sensing imaging from cameras on all platforms. Such an analysis, combined with nucleus shape observations, will supply information about the complex coma-nucleus relationship. For a bright LPC, measurements of even larger-scale spatial distribution of species in the coma will be possible from ground-based observatories and will provide, for the very first time, a clear link between the features observed from the ground and the nucleus of a comet.

Mapping of the dust and neutral gas jets both near to and far from the nucleus, especially on approach and post-encounter, where dust jet and shell structuring can constrain the time history of nucleus activity, will reveal more active locations and periodicities in the ejection rate of material. The jets should be mappable to their source locations, tying into high resolution images of the nucleus. The composition of the gas jets can also reveal a considerable amount about the active regions, such as determining whether all active regions have the same composition, and whether or not the relative abundances of different ices vary across the body. Observations in the IR will isolate jet emission by H_2_O, CO_2_, and CO, but also typically less abundant species such as methane, ethane, and methanol. Spectral information about the coma, and, better still, spectral imaging, will be highly beneficial. The spatial distribution of neutral hydrogen would provide a time history of activity in the comet; this could be achieved with imaging of the Lyman-$\alpha $ line in the UV range. The composition of the coma can be measured directly using *in situ* observations by a mass spectrometer. Coverage of masses up to a few hundred Daltons (amu) would be particularly beneficial. More direct inferences about surface activity can be made using thermal maps of the nucleus. For these, the surface would need to be resolved to better than ∼300 m for a 3 km-wide body.

Measuring *in situ* absolute densities of major neutral gasses and comparing with ground-based observations will allow coma models used to deduce production rates from ground-based observations to be tested and constrained. This is crucial to interpret the wealth of ground-based data using a common baseline. These measurements will also allow Comet Interceptor to help investigate the contribution of distributed sources and the pathways leading to different gas species and their complex relation with the bulk composition. Mass spectroscopy will also reveal minor constituents of the gas coma, and isotopic ratios of D/H if the gas production rate is sufficiently high (and possibly the most abundant isotopes of O, C, and S), allowing detailed comparison of composition differences and/or similarities between a less evolved comet and results from Rosetta at 67P.

Observations of all-sky brightness within the coma, and polarimetric curves, will allow us to study the observed phase function curve and the degree of linear polarisation from the same data set, including the important forward- and back-scattering regimes. The combination of both datasets will yield key constraints for the physical properties of the dominant population of dust particles in the coma, alongside the dust mass distribution measured along two different trajectories. A detailed characterisation of the plasma environment of a comet requires measurements of the magnetic and electric field strength and direction, the electron and ion distribution functions (density, temperature, and flow velocity vector), and the ion composition, in the different comet-solar wind interaction regions (e.g., solar wind, diamagnetic cavity) and at their boundaries.

Comet Interceptor will assess, through sensors on spacecraft A and B2, and the B1 Plasma Suite, the energy, mass, and momentum transfer in the cometary environment, through the coma and across boundaries. Multi-point measurements of ions, magnetic fields, and nm- to mm-scale dust will elucidate the physics behind mass transfer and the consequences for both the coma and tail. Unprecedented ENA observations will help us to understand the role of charge exchange collisions in the transfer of energy and momentum from the solar wind to the coma. Solar wind and cometary ion and electron dynamics will enable the assessment of the amount of electrically-charged material impacting the (pristine) comet surface.

For the magnetometers on all three platforms on this mission, the bow shock (or bow wave), the field-line draping pattern around the inner coma, and the diamagnetic cavity, are of specific interest, amongst other features. Using the spatial separation of the three spacecraft and their magnetometer measurements, one can deduce the three-dimensional shape of the magnetic field, and study the differences in magnetic activity. The magnetic field measurements also play a defining role in analysing the internal structure and type of the plasma boundaries. Given the possibly high flyby speed, the planned high cadence measurements of the most prominent plasma properties (ion and electron density, electron temperature) are essential to capture as much as possible of the detailed spatial and temporal structure of the plasma. This will help in particular in studying the internal structure of the boundaries (shape, spatial extent, etc.). The ion coma and ion tail in its vicinity will be observed by the visible light cameras on all three platforms.

### Science Objectives and Requirements

The above summary of the most desirable measurements possible during a fast flyby and modest spacecraft size are formalized as Science Objectives and Requirements, summarized here.

The overall goal of the Comet Interceptor mission is to provide the first investigation of an LPC, and to sample different regions of the coma simultaneously. The scientific requirements that need to be met to achieve this goal can be conveniently divided into the two Science Themes presented previously: First, measurement of the properties of the cometary nucleus that will allow the comparison of an LPC with short-period comet nuclei investigated by previous missions. Second, investigation of the coma, its connection to the nucleus (cometary activity) and its interaction with the solar wind, will take advantage of the multi-point perspective through three spacecraft.

The two science themes are first broken down into top level Science Objectives (Level 0) that describe the properties of the target to be investigated. Each Science Objective is further split in a series of Science Requirements (Level 1) that quantitatively describe the features, characteristics and processes that need to be measured to achieve the Science Objective. Finally, Level 2 Requirements, not included here for brevity, describe the measurements by each instrument that contribute to meeting the Level 1 requirements. Tables 1, 2, and 3 list the Science Objectives and the Level 1 Science Requirements.

**Table 1:**
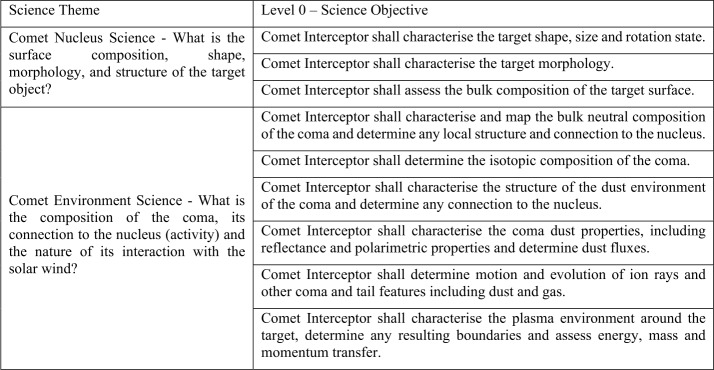
Science objectives of Comet Interceptor

**Table 2:**
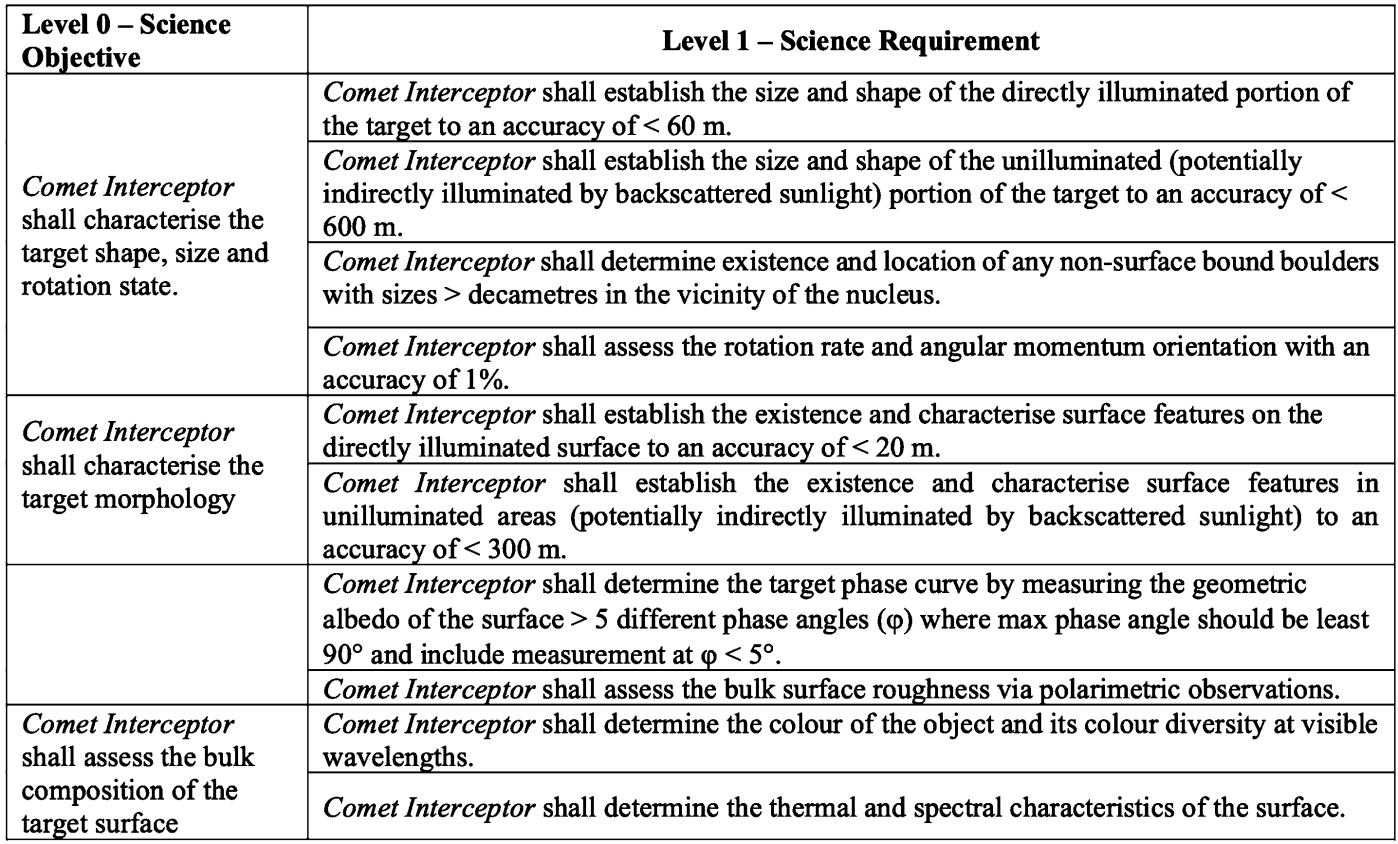
Top-level science requirements corresponding to the science objectives related to the cometary nucleus

**Table 3:**
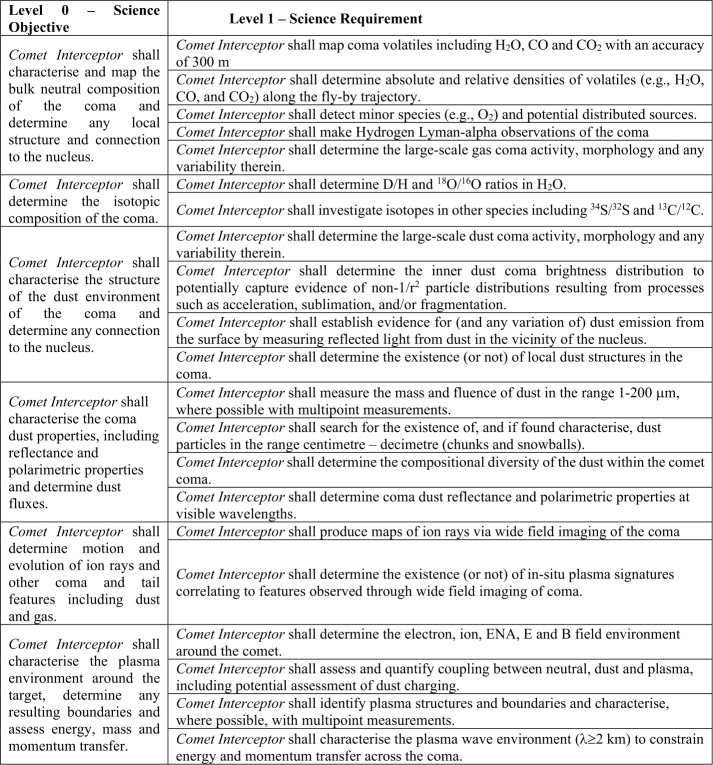
Top-level science requirements related to the cometary coma environment

## Planned Measurements and Payload Overview

The Comet Interceptor payload (Table 4) was selected to fulfil the mission Science Objectives.

**Table 4:** Summary of scientific instruments

Instrument	Sensor	Application
Spacecraft A		
CoCa		Visible-NIR hi-res imager
MIRMIS	NIR	NIR Hyperspectral imager
	MIR	MIR Point spectrometer
	TIRI	Multispectral thermal imager
MANiaC		Mass spectrometer
DFP	DISC	Dust detector
	FGM-A	Magnetometer
	COMPLIMENT	Electric field
	SCIENA	Ion and energetic neutral atom detector
	LEES	Electron spectrometer
Probe B1		
HI		Hydrogen imager
PS		Magnetometer
		Ion mass spectrometer
NAC/WAC	NAC	Narrow-angle imager
	WAC	Wide-angle imager
Probe B2		
EnVisS		All-sky imager
OPIC		Forward imager
DFP	FGM-B2	Magnetometer
	DISC	Dust detector

The Comet Camera (CoCa) on Spacecraft A will contribute to both mission themes by determining the physical properties of the nucleus and coma with high resolution images. CoCa will determine the physical properties of the nucleus with high resolution images, tracking the nucleus through the flyby. The current CoCa instrument design will

- Determine the size, shape, and albedo of the nucleus
- Identify features on the nucleus at <20 metres resolution (assuming a nominal 1000 km flyby) and allow comparison with similar observations of the surfaces of short-period comets
- Establish the reflectivity spectral gradient of the nucleus and of sub-regions on the nucleus (Thomas and Keller 1989; Fornasier et al. 2015)
- Search for evidence of surface ices for comparison with Rosetta and Deep Impact observations of SPCs (Sunshine et al. 2006; Pommerol et al. 2015)
- Constrain the rotation of the nucleus through, as a minimum, observation of dust coma structures
- Determine the spatial distribution of dust emission from the nucleus (including the dayside/nightside emission ratio, which may have implications for the presence of super-volatiles such as CO and CO_2_, Gerig et al. 2020)
- Investigate and constrain the properties of the acceleration region of the inner coma by studying dust column density profiles (Gerig et al. 2018; Zakharov et al. 2018)
- Establish the direction and relative magnitudes of jet-like structures for correlation with dust impact events on the spacecraft
- Provide pointing information including determination of the flyby geometry (Curdt et al. 1988)
- Identify impact events on the spacecraft by providing evidence of uncontrolled Spacecraft Attitude changes (Curdt and Keller 1990).

The infrared spectra taken by the Modular InfraRed Molecules and Ices Sensor (MIRMIS) will provide information about the composition and thermal properties. They will be supported by the Narrow Angle Camera (NAC) on probe B1 and the Optical Periscope Imager for Comet (OPIC) on probe B2, which will image the nucleus from perspectives different to those of Spacecraft A, providing stereo views and increasing the fraction of the surface that can be investigated. Probe B1 will also have a wide-angle visible camera (WAC) to image the nucleus and its surroundings from a fixed orientation during closest approach, and the OPIC instrument, on probe B2, will return resolved images of the nucleus and the inner coma from a different angle shortly before closest approach. Composition information will come from multi- colour imaging (four broadband filters in the visible range in CoCa; a tuneable hyperspectral imager in the 0.9 to 1.7 μm near-infrared region and fixed narrowband filters between 8.9 and 21.6 μm in MIRMIS) and point spectroscopy in the 2.5 to 5 μm range (MIRMIS/MIR). Unique insights into the physical properties of the surface layers (particle sizes, thermal inertia) will come from visible wavelength polarimetry measurements by the Entire Visible Sky instrument, EnVisS, on B2, and thermal-infrared temperature measurements (MIRMIS).

For the Comet Environment Theme, the coma composition will be measured *in situ* by the Mass Analyzer for Neutrals in a Coma (MANiaC), including measurement of isotopic ratios. Provided that the outgassing activity of the comet is sufficiently high, elemental, molecular, and isotope abundances of the gas will be derived by MANiaC. This includes monitoring the major coma volatiles H_2_O, CO, and CO_2_, key molecules such as O_2_, highly volatile species, and organic compounds, as well as hydrogen and oxygen isotopes in water. Even (icy) grains entering the instrument may be measured. MANiaC provides key measurements to study the comet’s activity and the material from which its nucleus formed and how it evolved. Gas composition measurements through remote sensing will be performed by MIRMIS. In addition, the Hydrogen Imager (HI) on probe B1 will monitor the cometary water production rate from months before the flyby until the end of operations. The spatial distribution of dust will be investigated from three different viewpoints by CoCa on Spacecraft A, the WAC on B1, and OPIC and the all-sky imager EnVisS on probe B2. EnVisS will additionally constrain dust properties through polarimetric measurements, and the Dust Impact Sensor and Counter (DISC), part of the Dust, Fields, and Plasma (DFP) instrument, will measure the mass distribution of dust particles colliding with both Spacecraft A and probe B2. DFP’s COMPLIMENT and LEES sensors will detect nanograin impacts.

The Dust Field and Plasma (DFP) package is a combined experiment dedicated to the multi-point *in situ* study of the multi-phased ionized and dusty environment in the comet’s coma, and its interaction with the surrounding space environment. DFP will measure the magnetic field, electric field, plasma parameters (density, temperature, and speed), the distribution functions of electrons, ions, and energetic neutrals, spacecraft potential, and cometary dust, in order to:

- Identify boundaries and regions in the cometary environment of a comet and its interaction with the solar wind (e.g., bow shock, diamagnetic cavity) and to assess their structure.
- Map the dust and plasma phases around the target.
- Assess mass, momentum, and energy transfer in the cometary environment.
- Provide simultaneous magnetic field, plasma, and dust measurements to identify the interplay between the ionized and dusty phases around a comet and characterize dusty plasma properties.
- Map the solar wind – coma interaction.
- Describe and map the (i) electron, (ii) negative and positive ion, and (iii) energetic neutral atom distribution functions in the vicinity of the comet and in the interaction region with the solar wind.
- Identify electron and ion kinetic processes that mediate the solar wind-comet interactions from ion kinetic scales down to electron scales.

DFP-SCIENA will measure energetic particles of solar wind and cometary origin, both with and without charge. The ion observational capabilities will allow the direct detection of solar wind and cometary ions, providing energy, direction, and estimated mass. These measurements are necessary to determine the 3D flow of plasma in the comet’s induced magnetosphere in order to assess the mass, momentum, and energy flow and transfer between different plasma regions, identification of plasma boundaries such as the bow shock, solar wind void and similar. The energetic neutral atom (ENA) measurement capability allows the study of the direct interaction of the solar wind with the neutral atmosphere, providing continuous monitoring of the solar wind’s dynamic pressure, an estimate of the position of the regions of strongest interaction between the solar wind and the coma as well as the coupling between the coma and the cometary ions.

Magnetometers are the only *in situ* instrument type present on all three spacecraft, though all three have different designs and instrument heritage. They will also detect various wave modes, such as mirror modes and ion cyclotron waves, to assess the energy transfer across these boundaries. Plasma properties will be derived from measurements of three spectrometers as part of DFP and an ion mass spectrometer as part of the B1 Plasma Suite (PS).

The following section provides more details of the mission’s scientific instruments.

## Scientific Instruments

### COmet CAmera (CoCa)

The camera system on-board Spacecraft A, CoCa, is required to provide detailed imaging of the nucleus and the innermost coma of the target. The design uses previous heritage to establish a baseline performance (Table 5) that surpasses that of previous flyby missions to comets. The instrument is based upon two elements. Firstly, it uses the telescope of the Colour and Stereo Surface Imaging System (CaSSIS) that is successfully operating at Mars on the European Space Agency’s ExoMars Trace Gas Orbiter (TGO) (Thomas et al. 2017). Secondly, the CoCa design uses the detector system of the JANUS instrument from ESA’s JUICE mission (Witasse 2021). By integrating these two elements, CoCa can achieve an angular scale of 8 μrad px^−1^, superior to cameras on all previous comet missions with the exception of Deep Impact’s HRI (A’Hearn et al. 2011). The scale is nearly a factor of three superior to that of the Halley Multicolour Camera, HMC, on-board Giotto (Keller et al. 1987). The detector system uses a rolling shutter technique to allow rapid image read-out with a minimum possible exposure time of 220 μs to avoid motion smear at closest approach for even the highest velocity flybys. A major difference here is that, unlike Giotto which was a spinning spacecraft, Comet Interceptor is a three-axis stabilised system implying that the exposure times can be selected. The detector allows saturation of the nucleus without blooming of charge. This, in turn, implies that the exposure times of selected images can be programmed to provide high signal to noise observations of the dust coma while saturating on the nucleus. This capability will increase the flexibility of the mission if targets are eventually found that have only weak dust emission.

**Table 5:** The main characteristics of the CoCa instrument

Parameter	Value
Instrument pixel scale	8 μrad/px
Field of View	0.69° × 0.92°
Detector	CIS115 Back-side illuminated
	CMOS image sensor
Pixels	1504 × 2000
Pixel size	7 × 7 μm
Exposure times	220 μs (flyby) to 15 min (identification), rolling shutter
Imaging rate multi-colour	≥1 frame s^−1^
Imaging rate single colour	≥2 frames s^−1^
Filters	475 nm (*Δλ* = 150 nm) BLU
	675 nm (*Δλ* = 100 nm) ORG
	775 nm (*Δλ* = 100 nm) RED
	900 nm (*Δλ* = 150 nm) NIR
Mass	13.5 kg (3 units)
Power	19 W average
Volume	CSU: 350 × 460 × 550 mm^3^
	PEU: 210 × 160 × 70 mm^3^
	ELU: 120 × 240 × 180 mm^3^
Data I/F	Spacewire
Instrument memory (holding science data)	2 × 128 Gbit
Max data volume	128 Gbit uncompressed

CoCa will be equipped with four selectable interference filters covering the sensitivity range of the detector (roughly 400 nm to 1000 nm). The filters will be around 150 nm in bandwidth and optimised to have approximately equal signal to noise ratio in all images of the nucleus. Low spectral resolution is not a disadvantage here as previous observations have shown that visible spectra are mostly featureless with a constant reflectivity gradient (e.g. Fornasier et al. 2015). Subtle broadband colour differences were seen by the Rosetta camera OSIRIS (Fornasier et al. 2016) requiring high signal to noise. A filter wheel mechanism has been designed to switch filters quickly to ensure minimum changes in phase angle between adjacent images. A goal of 1 second between images has been set and achieved in the prototype giving a change in phase angle for adjacent observations of ${<}1^{\circ}$. The passage through closest approach and imaging at high cadence will also provide a data set for establishing the 3D spatial distribution of dust in the inner coma using post-processing tomography techniques. The instrument is capable of acquiring around 2500 images during the encounter. Data compression (using a JPEG algorithm used extensively on CaSSIS) and sub-framing are foreseen to ensure that the on-board storage produces no practical limit to the flexibility of the data acquisition. It is intended that the instrument will be set into a “mode” by command and will continue imaging in this mode until there is a mode change or until CoCa is told to stop. CoCa therefore operates autonomously with pre-defined modes that can be programmed in flight.

**Fig. 16:**
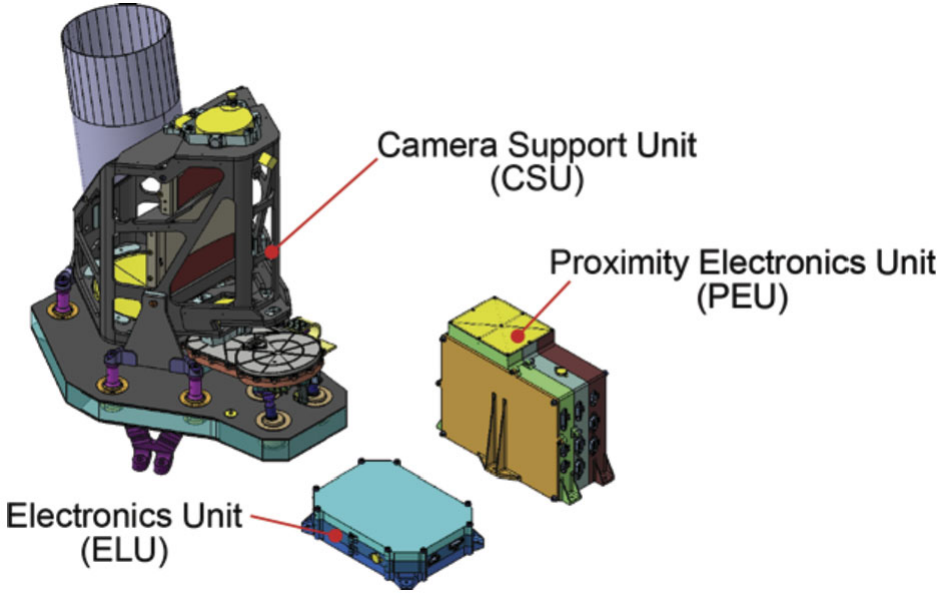
CAD/CAM of the CoCa instrument. Left: The Camera Support Unit (CSU). Light coming from the Rotating Mirror Assembly (RMA, Fig. 18) enters the instrument through the baffle (purple cylinder) and is reflected by the four mirrors of the telescope (yellow) onto the filter wheel assembly (Fig. 17) Right: The Electronics Unit (ELU). Centre: The Proximity Electronics Unit (PEU)

**Fig. 17:**
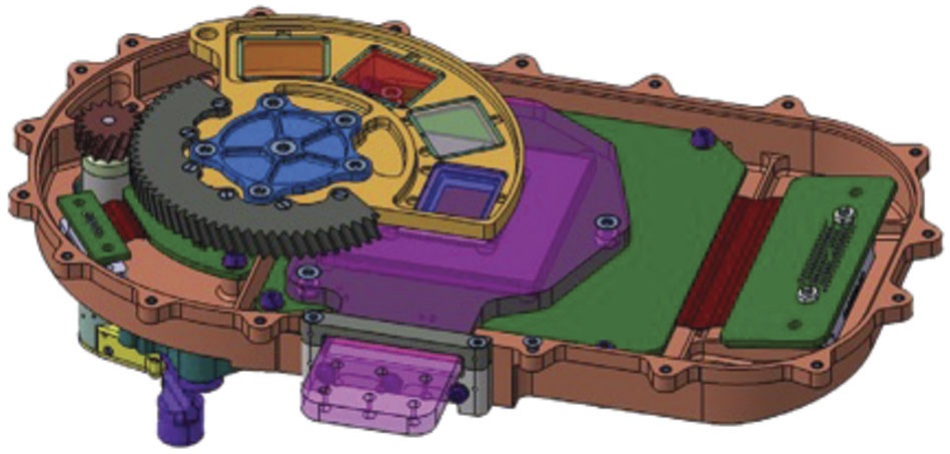
The CoCa filter wheel assembly with four filters. The detector is shown in pink below one of the filters. The filter wheel includes a launch lock to prevent motion during launch

The CoCa design is shown in Fig. 16. The Camera Support Unit (CSU) has numerous elements. The open structure in Fig. 16 is a 13.5 cm diameter 4-mirror off-axis telescope with a focal length of 880 mm following the design for the CaSSIS telescope on TGO. The structure is carbon-fibre reinforced plastic (CFRP). Following the CaSSIS experience, a small change to the internal baffling at the intermediate stop (between mirror M2 and M3) has been made and improved front baffle has been designed. Otherwise, the design is unchanged. The mirrors are silver coated, providing a field of view that is larger than the active area of the detector in the focal plane. The open structure ensures low mass and will finally be wrapped in multi-layer insulation to produce a light-tight unit. The telescope is mounted on a baseplate that also supports the detector and filter wheel assembly. The detector is a spare of the development for JANUS, the imaging system on JUICE. The sensor is a back-side illuminated (BSI) CMOS device from e2v with 1504 × 2000 pixels and 7 μm pitch, a peak quantum efficiency exceeding 90% (Crews et al. 2020) and a full-well of 27,000 e^−^. A radiator will be used to reduce the sensor temperature, with −30 °C being the goal, although nominal operation can be achieved at 0 °C. Combined with the telescope system, the detector provides a field of view of 0.69° × 0.92°. The filters (Fig. 17) will use fused silica substrates with standard interference coatings, designed for high throughput with sharp cut-offs and high out-of-band rejection. The PEU houses the proximity electronics for the detector while the ELU provides power conversion, instrument control, and data management.

**Fig. 18:**
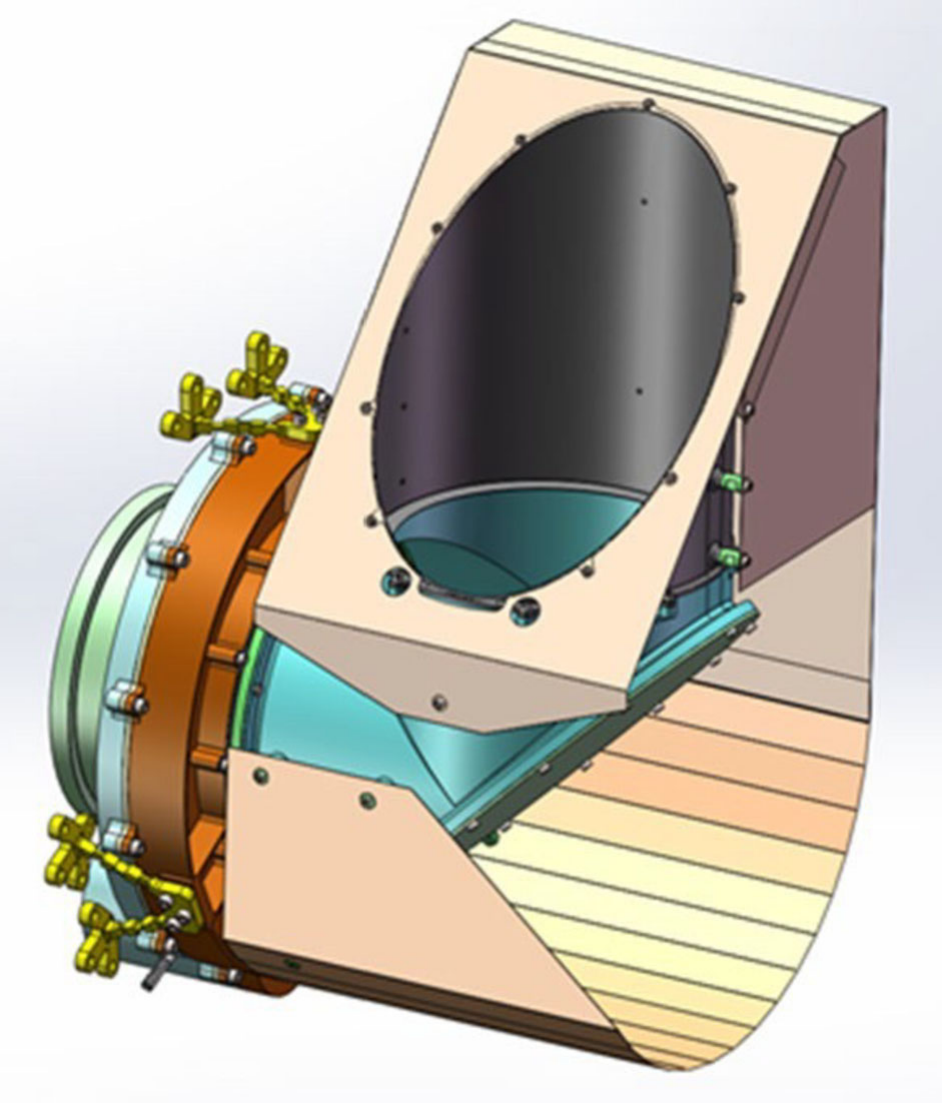
CAD/CAM drawing of the RMA showing the opening (top) and the fold mirror mount (turquoise colour) which rotates and reflects light toward CoCa (left). The mounting feet (yellow colour) attach the RMA to the exterior of the spacecraft while the entire CoCa instrument (Fig. 16) is inside with their optical axes aligned

Considerable effort has been invested in protecting CoCa from hyper-velocity dust impacts during the flyby. It is to be recalled that HMC was damaged severely during the 1P/Halley encounter despite being mostly behind the Whipple shield of the spacecraft (Schwehm 1991). In the case of Comet Interceptor, a rotating mirror assembly (RMA) has been developed which will allow CoCa to be mounted behind the protection shield while still providing a continuous view of the nucleus. The RMA has two elements (Fig. 18): the SMA (Scan Mirror Assembly), and the SME (Scan Mirror Electronics). The SMA is a mechanism holding the folding mirror and that will rotate this mirror in order to orient the field of view of CoCa towards the comet during encounter. It is based on a brushless DC motor moving the mirror via a gear system and an optical position sensor in order to allow closed loop control. The mechanism will be driven by the SME that will take care of powering the motor to position the folding mirror based on encounter parameters provided by the spacecraft platform combined with the read-out of the position sensor. The SMA includes a protection system that will hide the mirror from incoming dust particles during the most critical part of the encounter, when the spacecraft is closest to the nucleus.

CoCa is being built by an international consortium formed by the Instituto de Astrofísica de Andalucía (IAA Granada, Spain), the Research Center for Astronomy and Earth Science (CSFK, Budapest, Hungary), the Laboratoire d’Astrophysique de Marseille (LAM, France), and the DLR Institute for Planetary Research (Berlin, Germany) under the lead of the University of Bern (Switzerland). The University of Liége (Belgium) leads the development of the Rotating Mirror Assembly (RMA).

### Modular InfraRed Molecules and Ices Sensor (MIRMIS)

**Fig. 19:**
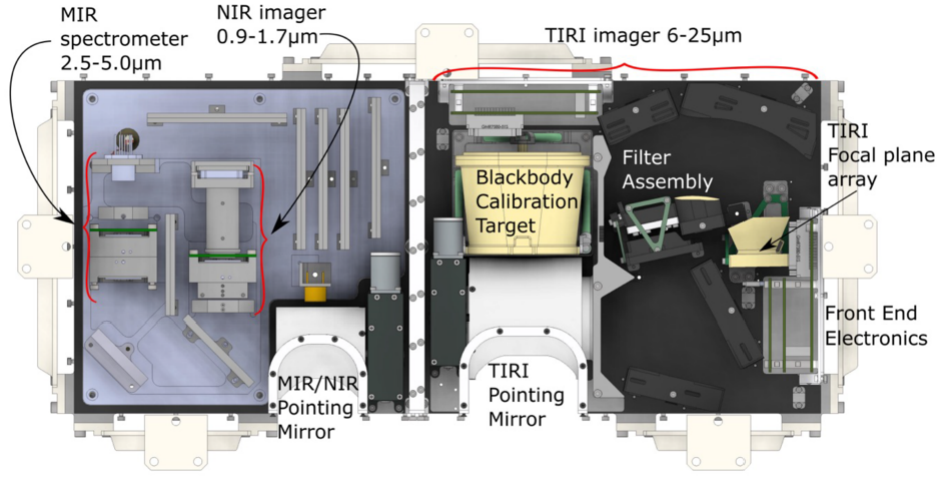
MIRMIS TIRI/MIR/NIR mounted on a common optical bench (548.5 × 282.0 × 126.8, in mm)

MIRMIS is the hyper and multi spectral remote sensing instrument for Comet Interceptor (Fig. 19, Table 6). The instrument covers a wavelength range of 0.9 to 25 μm which samples spectral features of CO_2_, H_2_O, CO, mineral compositions and is rich in thermophysical data. The instrument is made up of three closely integrated modules: the Near-IR (NIR)/Mid-IR (MIR) spectrometer and a thermal infrared imager (TIRI), with one thermal, mechanical, and electrical interface to the spacecraft. NIR is a 0.9 to 1.7 μm hyperspectral imager, MIR is a 2.5 to 5 μm point spectrometer and TIRI is a 6 to 25 μm multispectral thermal imager.

**Table 6:** MIRMIS instrument summary table

Scientific performance summary
Spectral range 0.9–25 μm using four channels:
NIR, hyperspectral imager, 0.9–1.7 μm
@20 nm spectral bandwidth
MIR, single-point spectrometer, 2.5–5.0 μm
@30 nm spectral bandwidth
TIRI, multispectral thermal imager, 6–25 μm

Key instrument numbers
TIRI: FoV = 9 × 7^∘^ (7 μm diffraction limit), iFoV = 0.26 mrad
NIR: FoV = 6.7 × 5.4^∘^, iFoV = 0.18 mrad
MIR: FoV = 2^∘^ circular
Total Mass (CBE): 8.8 kg with margin
Standby average: 8.3 W
Standby average with detector thermal control: 9.9 W
Average science operating mode (Nucleus pointing): 11.2 W
Average science operating mode (Coma monitoring): 9.7 W
Total module volume: 548.5 mm × 282.0 mm × 126.8 mm

The MIR and NIR modules are based on tunable Fabry-Pérot interferometers which are used as an adjustable bandpass filter (Näsilä and Kohout 2020). For the NIR channel the filter is combined with an InGaAs focal plane assembly to give a field of view (FoV) of ca. $6.7 \times 5.4^{\circ}$ from $640 \times 512$ pixels and spectral bandwidth of 20 nm. The NIR design is based on refractive optics with heritage from the ASPECT imager in the Milani CubeSat for ESA’s Hera mission (Kohout et al. 2018; Michel et al. 2022). A prototype version has been flying in low Earth orbit since November 2018 on the Reaktor Hello World nanosatellite. The basic operating principle of the MIR channel is the same as NIR but has a single HgCdTe detector with a ca. $2^{ \circ}$ FoV to provide point spectroscopy and spectral bandwidth of 30 nm,

The TIRI module is based on the Lunar Thermal Mapper instrument (Bowles et al. 2020) due for launch on NASA’s Lunar Trailblazer mission in 2023 with heritage from the Compact Modular Sounder (CMS) instrument flown in low Earth orbit between 2014–2017 on the UK TechDemoSat-1 spacecraft. TIRI uses gold-coated all-reflective optics with f/1.4 and a 50-mm aperture. A pointing mirror is used to direct the field of view onto the target object, a black body calibration target or a space view. Calibrations using the black body and space view will be performed immediately before and after each observation sequence. A two-mirror telescope directs the incoming infrared radiation onto a filter assembly used to define ten individual spectral channels (Shirley et al. 2022) for compositional and temperature mapping of the nucleus. This filter assembly is re-imaged onto a $640 \times 480$ uncooled microbolometer array using a three-mirror relay.

MIRMIS/NIR and MIRMIS/MIR are supplied by VTT Finland with the University of Oxford, UK providing MIRMIS/TIRI and overall integration of the instrument. The MIRMIS instrument team includes members from the UK, Finland, and the USA.

### Mass Analyzer for Neutrals in a Coma (MANiaC)

**Fig. 20:**
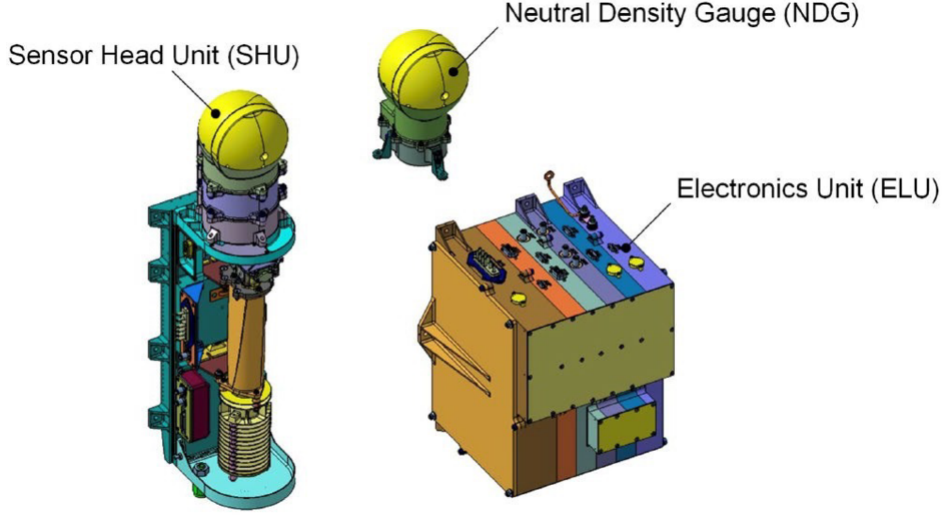
MANiaC consisting of a time-of-flight mass spectrometer (SHU, Sensor Head Unit), the Neutral Density Gauge (NDG), and the ELectronic Unit (ELU). For reference the long axis of the SHU corresponds to ∼470 mm. Only the antechamber spheres of both the NDG and the SHU (marked yellow) are exposed to the gas and dust flow of the coma and are covered by dedicated dust shields. The rest is enclosed and protected inside the spacecraft

MANiaC is dedicated to the *in situ* measurement of the neutral gas coma. MANiaC consists of two instruments, a time-of-flight mass spectrometer and a neutral density gauge (Fig. 20). The mass spectrometer obtains the relative abundances of the major and a subset of minor volatiles. The neutral density gauge measures the total gas density. Combining the measurements of both instruments yields the absolute densities of a suite of volatile species along the flyby trajectory.

Since the flyby velocity range of 10–70 km/s will be much larger than the neutral gas speed (∼1 km/s) MANiaC will be mounted on the spacecraft such that the aperture is always pointing in the direction of relative motion of the spacecraft. To cope with the large range of possible flyby velocities, both the SHU and the NDG contain antechambers for the thermalization of the incoming gas. Afterwards, the neutral gas entering the ion source will be ionized by impacting 70 eV electrons emitted by a hot filament. In the NDG, the resulting ions are measured as a current by a sensitive electrometer and in proportion to the gas density inside the antechamber and hence the surrounding coma. In the SHU, the newly formed ions are accelerated by a sharp extraction voltage pulse into the drift section. After passing the reflectron, i.e., an opposing electric field, the ions cross the drift section again before impinging on the Micro Channel Plate detector. Since the voltage pulse provides the same energy to all extracted ions, their arrival time on the detector can be converted into a mass/charge ratio. Combining NDG and SHU measurements leads to *in situ* abundances of coma gases.

MANiaC is being built by an international consortium formed by the Instituto de Astrofísica de Andalucía (IAA Granada, Spain), the Institut für Weltraumforschung (IWF Graz, Austria), the Institut de Recherche en Astrophysique et Planétologie (IRAP Toulouse, France), and Creotech Instruments S. A. (Piaseczno, Poland) under the lead of the University of Bern (Switzerland).

### Dust, Fields, and Plasma (DFP-A)

To enable multipoint *in situ* measurements of dust, plasma, and energetic atoms, five instrumental sensors, common data processing unit DAPU, and power supply system, PSU, will be constructed and placed on Spacecraft A, and two sensors with the respective DAPU and PSU will be present on probe B2 (see Sect. 5.10). Overall management of the DFP suite on both spacecraft A and B2 is by Centrum Badań Kosmicznych, CBK, Warsaw, Poland.

#### Dust Impact Sensor and Counter (DISC)

**Fig. 21:**
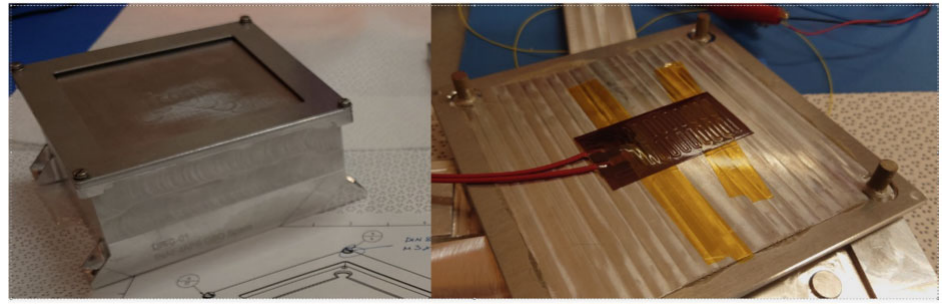
DISC Unit. Left: assembled DISC breadboard. Right: Sensing plate with glued PZTs at 3 corners

DFP-DISC will be provided in two twin units, mounted on spacecraft A and probe B2 (Fig. 21). DFP-DISC is devoted to the *in situ* characterization of cometary dust. In particular, DFP-DISC is designed to count the dust particles populating the coma and to determine the mass of each individual particle. The DFP-DISC design is a direct heritage of the Impact Sensor: one of the measurement sub-systems of the successful GIADA (Grain Impact Analyzer and Dust Accumulator) instrument. Full details of the instrument are provided in Della Corte et al. (2023).

DFP-DISC consists of:

- a square aluminum diaphragm, 0.5 mm thick with a sensitive area of $84 \times 84\text{ mm}^{2}$;
- three lead zirconate titanate ceramic piezoelectrics (PZTs), with a resonant frequency of 200 kHz, placed at three corners of the aluminum diaphragm;
- one PZT, placed at the fourth corner of the aluminum diaphragm, is the internal calibrator.

Each dust grain impacting the aluminum diaphragm generates acoustic waves that propagate in the diaphragm reaching the PZTs. These waves’ amplitudes are proportional to the momentum of the impacting dust particle. From the individual particle momentum measurement, knowing the relative speed between the spacecraft and the dust particles, DFP-DISC will determine the mass of individual impacting particles. DFP-DISC is a monitoring instrument with event driven acquisitions that will provide an *in situ* characterization of dust particles. It will count impacts and determine for individual grains their mass, impact duration, and density/structure. These measurements will allow the characterization of the dust coma structures along the spacecraft trajectory. The dust grain momentum measurement capabilities, combined with the foreseen flyby speeds, DFP-DISC will determine the dust mass distribution for grains in the range $10^{-15}\text{--}10^{-8}\text{ kg}$ (for particles with mass ${>}10^{-8}\text{ kg}$, the dust particle count will be provided).

DISC is being built by an Italian consortium scientifically led by INAF Osservatorio Astronomico di Capodimonte (INAF-OACN, Napoli, Italy) and having Leonardo S.p.A (Campi Bisenzio (Fi), Italy) as the Prime industry and the Italian Space Agency (ASI) as the main supporting funder. The contributing consortium partners are the University of Napoli “Parthenope” (Napoli, Italy) and the Istituto di Astrofisica e Planetologia Spaziali (INAF-IAPS Roma, Italy). DISC benefits also of the support of ESA, Univ. Parthenope and MUR (Ministero Università e Ricerca).

#### FGM-A

**Fig. 22:**
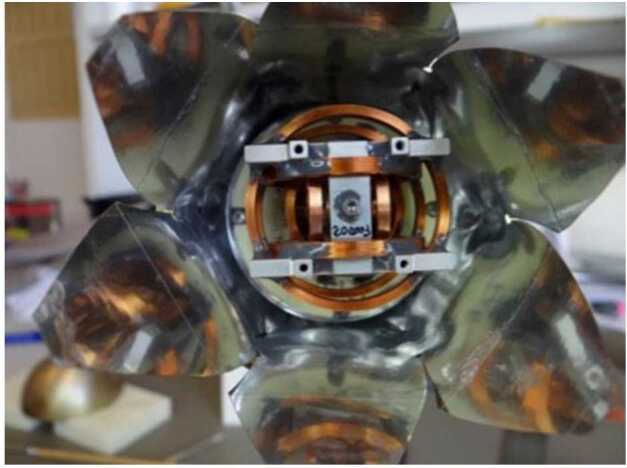
Integration of the FGM-A sensor within the COMPLIMENT merged probe

The FGM-A fluxgate magnetometer is composed of two sensors (outboard and inboard) mounted on a deployable boom (with heritage from Venus Express and Kompsat-2A) and their electronic front-ends, which are hosted in the DFP central electronics box, CEBOX. Its main properties are shown in Table 7. The fluxgate outboard sensor has been merged with the COMPLIMENT Spherical Probe, shown in Fig. 22. The combined sensor consists of a hollow spherical Langmuir probe that harbours a fluxgate magnetometer at its centre. Special precautions have been taken to minimize the possible interference between both whilst also being very lightweight. FGM-A is being built by an international consortium formed by the Technische Universität Braunschweig (Germany), the Institut für Weltraumforschung (IWF Graz, Austria) and Imperial College London (UK).

**Table 7:** FGM-A properties

Operation range	±16,000 nT (configurable)
Digital resolution	2 pT
Noise	<10 pT/sqrt(Hz) at 1 Hz
Absolute accuracy (requirement)	±1 nT (goal), ±2 nT
Mass	1.8 kg boom with sensors, 0.5 kg electronics
Power	1.2 W total
Temperature range	[−80; + 60] °C (both survival and operation). No heaters installed

#### COMPLIMENT

The COMetary Plasma Light InstruMENT unit (COMPLIMENT) is an electric field instrument that combines the capabilities of both a mutual impedance probe and a Langmuir probe. It is designed to probe the cometary plasma, the electric field and the (few nanometer-size) dust within the cometary environment. COMPLIMENT will provide the following measurements: electric field and waves (one component), high cadence independent ion and electron densities, electron temperature(s), spacecraft potential, integrated EUV flux, nanodust impacts (signal processed by DAPU) in order to address the structure and dynamics of the ionized and dusty cometary environment and its interactions with the escaping cometary atmosphere. COMPLIMENT (Table 8, Fig. 22) is composed of three sensors: two electric spherical probes (8 cm), one of which is a merged Electro-magnetic sensor (COMPLIMENT + FGM-A), mounted on booms and a transmitter, two electronic boards (LP and HMI) for signal generation, reception, and treatment, plus dedicated software hosted on the DAPU.

**Table 8:** COMPLIMENT properties

Electric field component *δ*E(t)	1 Hz–1.4 MHz
Electron density	10^2^–10^5^ cm^−3^, <1 Hz
Density fluctuation *δ*n/n	DC-1 kHz
Ion density $\mathrm{N}_{i}$	10^2^–10^5^ cm^−3^ , <1 Hz
Electron Temperature $\mathrm{T}_{e}$	0.01–30 eV , <1 Hz
spacecraft potential $\mathrm{U}_{sc}$	<100 Hz
Integrated solar flux	<1 Hz

DFP-COMPLIMENT is being built by an international consortium formed by the Belgian Institute for Space Aeronomy (BIRA-IASB, Brussels, Belgium), the Swedish Institute of Space physics (IRF, Sweden) under the lead of the Laboratoire de Physique et de Chimie de l’Environnement et de l’Espace (LPC2E, CNRS, Université d’Orléans, CNES, Orléans, France).

#### Solar Wind and Cometary Ions and Energetic Neutral Atoms (SCIENA)

**Fig. 23:**
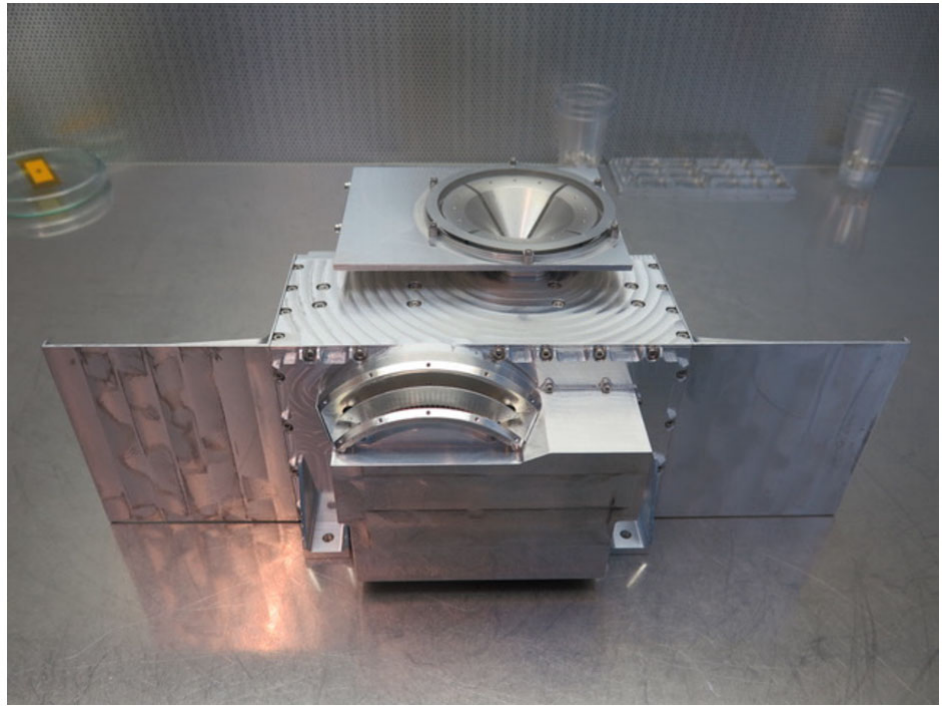
The SCIENA fully operational Technology Model, excluding some thermal hardware

SCIENA is an instrument of the SWIM family (Wieser and Barabash 2016) with one ion and one ENA sensor head (Fig. 23). The ion sensor head achieves a near 2$\pi $ steradian field of view through four directional electrodes, followed by a classical electrostatic analyser for energy determination in the range a few eV up to 15 keV (Table 9). Mass is determined through start and stop surfaces and a time-of-flight system. The ENA system is similar, but has only two direction electrodes and is performing a two-dimensional direction scan. In front of the direction electrodes are an ion rejector and a charge conversion surface, similar to what is used on the ASAN instrument operating on the far side of the moon (Wieser et al. 2020). The energy coverage is focussed on solar wind ENA at 300 eV to 3 keV. SCIENA is being built by the Swedish Institute of Space Physics (IRF, Sweden).

**Table 9:** SCIENA properties

	Ions	ENA
Energy range	10 eV–15 keV	300 eV–3 keV
Angular coverage	Near 2 *π*	30° × 150°
Mass resolution	1, 2, 4, 8, 16, 32 amu	1, heavy
Time resolution	1 s / energy spectra	1–10 s / energy spectra
	20–50 s / full distribution	5–50 s / full scan

#### Low-Energy Electron Spectrometer (LEES)

**Fig. 24:**
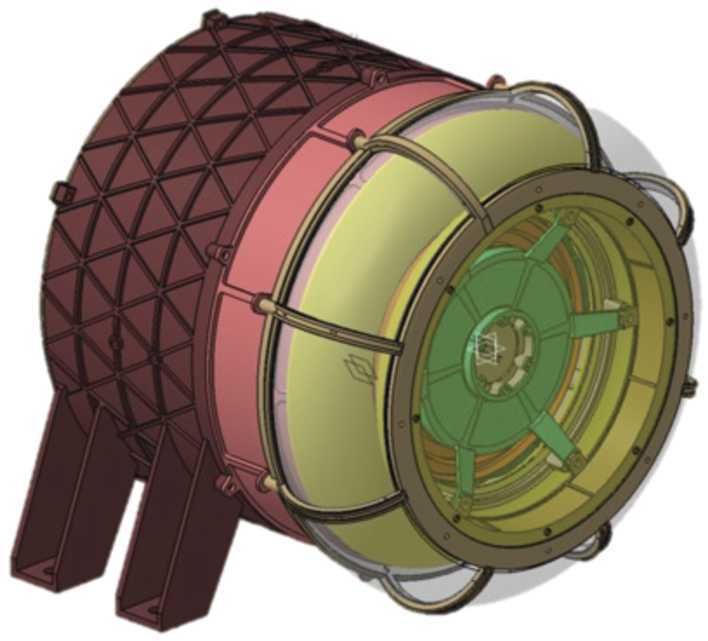
LEES instrument CAD model

LEES will determine the electron density, temperature, and the velocity distribution functions of the *in situ* plasma environment of the solar wind and comet (Fig. 24). LEES will detect the suprathermal photoelectrons created during neutral-plasma interactions in the coma and trace the magnetic connectivity between the spacecraft and the cometary environment. In addition LEES will measure the properties of negatively charged ions and dust of cometary origin. The energy range covered by LEES will be from a few eV up to 1 keV. Particles are measured in 360° azimuth angles with elevation angles ranging from −40° to 70° (Table 10).

**Table 10:** LEES measurement parameters

Ions	ENA
Energy range	10 eV–1 keV in 90 energy bins
Energy resolution	E/*Δ*E 0.07
Angular coverage	2.8 *π* sr
Time resolution	8 s / energy spectra

LEES is a classical top-hat type electrostatic analyzer with a Field-of-View (FoV) deflector system to allow the coverage of the elevation range by electrostatic deflection of incoming electrons. Incident charged particles enter the sensor through the exterior electrically grounded toroidal aperture grid. The particles are steered from the arrival direction into the top-hat electrostatic analyzer (ESA) using voltages applied to the upper deflection plate. The ESA section permits only electrons of the selected energy to reach the detector subsystem with MCPs (microchannel plates) in chevron stack. The MCP anode is divided in 16 sections that provide 16 azimuth bins with about 20° of angular resolution. Such a design allows fulfilling all scientific requirements for the electron and negative ions measurements.

LEES is being developed by the Institut de Recherche en Astrophysique et Planétologie in France, with significant contributions from Charles University in the Czech Republic and the Laboratoire d’Astrophysique de Bordeaux in France.

#### DAPU-A

The DAPU is a central data processing unit of the DFP instrument suite both on Spacecraft A and B2. It is a computer board serving as a common digital interface between the spacecraft and the DFP instruments and sensors. DAPU will:

- Perform last stage processing, compression and buffering of all science data,
- Store the data from the entire flyby in its large flash memory (as a backup copy in case),
- Manage common DFP suite modes, instrument commanding and configuration.
- DAPU-A will also count dust particle impacts in COMPLIMENT probe voltage data, by sensing the plasma clouds from the vapourised dust grains. This will allow the detection of small dust particles down to <100 nm across.

Development of the DAPU units for spacecraft A and probe B2 is led by the Institute of Atmospheric Physics (IAP) of the Czech Academy of Sciences, Prague, Czechia.

#### PSU-A

The Power Supply Unit (Table 11) shall generate, condition, control, monitor, and distribute electrical power to the DFP units from two unregulated 28 V buses, to fulfil the instrument power demands throughout all mission phases. The instrument power interfaces toward the spacecraft have been designed to prevent any single point failure, which could lead to a short circuit. The PSU modules include current and voltage monitoring, soft-start circuits, over-current protection (OCP), over-voltage protection (OVP), and under-voltage lock-out circuitry (UVLO) for the protection of the DFP units and subsystems. The current design of the PSU is based on previous and ongoing instrument power supply designs that have been implementing isolated DC/DC converters with embedded logic for switching control, protection, and HK monitoring. The design has heritage from instruments also built at Centrum Badań Kosmicznych, CBK, that were mainly used for radio and plasma diagnostics, i.e. Chronograph Control Block for PROBA-3 (ESA) and RELEC (Russia).

**Table 11:** The parameters of the PSU-A unit

Parameter	
Supply Voltage (unregulated) [V]	24–34
Power [W]	22
Redundancy	YES (NOM/RED)
Mass [kg]	0.5 (NOM+RED)
Secondary output voltages	3.7 V, ±5 V, ±12 V

### Hydrogen Imager (HI)

**Fig. 25:**
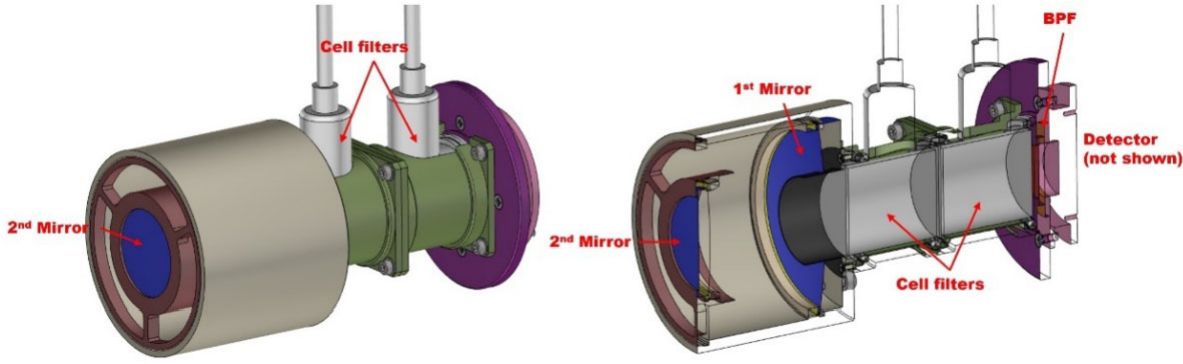
Design overview of HI

HI is an ultraviolet imager for hydrogen Lyman-$\alpha $ emission with parameters as listed in Table 12. The instrument aims to characterize the spatial distribution of the hydrogen coma through the imaging observation during the approach to the target. From the radial profile of hydrogen around the nucleus, the water production rate and its spatial variation will be deduced. HI is a Cassegrain-type telescope, with mirrors coated with Al/MgF_2_. A bandpass filter is installed on the light axis (Fig. 25). The photon detection system consists of MCPs combined with a Resistive Anode Encoder (RAE), enabling two-dimensional imaging. The electron cloud generated by the MCPs is divided into the four corners of the RAE, and the arrival position of photons is determined based on the relative charge distribution. HI offers two observation modes: the “light curve mode” and the “imaging mode”. In the former, a detector counts the number of incident photons per frame, while in the latter, the main processor calculates the position of each photon and integrates them into a 256 × 256 matrix over a fixed time span. Two additional gas filters are installed along the light axis. The narrow bandpass filters contain atomic hydrogen and deuterium individually when activated by filaments. By using these filters, the brightness ratio of the isotopic components for the resonant absorption of Lyman-$\alpha $ radiation can be determined, which occurs at 121.567 nm for hydrogen and 121.534 nm for deuterium. However, it is important to note that the measurement accuracy achieved using the gas filters strongly depends on the geometric conditions of the target and the B1 probe, which can cause a Doppler shift in the line centre. The HI consortium is led by the University of Tokyo, with contributions from Rikkyo University and JAXA.

**Table 12:** HI parameters

Parameter	
Power	<9 W (nominal), 12 W(max.)
Mass	<1.6 kg
Field of View	±2.1^∘^
Spatial resolution	0.02^∘^
Bandpass filter	120 nm, FWHM 10 nm
Hydrogen filter	121.567 nm (FWHM ∼3 pm, depending on filament temperature)
Deuterium filter	121.533 nm (FWHM ∼3 pm, depending on filament temperature)

### Plasma Suite (PS)

**Fig. 26:**
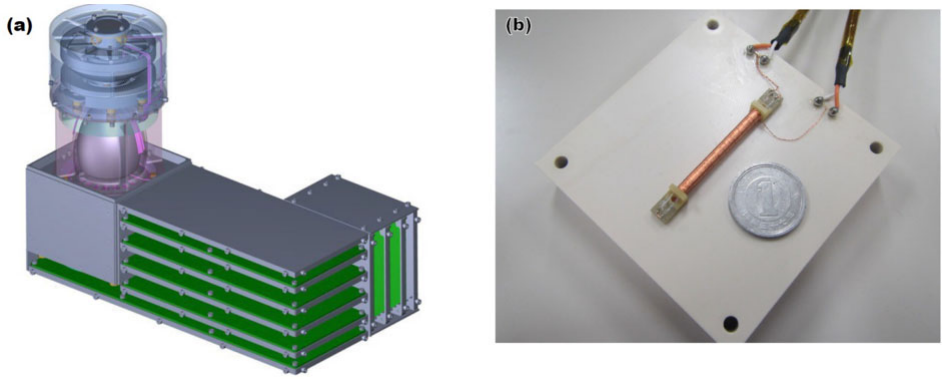
(a) PS structure including electronics boxes and (b) a magnetometer breadboard model

PS consists of an ion mass spectrometer and a 3-axis magnetometer (Fig. 26). It provides velocity distribution functions of individual ion species of the low-energy coma plasma, as well as the DC and low-frequency AC magnetic field data. The ion sensor consists of an electrostatic analyser and time-of-flight mass analyser (TOF). The incident energy and direction of each incoming ion are determined by the electrostatic analyser. Ions are then introduced to the TOF sector, where mass-per-charge is measured by the linearly increasing electric field. A large field-of-view (entire hemisphere if there is no exterior interference) is achieved by an entrance deflector in the electrostatic analyser unit. The PS magnetometer is based on the fundamental mode orthogonal fluxgate (FM-OFG) technique. FM-OFG adopts an amorphous wire sensor core driven with a unique excitation method where AC current is superposed on DC bias current. It enables low-noise detection of magnetic field with a compact and lightweight sensor design. The magnetometer is accommodated on the top of an extensible boom for magnetic cleanliness of the measurements. The boom is stowed during the launch and deployed during commissioning. The key performance aspects of PS are summarised in Table 13.

**Table 13:** Expected performance of PS

Ion mass spectrometer:
Instantaneous 3-D ion distribution with mass discrimination
Energy: 10–20,000 eV/q, *Δ*E/E ∼10%
Mass: M/*Δ*M ∼30
Field-of-view: Hemispheric
Magnetometer:
DC and low-frequency AC magnetic field
Absolute accuracy:
1 nT @ ±512 nT Range
Directional accuracy: <5 degrees
Noise level:
∼12 pT/Hz^1/2^ @1 Hz
∼6 pT/Hz^1/2^ @10 Hz

The PS consortium is led by the University of Tokyo, with contributions from Kyoto University, Osaka University, Kobe University, and JAXA.

### Narrow Angle Camera (NAC) and Wide Angle Camera (WAC)

**Fig. 27:**
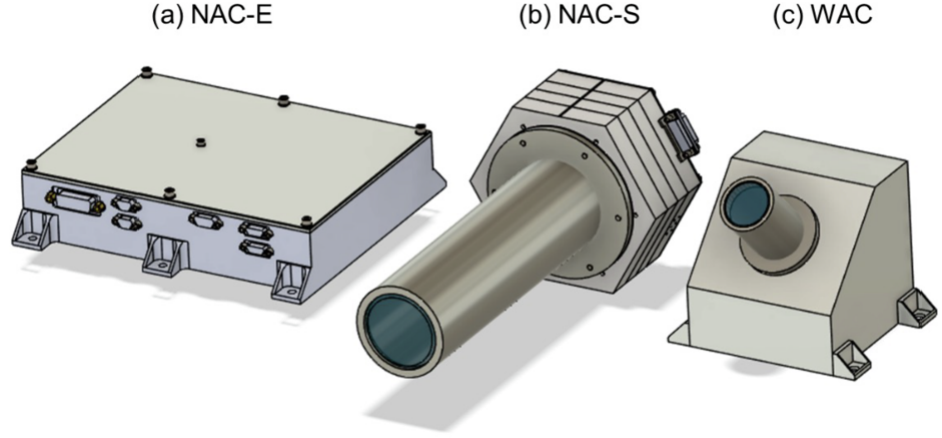
Components of the NAC/WAC system

The Narrow Angle Camera (NAC) is an optical telescopic camera. NAC will obtain optical images of the target nucleus with a high solar phase angle, to address nucleus science. The field of view is $3.5 \times 2.6^{\circ}$, which is anticipated to be wide enough to observe the entire nucleus. NAC is equipped with a CCD sensor of $3296 \times 2472$ pixels. The pixel resolution of the NAC is about 15.6 m/pix or better at a closest approach distance of 850 km. The Wide Angle Camera (WAC) is also an optical camera with CMOS imaging sensor of $2048 \times 2048$ pixels, which observes the coma with wide-field of view of $90 \times 90^{\circ}$. The instantaneous field of view is roughly 40 times wider than that of the NAC. Both cameras have panchromatic filters covering 0.4 to 0.75 μm in wavelength. They are utilized not only for the scientific objectives, but also for the optical navigation of the B1 probe. The NAC/WAC system (Fig. 27) consists of: (a) electronics box with control function of the both cameras and interface to the bus system (NAC-E), (b) Narrow angle camera sensor and optics (NAC-S), and (c) Wide angle camera sensor and optics (WAC). NAC-E and NAC-S will be based on the telescopic camera TENGOO on-board the Martian Moon Explorer – MMX – mission (Kameda et al. 2021).

The NAC and WAC consortium is led by the Institute of Space and Astronautical Science (ISAS), Japan Aerospace Exploration Agency (JAXA), with contributions from Rikkyo University.

### Entire Visible Sky (EnVisS)

**Fig. 28:**
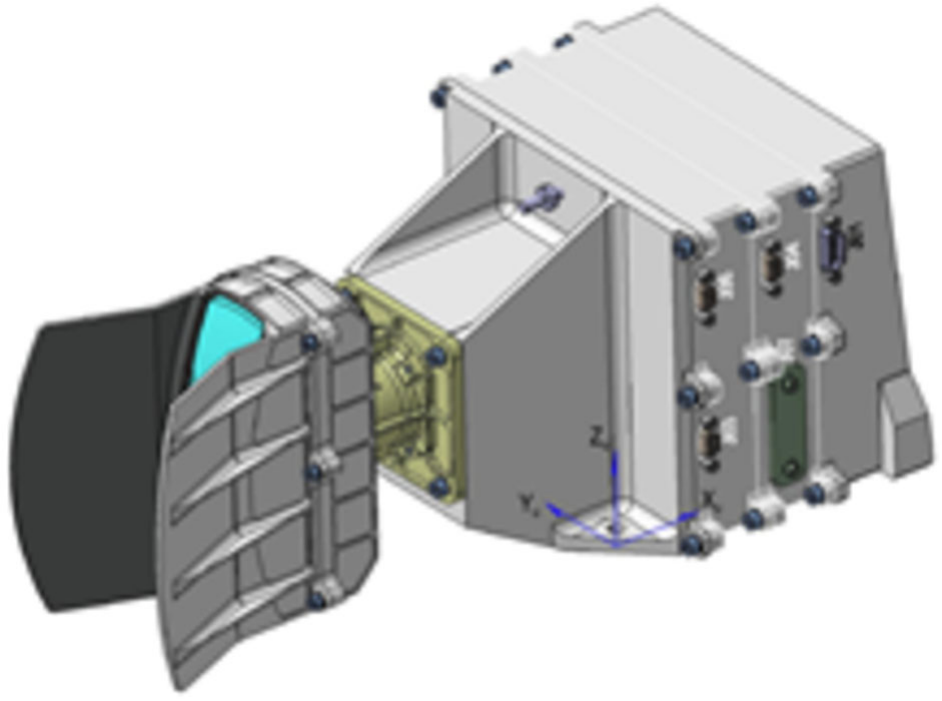
EnVisS instrument present mechanical layout (Courtesy of Leonardo SpA-IT)

The EnVisS camera (Fig. 28) has been conceived to study the comet’s dust environment in the visible range, at wavelengths of 550–800 nm. The intensity, degree of linear polarisation, and polarisation angle orientation of the light scattered by the dust particles in the comet coma, with a full 180° phase angle coverage, will be studied. Such a measurement is unique, it has never been carried out in space. Giotto’s Halley Opical Probe – HOPE – could only observe a very narrow angle FoV in the direction opposite to the motion of the spacecraft (Levasseur-Regourd et al. 1986).

The FoV of the instrument is designed to allow the entire sky to be acquired thanks to the rotation of the B2 spacecraft. EnVisS will feature a flexible push-broom/push-frame imaging technique, thus acquiring slices of the sky, while the probe rotates; the slices acquired will be stitched together, on-ground, to form a whole sky image. The coma investigation will be conducted throughout the full flyby of the comet from an advantageous point inside the coma itself. The probe spin-axis will be pointed at the comet nucleus for most of the time, except at closest approach when the comet nucleus will fall inside the camera FoV.

Depending on the target object activity, the map of the coma will be taken with different spatial resolution (i.e., smearing through adjustment of exposure length, and pixel binning) to achieve the desired SNR. The minimum angular resolution element corresponds to 0.2°. EnVisS features a fish-eye lens coupled to a commercial space-qualified detector and ad-hoc power and data handing units and software. The design solution adopted for the filters, i.e., fixed filter strips mounted as near as possible to the detector, allows for a compact, low mass and low complexity camera. Three broad band filters, all working in the same wavelength range, are foreseen for the camera: one non polarizing filter, centred on the detector, and, on either side, two polarimetric filters with transmission axis angles oriented at 45° one to the other. The full EnVisS characteristics are summarized in Table 14.

**Table 14:** Summary of EnVisS characteristics

Parameter	
Wavelength coverage	550–800 nm
	1 broad band filter
	2 polarimetric filters
Instrument FoV	180^∘^ × 45^∘^ (fixed)
	180^∘^ × 360^∘^ (dynamic)
Entrance aperture (F#)	1.23 mm (2.8)
Detector	CMOS 2k × 2k
	5.5-μm px size
Scale factor	0.1°/px
MTF	>70% @ 45 lp/mm
Distortion & telecentricity	<8% (f-theta distortion law) and <4° (at the FoV edges)

A flexible approach has been devised to allow a SNR of 10 to be obtained in the case of broadband images and of 100 for the polarimetric images. The signal from the coma in the direction of the apparent motion of the scene is not expected to show big changes, so a high spatial resolution is not needed from a scientific point of view. The integration time for each filter strip can be tuned, allowing for some smearing in the along-track direction. The spatial resolution can be retained in the across-track direction to assure a sampling of the comet phase function every 0.2°. This strategy will also allow for an adjustment of the exposure time if the radiance of the coma is different to the expected. Should the signal be extremely low, further pixel binning on-board, or co-adding, on-ground, of the images over different rotations, could be considered.

EnVisS is being realized by an international consortium scientifically led by Istituto di Fotonica e Nanotecnologie – Consiglio Nazionale delle Ricerche (CNR-IFN, Padova, Italy) and having Leonardo S.p.A (Campi Bisenzio (FI), Italy) as Prime industy. Instituto de Astrofísica de Andalucía (IAA-CSIC, Granada, Spain) is co-leading and, with SENER (Barcelona, Spain), is responsible for the DHU and PHU development. The contributing consortium partners are: Istituto Nazionale di Astrofisica (INAF-OAC Napoli, Italy), responsible for the instrument management, with the support of University of Napoli Parthenope (Napoli, Italy); and Huld (Espoo, Finland), with the support of Aalto University (Espoo, Finland), responsible for the ASW development. Preliminary instrument conceptual design was carried out by the University College London’s Mullard Space Science Laboratory, MSSL, UK.

### Optical Periscope Imager for Comets (OPIC)

**Fig. 29:**
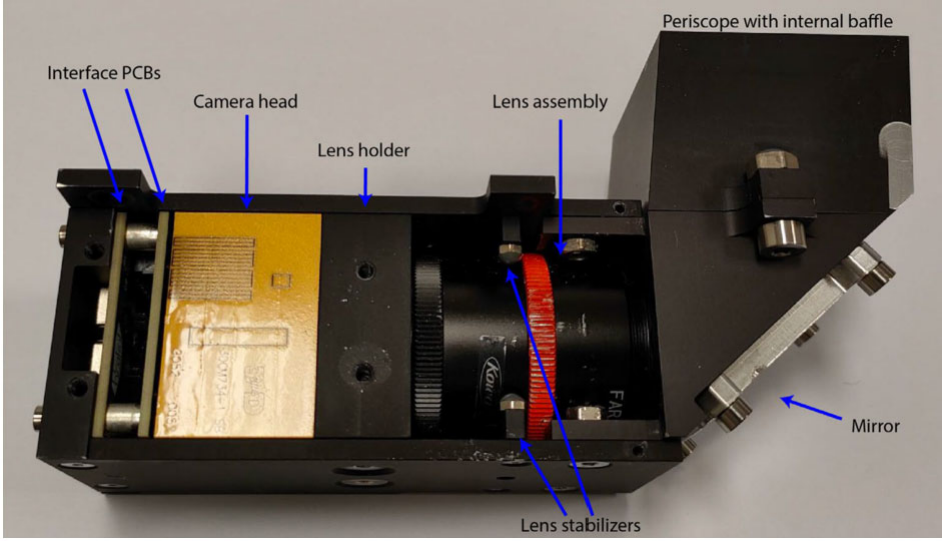
OPIC engineering model with internals exposed

OPIC, situated on probe B2 and looking over the edge of its dust shield, is an automated camera system for taking images of the target and its near environment (Fig. 29). It consists of an automated camera head (3D Plus 3DCM734-1 SS), imaging optics (lens assembly and baffled periscope) and interface electronics. It has a $2048 \times 2048$-pixel CMV4000 sensor and an integrated ProAsic3 FPGA. OPIC’s field-of-view is ${\sim} 18.2 \times 18.2^{\circ}$. OPIC is connected to the EnVisS instrument for additional data handling and power.

When far from the nucleus, OPIC will take long exposure images of the area around the nucleus, when the nucleus is not resolvable. These images will show the amount and spatial distribution of gas and dust is in OPIC’s viewing direction. The data can be also used to constrain the trajectory and rotation state of B2 after separation.

When the nucleus becomes resolvable, the captured images will be processed within OPIC before transmission to Spacecraft A. The images will show the low-resolution structure of the nucleus and of the gas and dust immediately around it, including potential satellites and fragments. This can be combined with imagery from the A and B1 platforms to generate a more detailed and less ambiguous 3D model of the target. OPIC is being built by Tartu Observatory, University of Tartu, Tõravere, Estonia.

### Dust, Fields, and Plasma (DFP-B2)

The DFP sensors on probe B2 will measure the magnetic field and the cometary dust. Two instrumental sensors, DISC, and FGM-B2, a slightly modified common data processing unit DAPU, and power supply system PSU will be constructed and placed on probe B2. The Power Supply Unit on B2 is similar to PSU-A, but with the parameters listed in Table 15. The Dust Impact Sensor and Counter (DISC) on Probe B2 is identical to that on Spacecraft A (see Sect. 5.4).

**Table 15:** The parameters of the PSU-B2 unit

Parameter	
Supply Voltage (unregulated) [V]	28 V
Power [W]	10
Redundancy	No
Mass [kg]	0.25
Secondary output voltages	3.4 V, ±5 V

#### FGM-B2

**Fig. 30:**
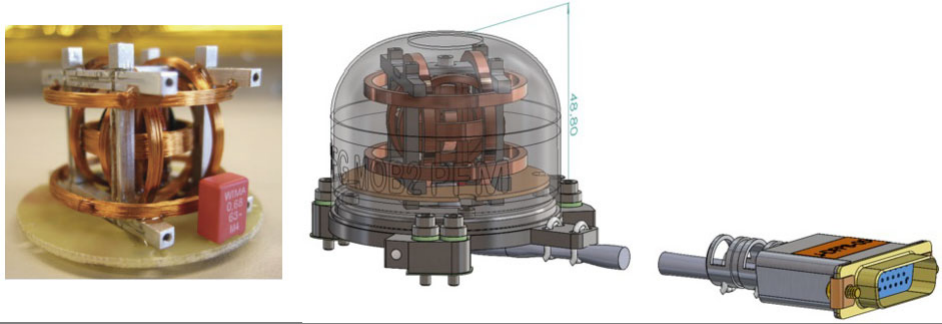
Picture of its sensing elements (left) and CAD rendering (right) of one FGM-B2 sensor

FGM-B2, or BFG for short, (Fig. 30) is composed of two sensors (outboard and inboard) mounted on a rigid boom, and their electronic front-ends, which are hosted in the DFP CEBOX. Whilst both sensors and electronics have a strong heritage (e.g., Rosetta/Philae, Venus Express, and THEMIS (sensors) and MMS, Geo-KOMPSAT-2A/SOSMAG (electronics)), their mass and power consumption have been optimised for the resource-constrained Probe B2. The instrument has a dynamic range of ±1000 nT (compensation range ±9000 nT), a digital resolution of 31 pT and an accuracy per component of at least ±2 nT. The noise level is <10 pT/sqrt(Hz) at 1 Hz.

FGM-B2 is being built by an international consortium formed by Imperial College London (UK), the Institut für Weltraumforschung (IWF Graz, Austria) and the Technische Universität Braunschweig (Germany).

## Mission Design

### Mission Analysis

To recap, the Comet Interceptor mission aims to intercept an LPC, ideally a DNC that is approaching the inner Solar System for the first time, or even an interstellar body. The encounter involves a close-approach flyby scenario using three elements: a mother spacecraft, spacecraft A, and probes B1 and B2 carried as payloads until the flyby and delivered to different flyby trajectories. This will allow the gathering of multi-point observations of the comet and its coma.

LPCs are typically discovered as they reach the inner Solar System, no more than a few years before perihelion passage. Their orbits are therefore not known in advance. An innovative and flexible mission concept is therefore required. By waiting in an orbit around Sun-Earth Lagrange Point 2, SEL2, typically for up to 3 years before being targeted, the probability of finding a suitable LPC that can be reached in time is considerably increased. The likelihood of a suitable LPC being found during the waiting period will be greatly increased soon by the availability of the LSST, with which it is expected that during its routine operations, much earlier LPC discoveries, at heliocentric distances ∼20 au will be achieved, giving warning times of >5 years before targeting. This may be early enough to know the target comet before launch, although not before the mission and spacecraft designs have to be frozen.

Comet Interceptor will transfer using its on-board propulsion system to a single flyby of the target during which all science measurements will occur (Sánchez et al. 2021). Science data from probes B1 and B2 will be transmitted to spacecraft A and stored on-board. The downlink to Earth of the science data will take place in the months immediately following the comet flyby. In the unlikely event that no suitable target is identified, Comet Interceptor will transfer to a backup target from a list of known short-period comets. A request for community observations to characterise the preliminary set of mission backup targets was published by Schwamb et al. (2020). A request for observations of the revised backup targets is to be published in the near future.

#### Target Comet Population

**Fig. 31:**
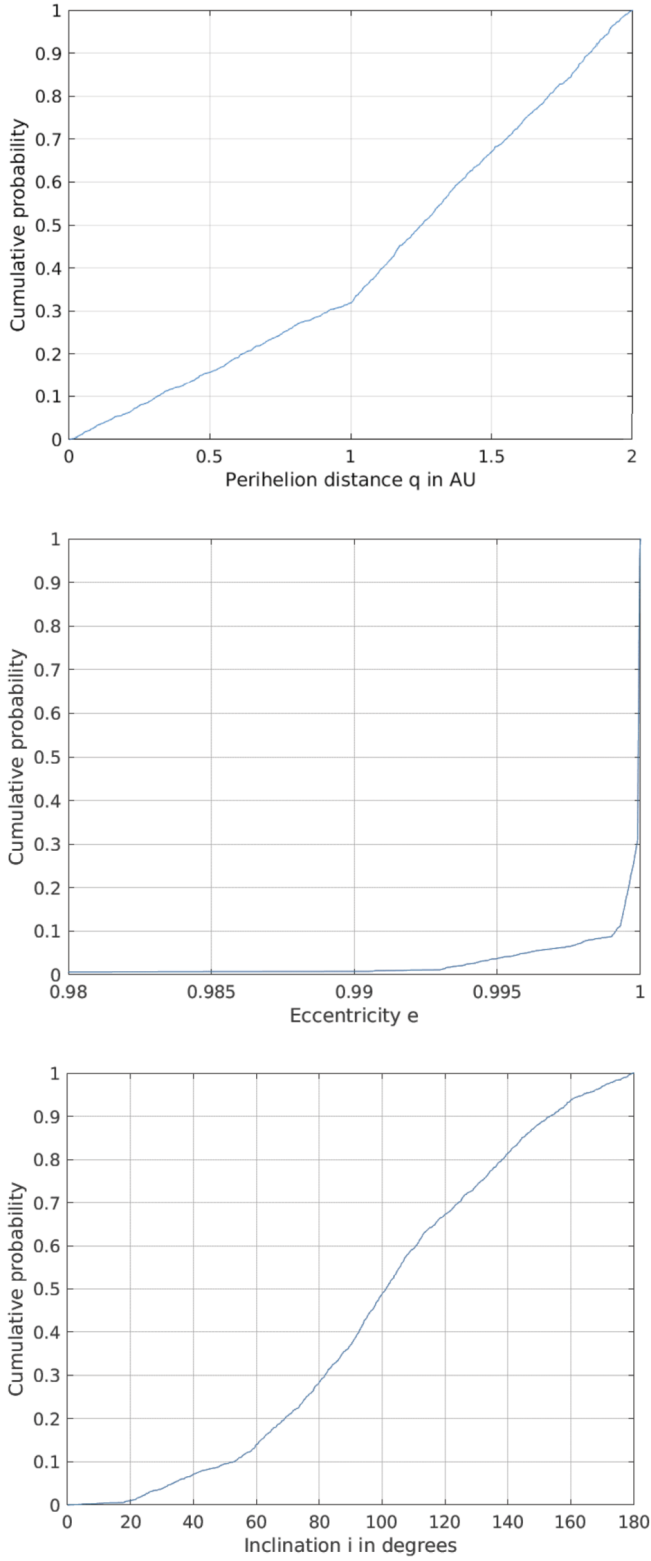
Empirical CDFs of LPCs with perihelion <2 au

The characterization of the LPC population is based on numerical studies of the evolution of these objects’ orbits and comparisons with observational data, in particular from the Pan-STARRS1 survey (Wiegert and Tremaine 1999; Boe et al. 2019). The Comet Interceptor Science Consortium provided a set of 1699 LPCs with perihelion occurring inside 2 au, which has been used to derive statistical distributions for the orbit parameters of the LPC population. Figure 31 shows the cumulative distribution functions (CDF) for the perihelion distance, eccentricity and inclination. LPC orbits are quasi-parabolic with a probability of eccentricity peaking steeply very close to 1 (the minimum eccentricity in the set is 0.9504), and more likely to be retrograde (64% probability of inclination >90^∘^). The other orbital elements follow an essentially uniform distribution. Correlations between orbital elements are neglected for the simulations carried out in the context of Comet Interceptor.

#### Launch, Transfer to SEL2, and Waiting Phase

Comet Interceptor is scheduled to launch in late 2029 with the ESA mission ARIEL on an Ariane 62 launcher. The launch configuration envisages the use of the Ariane 6 short fairing (TBC) and the Dual Launch Structure, with Comet Interceptor as the upper passenger and ARIEL as the lower. The injection orbit for Comet Interceptor will be a $9^{\circ}$ inclined high-apogee, nearly parabolic orbit with perigee at 180 km altitude and apogee at 1.5 million km. The Ariane 62 will use a direct ascent strategy, with a single boost of the upper stage’s Vinci engine. An additional biasing boost between both spacecraft separations will be implemented to reach slightly different SEL2 transfer trajectories, unbiased for Comet Interceptor and biased for ARIEL. This launch scenario results in a total wet launch mass for Comet Interceptor limited to ∼975 kg, excluding the mass of any required launch adapter.

A large amplitude quasi-halo orbit is selected on the basis of the following arguments:

- Compatibility with ARIEL also targeting a large amplitude SEL2 orbit.
- Minimization of the $\varDelta $V required for the transfer and no need for an insertion manoeuvre.
- Mitigation of eclipses during transfer and in the orbit around SEL2.

**Fig. 32:**
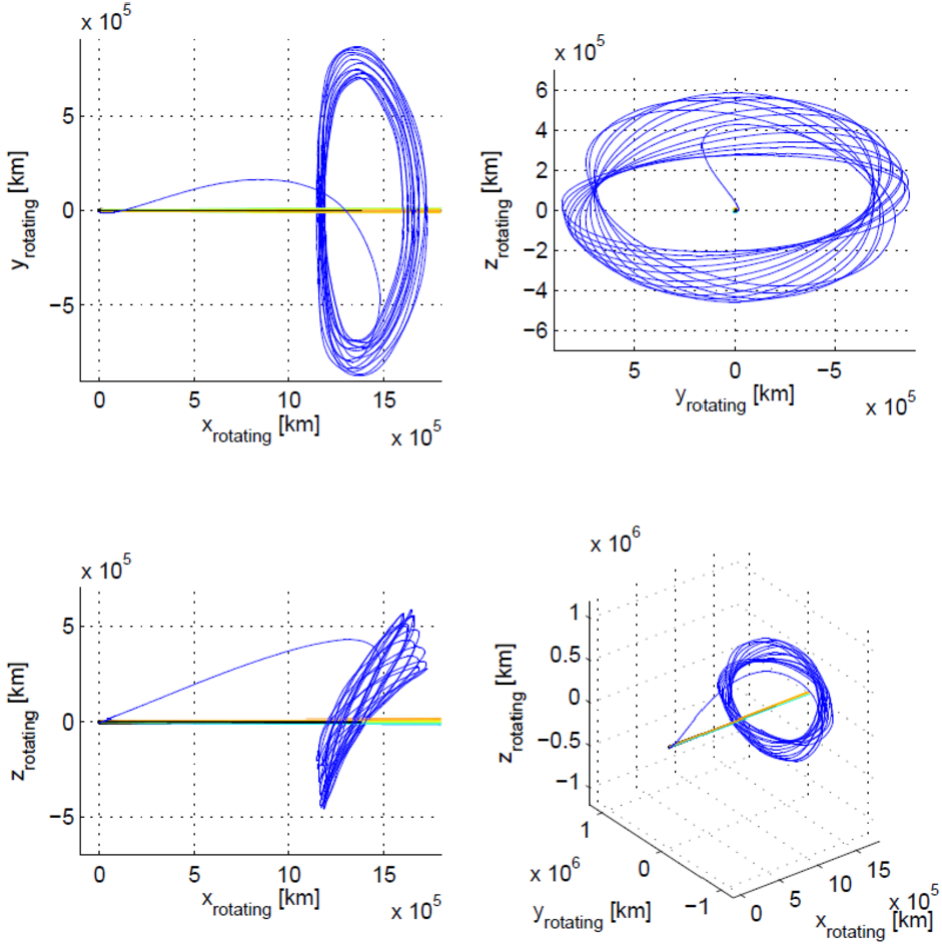
Sample transfer to SEL2 quasi-Halo orbit and waiting phase in the Sun-Earth rotating frame

A sample direct transfer to a large SEL2 quasi-halo and waiting phase around SEL2 are depicted in Fig. 32, showing possible orbit features and geometry.

The transfer geometry and amplitude of the achievable quasi-halo orbit will depend on the launch date and time. Assuming that a single Ariane 62 flight program is used, the natural variation of the perigee velocity over the launch window will have to be corrected with a manoeuvre 2 days into the mission. This manoeuvre will be combined with the correction of launcher injection errors. More trajectory correction manoeuvres are planned at days 5 and 20 to achieve an accurate manoeuvre-free transfer into the quasi-Halo orbit. Including the deterministic and the stochastic parts, 50 m/s are allocated overall, for the trajectory corrections during this part of the mission. The overall transfer duration is ∼3–4 months, during which commissioning activities for the spacecraft and probes will be performed.

Comet Interceptor will orbit SEL2 for a currently-unknown duration, likely between a few months and 4 years. It is during this waiting time that Earth-based observatories are expected to discover one or more potential targets for the mission, if one was not found prior to launch. Following target selection, Comet Interceptor will remain near SEL2 waiting for the right conditions to start the transfer towards the target comet.

Quasi-Halo orbits around SEL2, up to an amplitude of ∼1 million km, have a period of ∼180 days. These orbits are inherently unstable; any small perturbation will lead to an exponential deviation from the reference orbit, hence periodic station keeping manoeuvres are planned. The frequency and size of these manoeuvres depends on the spacecraft’s ability to reduce velocity perturbations and dynamic noise. The current plan considers station keeping manoeuvres every 28 days and allocates 2.3 m/s per year to stay at SEL2.

#### Transfer from SEL2 to Encounter

Newly detected potential targets will be monitored continuously from Earth, and the possible transfer trajectories and encounter/post-encounter profiles will be studied in detail for each candidate a minimum of 6 months in advance of initiating the transfer. Once the comet target is selected, or a decision is made to go to a backup, the spacecraft will wait until the optimum time to depart from its orbit around SEL2.

An optimal transfer trajectory avoids costly out-of-plane $\varDelta $V manoeuvres and stays close to the ecliptic plane. As a result, the target comet has to be intercepted at one of its ecliptic nodal crossings, hence the encounter location can be defined by just 2 parameters: the heliocentric distance at encounter $R_{c}$ and the phase angle of Earth at encounter $\theta $, i.e. Comet-Sun-Earth angle. Analysed by 2-body dynamics under solar gravity, the transfer orbit needs to have the perihelion and/or the aphelion distance adjusted in such a way that $R_{c}$ can be reached, together with an orbital period such that the phase drift, ahead or behind Earth, that leads to the desired angle $\theta $ in a given transfer time. Typically, this requires ≤2 trajectory manoeuvres, though in a few cases a third manoeuvre can be beneficial. At the beginning of the transfer, when the spacecraft leaves SEL2, the gravity effect of Earth has a significant impact on the trajectory. Extensive analysis leads to two different strategies being envisaged:

1. *Direct Transfer*. The spacecraft performs a manoeuvre to leave the SEL2 parking orbit in order to exit the gravitational pull of the Earth-Moon system and is injected into a heliocentric orbit drifting towards encounter. A second deep-space manoeuvre, during the transfer orbit to the comet might be necessary to adjust the orbit or the phasing. These transfers can be Exterior, when the spacecraft leaves directly towards the outside of the Sun-Earth direction, or Interior, leaving towards Earth, performing a high-altitude Earth flyby before leaving in the direction of Sun-Earth Lagrange Point 1. The complex dynamics of the interior case can exploit multiple loops around the Earth and/or an Earth flyby to reduce the transfer $\varDelta $V.
2. *Moon Gravity Assist*. The dynamics of the SEL2 manifold towards Earth allow a Moon flyby to be performed, after which the spacecraft can escape from Earth with a velocity at infinity of ∼1–1.4 km/s and in a direction approximately opposite to the Earth’s velocity vector. This is an efficient way to reach heliocentric orbits with perihelion <1 au and favours targets with negative phase angle at encounter ($\theta <0^{\circ}$, ahead of Earth). The Moon flyby allows $\varDelta $V savings, but introduces additional operational complexity.

**Fig. 33:**
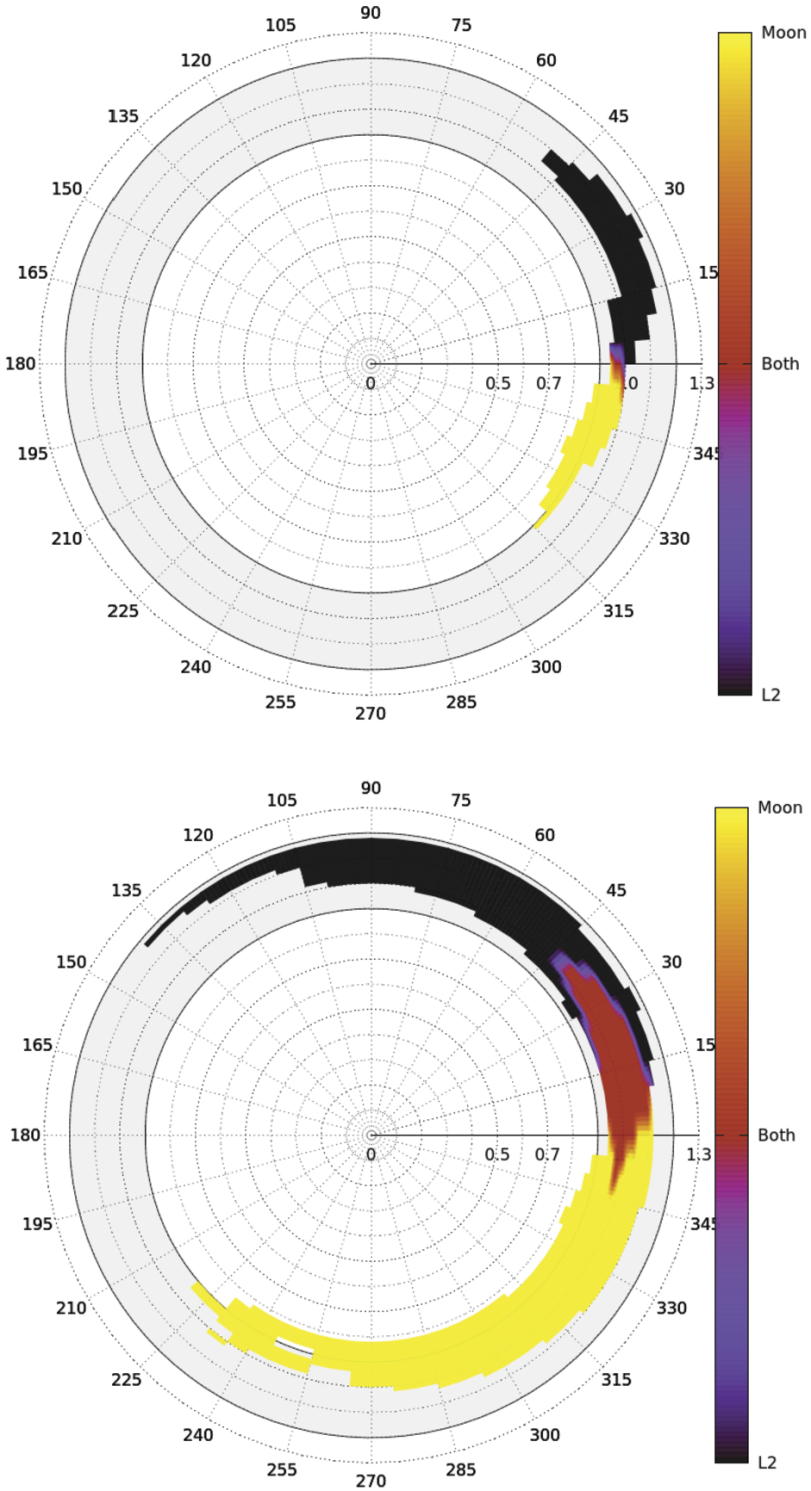
Reachable $R_{c}$-$\theta $ regions with 750 m/s for sample transfer times of 1 year (top) and 3 years (bottom)

The reachable domain of comet encounters for each strategy (Fig. 33), is driven by a trade between transfer time and $\varDelta $V. Direct transfers favour $R_{c}>1$ au and $\theta >0^{\circ}$ (behind Earth), while the opposite occurs for transfers with Moon gravity assist, whereas a region of overlap exists in which both strategies are feasible. Increasing the transfer time significantly impacts the reachable domain.

**Fig. 34:**
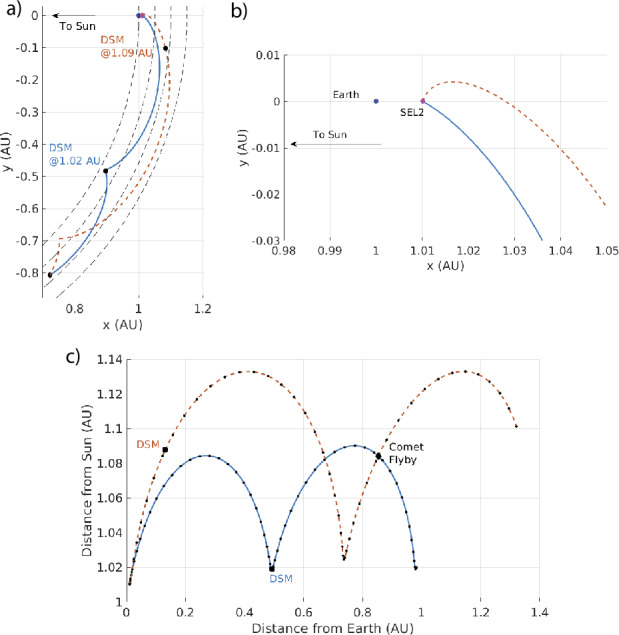
Example geometry of sample transfers to comet 26P, which was an original backup target for the mission. a) Projection on Sun-Earth rotating frame. Solid blue line: minimum $\varDelta $V; dashed orange line: minimum time of flight. b) Zoom of SEL2 departure. c) Distances to Earth and Sun; dots every 15 days; 6 months post-encounter phase

Figure 34 illustrates sample transfer trajectories to an identified backup target. Two transfer trajectories are shown: one optimised for minimum $\varDelta $V, requiring 37 m/s and 847 days, and one optimised for minimum transfer time with a $\varDelta $V cap at 570 m/s that reduces the transfer duration to 529 days. In both cases the exterior direct transfer strategy is used, and the transfer requires 2 manoeuvres, one to depart from SEL2 and one deep space manoeuvre during the cruise towards the comet encounter.

It is likely that Comet Interceptor will be able to slightly adjust its transfer trajectory in order to also encounter a suitable inactive minor body. Such an additional flyby would be a good opportunity for an engineering test of the spacecraft systems and operational procedures required for the comet flyby in a similar scenario, i.e., the optical navigation cameras and autonomous tracking. This would provide valuable experience to increase the mission robustness and probability of success of the actual science flyby.

#### Probabilistic Reachability Analysis and Statistics of Key Mission Parameters

The results presented in this section have been obtained using a Monte Carlo tool that simulates possible Comet Interceptor missions, modelling from the target detection process to the transfer and comet encounter and the following post-encounter phase. The simulations consider a rate of 14 LPCs per year with perihelia <2 au originating from the comet population of Sect. 6.1.1. This underlying assumption is consistent with the historical observations of 21 new LPCs in the 2010–2019 decade with nodal crossings in the accessible 0.9–1.25 au range.

**Fig. 35:**
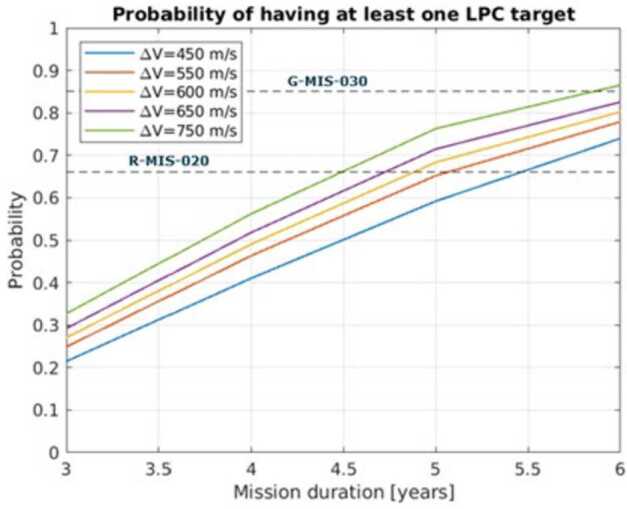
Influence of $\varDelta $V and mission duration on the probability of at least one LPC target. Time between launch and target detection <2 years, mission duration includes 6 months post-encounter phase

The probability of finding at least one feasible LPC target within the given set of constraints and mission requirements is used as a figure of merit. Figure 35 shows how the mission duration and the transfer $\varDelta $V impact this probability. With the allocations at the time of writing of 600 m/s for the transfer and 6 years overall mission duration the probability is 80% (30% of single target plus 50% of multiple targets). Excluding the transfer option with the Moon gravity assist reduces the probability significantly to 63%.

**Fig. 36:**
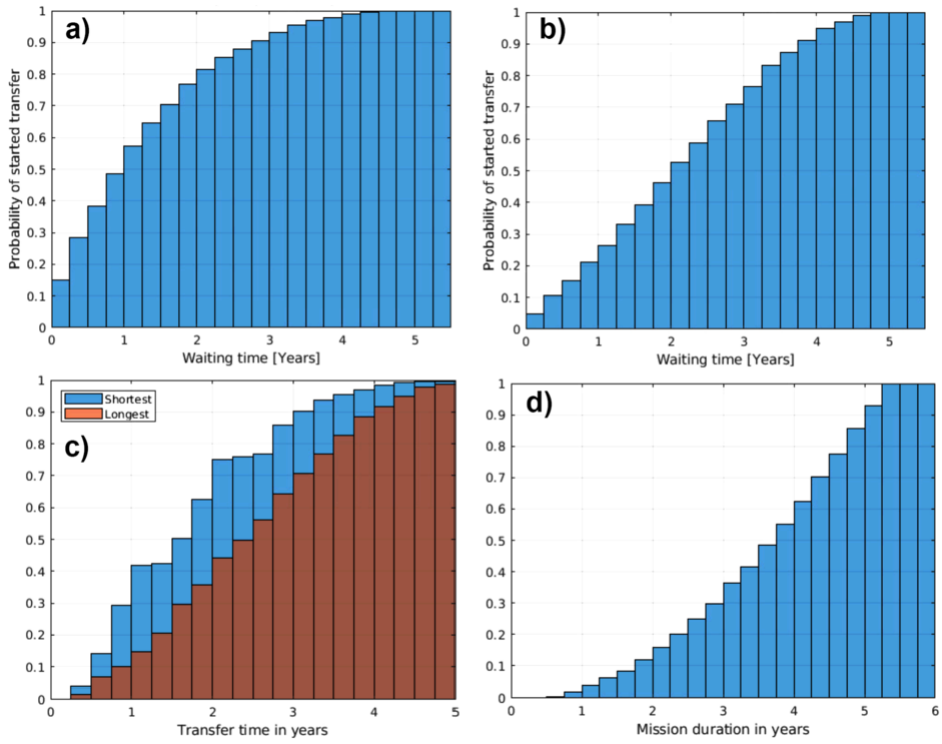
Statistics of time parameters relevant for the mission. a) Shortest wait at SEL2; b) Longest wait at SEL2; c) Transfer time from SEL2 to comet encounter; d) Duration from launch to comet encounter

The Monte Carlo mission simulator provided statistical information on durations relevant to the mission. We have to consider that missions finding multiple feasible targets might choose to favour a given parameter, thus two limiting cases are studied. The main results are summarised in Fig. 36 in which the case of the mission eventually intercepting a backup comet target has been disregarded. The median waiting time at SEL2 is 1–2 years, while waiting times >4 years rarely occur (<5% of cases).

For transfer from SEL2 to the comet, when aiming for the shortest transfer there is a preference for heliocentric transfers of durations close to an integer number of years. Aiming for the longest transfer tends to smooth out the peaks and to result in more uniform distribution. The median of this parameter is 1.5–2.5 years.

For the mission duration from launch to comet encounter (limited to 5.5 years assuming 6 years maximum overall duration, including 6 months of post-encounter activities), the statistics show an increasing probability density followed by a flat region for durations >4 years. The median is observed ∼3.5 years and the 90th percentile at ∼5 years.

#### Comet Encounter

**Fig. 37:**
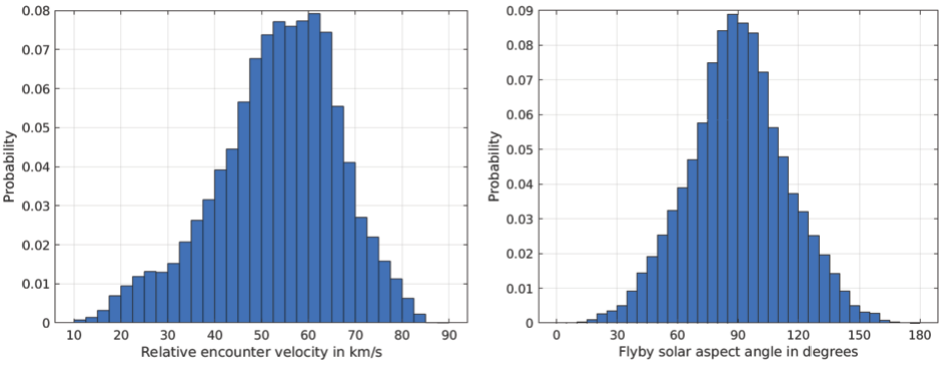
Relative encounter speed (left) and flyby solar aspect angle (right) for reachable LPCs (0.7 au < Rc < 1.3 au)

Figure 37 shows the distribution of the encounter relative speed, and the flyby solar aspect angle (the angle between the Comet-Sun vector and the relative velocity vector), for simulated feasible encounters obtained from the population of LPCs. The relative velocity is biased towards higher values and peaks around 60 km/s. The flyby solar aspect angle shows a symmetrical distribution around $90^{\circ}$. Constraining the encounter parameters has an impact on the availability of targets: having a 60 km/s maximum velocity would remove 33% of possible targets, while the baselined 70 km/s requirement removes only 8.5%. On the other hand, the requirement that constrains the flyby solar aspect angle to $90 \pm 45^{\circ}$ removes ∼7.5% of the targets.

**Fig. 38:**
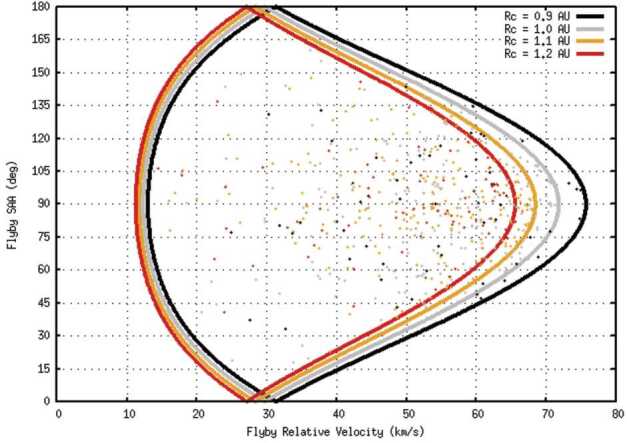
Allowed regions of relative velocities and flyby solar aspect angles

In addition, it must be pointed out that the orbital mechanics of an LPC encounter at a given heliocentric distance, from 0.9 to 1.2 au, constrain the feasible combinations of relative speed and flyby solar aspect angle, as depicted in Fig. 38. The solar aspect angle provides information directly as to whether the encounter is on the inbound or the outbound leg of the comet’s orbit, with the angle being ${>}90^{\circ}$ or ${<}90^{\circ}$, respectively.

The approach to encounter phase is assumed to be defined as the last 60 days before the flyby. By trajectory design there will be no need for a deterministic manoeuvre during this phase, hence operations can focus on the navigation required to reach the comet. This navigation will rely on ground-based measurements of the comet’s position, radio tracking of the spacecraft using the ESTRACK deep space antennas and, most importantly, on the optical data from the navigation cameras (see Sect. 6.1.7). The optical observations have the strength to directly relate the states of the spacecraft and target comet, improving the accuracy of the prediction of the flyby location and time, and allowing critical trajectory correction manoeuvres (TCMs) to be performed.

The flyby targets are defined in the B-plane, i.e. the plane perpendicular to the relative velocity $\overrightarrow{v}_{\mathrm{rel}}$ and passes through the comet nucleus. Two perpendicular directions are defined: the T vector is the orthogonal projection of the Sun-to-target vector onto the B-plane. The R vector completes an orthogonal right-handed triad with S = $\overrightarrow{v}_{\mathrm{rel}}$ and T. The B-plane targets for the spacecraft and probes are defined by the closest approach distance and the angle $\theta $, with the T-axis measured in the direction towards the R-axis.

**Fig. 39:**
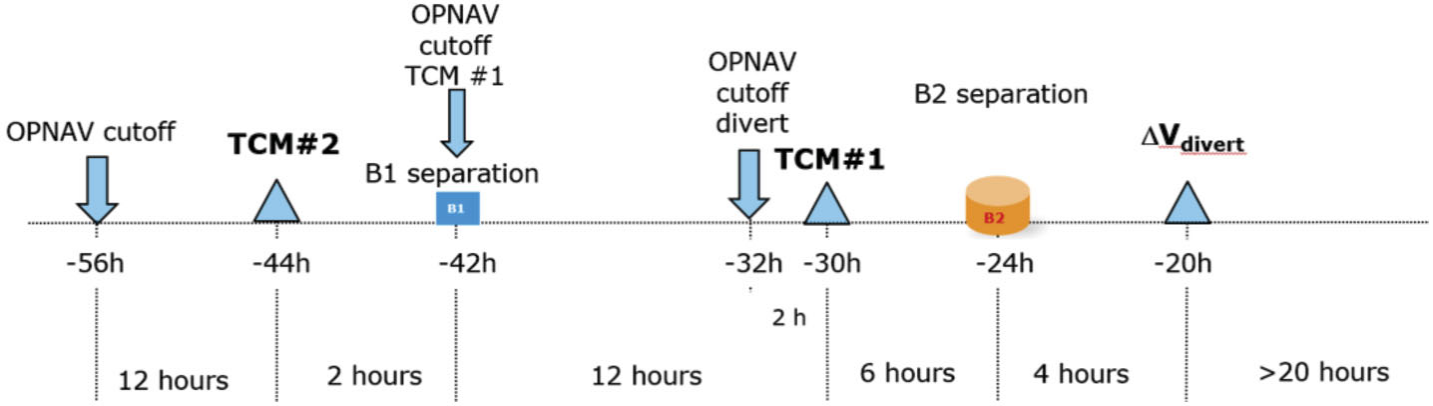
Timeline of operations for the comet flyby

The final approach operations will take place according to the timeline illustrated in Fig. 39, which is still subject of refinement and optimisation in future phases of the mission design.

- The navigation camera will be used continuously to improve the determination of the nucleus position and the flyby accuracy. A ground turn-around time of 12 h (seen as the data cut-off time before each TCM) is considered necessary to downlink the last image, perform the on-ground processing, orbit determination and next manoeuvre and/or separation planning, and to uplink telecommands. Therefore, the input data cut-off for each TCM is 12 hours beforehand.
- The spacecraft composite (A, B1 and B2) is assumed to be targeted at the B1 aim point, with a closest approach of 850 km and $\theta = 135^{\circ}$, thanks to navigation during the approach phase.
- A stochastic TCM at −44 hours to the flyby will target the composite precisely at the B1 aim point, making use of the most updated optical observations.
- Separation of Probe B1 will occur 2 hours later, following a post-TCM tranquilisation phase and a slew to the separation attitude.
- TCM @ 30 hours to the flyby will target the platforms A+B2 towards the aim point of Probe B2 at closest approach of 400 km and $\theta = 180^{\circ}$. This TCM will combine a deterministic part of ∼6 m/s with a stochastic correction.
- Separation of Probe B2 occurs 6 hours later.
- The diversion manoeuvre of spacecraft A occurs −20 hours to the flyby. It targets a greater closest approach distance of 1000 km and $\theta = 180^{\circ}$. The deterministic part of the diversion manoeuvre is 8.5 m/s.

#### Back-up Targets

It is critical for the mission’s success that the availability of a backup target for any launch date is ensured. This has been investigated for a down-selected list of 11 comet candidates provided by the Science Consortium and for the 4-year launch timeframe 2029–2032. The analysis identified 3 backup targets compatible with a transfer $\varDelta $V allocation of 600 m/s and an overall mission duration of 6 years, chronologically as follows:

1. From 2029-03-24 to 2030-05-18: 15P/Finlay
2. From 2030-05-18 to 2030-12-14: 289P/Blanpain
3. From 2030-12-14 onwards: 300P/Catalina

**Fig. 40:**
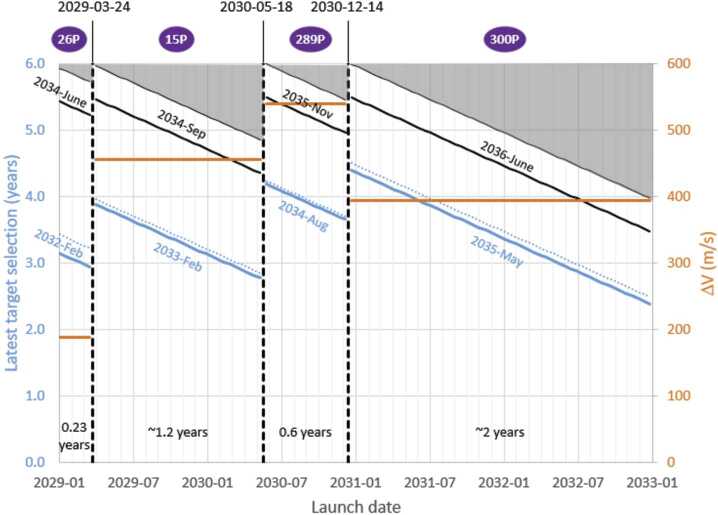
Summary of backup target selected as a function of the launch date. As the launch date at the time of writing is in Q4 of 2029, 26P is no longer reachable

Figure 40 shows a summary of the backup target selection. As an example, for launch on January 1st, 2030, the backup target is 15P/Finlay and the latest selection of a primary LPC target needs to occur before ∼3 years after launch. Otherwise, a transfer to 15P will be used, which requires departure ∼3.5 years after launch and arrives in September 2034; 6 months later, or 5.2 years after launch, the mission will be finished. We observe that more than 3 years are provided consistently from launch until the decision to go to the backup target. Only relatively short periods, in Q1–Q2 2030 and May 2032, provide shorter decision cut-off times of between 2.5 and 3 years.

#### Spacecraft Design Drivers and Schedule

The mission design is driven by the key objective of performing multi-point observations during a high relative velocity flyby with a target which will be identified after the finalisation of the spacecraft design. An Engineering Dust Coma Model, EDCM, was developed by members of the mission’s proposing consortium, to assess the risk from dust particle impacts onto the spacecraft (Marschall et al. 2022), the results of which are available at https://doi.org/10.5281/zenodo.6906815. The main design drivers of spacecraft A and probe B2 are summarised in Table 16.

**Table 16:** Main design drivers for the Comet Interceptor spacecraft

Design driver	Main implications
Dual launch with ARIEL on A62	Max launch mass limited to 975 kg
Multi-point observation principle	Additional probes to be carried by the main spacecraft
Large payload complement	Accommodation of several *in situ* and remote sensing units on spacecraft A and Probe B2
Target defined at late stage	Spacecraft design compatible with range of possible targets, of encounter conditions and Sun-Earth-Target geometries
Maximise probability to reach a suitable target	Maximise *Δ*V capability. Navigation & Target tracking capabilities to remain compatible with multiple targets. High maximum flyby relative velocity (range 10 to 70 km/s)
Interplanetary mission	Spacecraft operating at ∼1–2 au from Earth
Measurements performed during a high relative velocity flyby	“One shot” science. Data downlinked to Earth after the closest approach
Comet environment	Capability to survive the dust environment for a variety of possible targets
Programmatic constraints as from F-Mission call	Cost at completion boundaries. Fast development track. Incompatibility with dedicated technology developments and need to rely on existing, flight qualified solutions

**Fig. 41:**
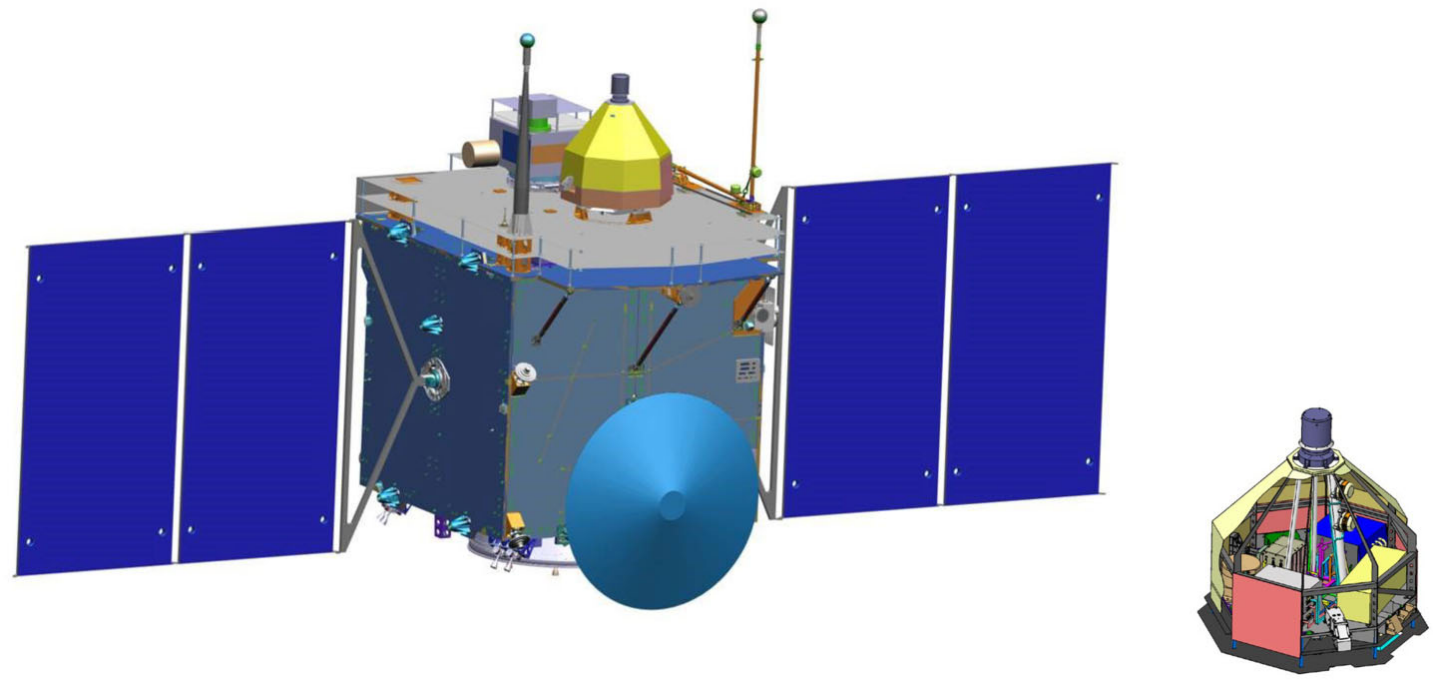
Comet Interceptor spacecraft A and probe B2 design concepts (OHB)

Following adoption of the mission by ESA, the project team carried out the satellite level Preliminary Design Review (PDR). For the mission’s definition phase, two consortia were contracted to provide designs for spacecraft A and probe B2; these were led by OHB-IT and a TAS-UK. On completion of the PDR, the OHB-IT-led consortium was selected by ESA as the prime contractor. The design proposed by the selected OHB-led consortium is represented in Fig. 41. Given the fast development approach followed by Comet Interceptor, the design solutions had to rely on existing platform heritage, minimizing the need for qualification activities. The total spacecraft mass, including propellant and margins is limited to 975 kg by the presently estimated launcher performance (for a dual launch with Ariel). The overall dimensions of the stowed spacecraft are ∼1600 × 1600 × 1500 mm. The configuration of spacecraft A includes:

- a cuboid shape, hosting all platform equipment and accommodating payload and probes.
- the Attitude, Orbit, Guidance and Navigation Control (AOGNC) subsystem, including different sensors (Coarse Sun Sensor, Gyro, Star Trackers, Navigation Cameras) and actuators (Reaction Wheels and Reaction Control System).
- a chemical propulsion system with a large $\varDelta $V capability.
- a communication system based on a fixed High Gain Antenna operating in X band and including an inter-satellite link operating in S band.
- two deployable solar arrays.
- a thermal control system, based on a classic passive design.
- a dust shield protecting spacecraft A and probes during the encounter phase on the ram face.
- two probes accommodated on the same spacecraft side.

The chemical propulsion system is designed to provide, within the maximum allowed launch mass, a minimum transfer $\varDelta $V capability of 600 ms^−1^ (see Sect. 6.1.4); in addition, should the Ariane 6.2 performance improve by the time of Comet Interceptor’s launch, the capability to load additional propellant, thus exceeding the minimum required $\varDelta $V performance, is requested. It is noted that during the flyby the target is maintained in the field of view of the high-resolution camera CoCa via the dedicated Rotating Mirror Assembly (Sect. 5.1), while the spacecraft maintains inertial attitude, maintaining the dust shield in the relative velocity direction.

Probes B1 and B2 are both smaller craft, without propulsion capability, and are deployed from spacecraft A via dedicated separation systems. Both B1 and B2 carry dust shields for protection during the encounter, and will transmit data from their scientific instruments to spacecraft A via dedicated S-band inter-satellite links. The masses of both probes are ∼35 kg each, with a typical diameter/size of ∼0.5 m.

**Fig. 42:**
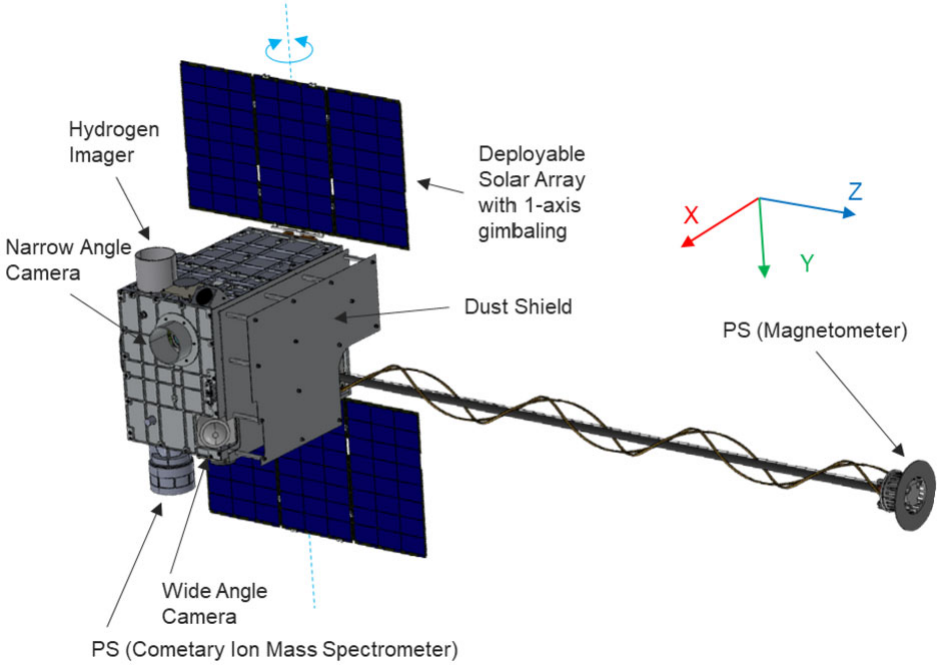
Comet Interceptor probe B1 after separation from spacecraft A (JAXA)

B1 has a cuboid shape, and is 3-axis stabilised through a dedicated Attitude, Guidance and navigation sub-system (Fig. 42). Its electrical power system is based on deployable solar arrays and a secondary battery. B2 has an axisymmetric shape, is gyroscopically stabilised and has no attitude control capability. Its power system based on a primary battery, with an operational lifetime of ∼30 hours.

The key dates for the Comet Interceptor high level schedule are given in Table 17. Compared to L- or M-class missions, the schedule is highly compressed, with 3 years of study phase (0, A, B) from ESA Science Programme Committee selection in June 2019 to formal adoption in June 2022, and 6 years of development phase (C, D) from adoption to launch readiness mid-2028.

**Table 17:** The key dates for the Comet Interceptor high level schedule

Milestone	Date
Selection as F1 mission	June 2019
PDR	Q2 2022
Mission Adoption	June 2022
Prime selection	Q4 2022
Start of phase C/D	Jan 2023
CDR	July 2023 (Instruments)
	Q4 2024 (System)
Delivery of payload flight units	Q4 2025 (probe B2)
	Q1 2026 (spacecraft A)
	Q1 2026 (probe B1)
QAR	Dec. 2027
Launch ready	Mid-2028
Launch (L)	Dec. 2029 (shared with ARIEL)
Arrival at L2	L + 4 months
Waiting at L2 Transfer to target Comet	Maximum 5 years
Comet flyby	Latest L + 5.5 years
End of Operations	Latest L + 6 years

## Conclusion

Comet Interceptor, which will be the first dedicated comet mission launched by any space agency for over 25 years, promises to provide unique, multi-point measurements of a long-period comet, preferably dynamically-new. The mission also represents a new approach to a science mission by ESA, being the first in the agency’s F-class of projects. The mission addresses ambitious science objectives in a modest-cost project, and is the first planetary mission selected by an agency where its primary science target has not been identified beforehand, and may not even be known at the time of launch.

## References

[CR1] Adams FC (2010). The birth environment of the solar system. Annu Rev Astron Astrophys.

[CR2] A’Hearn MF, Millis RC, Schleicher DO, Osip DJ, Birch PV (1995). The ensemble properties of comets: results from narrowband photometry of 85 comets, 1976-1992. Icarus.

[CR3] A’Hearn MF, Belton MJS, Delamere WA, Kissel J, Klaasen KP, McFadden LA, Meech KJ, Melosh HJ, Schultz PH, Sunshine JM, Thomas PC, Veverka J, Yeomans DK, Baca MW, Busko I, Crockett CJ, Collins SM, Desnoyer M, Eberhardy CA, Ernst CM, Farnham TL, Feaga L, Groussin O, Hampton D, Ipatov SI, Li JY, Lindler D, Lisse CM, Mastrodemos N, Owen WM, Richardson JE, Wellnitz DD, White RL (2005). Deep impact: excavating comet Tempel 1. Science.

[CR4] A’Hearn MF, Belton MJS, Delamere WA, Feaga LM, Hampton D, Kissel J, Klaasen KP, McFadden LA, Meech KJ, Melosh HJ, Schultz PH, Sunshine JM, Thomas PC, Veverka J, Wellnitz DD, Yeomans DK, Besse S, Bodewits D, Bowling TJ, Carcich BT, Collins SM, Farnham TL, Groussin O, Hermalyn B, Kelley MS, Kelley MS, Li JY, Lindler DJ, Lisse CM, McLaughlin SA, Merlin F, Protopapa S, Richardson JE, Williams JL (2011). EPOXI at comet Hartley 2. Science.

[CR5] Alfven H (1957). On the theory of comet tails. Tellus.

[CR6] Alho M, Simon Wedlund C, Nilsson H, Kallio E, Jarvinen R, Pulkkinen TI (2019). Hybrid modeling of cometary plasma environments. II. Remote-sensing of a cometary bow shock. Astron Astrophys.

[CR7] Alho M, Jarvinen R, Simon Wedlund C, Nilsson H, Kallio E, Pulkkinen TI (2021). Remote sensing of cometary bow shocks: modelled asymmetric outgassing and pickup ion observations. Mon Not R Astron Soc.

[CR8] Altwegg K, Balsiger H, Bar-Nun A, Berthelier JJ, Bieler A, Bochsler P, Briois C, Calmonte U, Combi M, De Keyser J, Eberhardt P, Fiethe B, Fuselier S, Gasc S, Gombosi TI, Hansen KC, Hässig M, Jäckel A, Kopp E, Korth A, LeRoy L, Mall U, Marty B, Mousis O, Neefs E, Owen T, Rème H, Rubin M, Sémon T, Tzou CY, Waite H, Wurz P (2015). 67P/Churyumov-Gerasimenko, a Jupiter family comet with a high D/H ratio. Science.

[CR9] Altwegg K, Balsiger H, Bar-Nun A, Berthelier JJ, Bieler A, Bochsler P, Briois C, Calmonte U, Combi MR, Cottin H, De Keyser J, Dhooghe F, Fiethe B, Fuselier SA, Gasc S, Gombosi TI, Hansen KC, Haessig M, Jäckel A, Kopp E, Korth A, Le Roy L, Mall U, Marty B, Mousis O, Owen T, Reme H, Rubin M, Semon T, Tzou CY, Waite JH, Wurz P (2016). Prebiotic chemicals–amino acid and phosphorus–in the coma of comet 67P/Churyumov-Gerasimenko. Sci Adv.

[CR10] Altwegg K, Balsiger H, Berthelier JJ, Bieler A, Calmonte U, De Keyser J, Fiethe B, Fuselier SA, Gasc S, Gombosi TI, Owen T, Le Roy L, Rubin M, Sémon T, Tzou CY (2017). D_2_O and HDS in the coma of 67P/Churyumov-Gerasimenko. Philos Trans R Soc Lond Ser A.

[CR11] Altwegg K, Balsiger H, Berthelier JJ, Bieler A, Calmonte U, Fuselier SA, Goesmann F, Gasc S, Gombosi TI, Le Roy L, de Keyser J, Morse A, Rubin M, Schuhmann M, Taylor MGGT, Tzou CY, Wright I (2017). Organics in comet 67P – a first comparative analysis of mass spectra from ROSINA-DFMS, COSAC and Ptolemy. Mon Not R Astron Soc.

[CR12] Altwegg K, Balsiger H, Fuselier SA (2019). Cometary chemistry and the origin of icy solar system bodies: the view after Rosetta. Annu Rev Astron Astrophys.

[CR13] Altwegg K, Balsiger H, Hänni N, Rubin M, Schuhmann M, Schroeder I, Sémon T, Wampfler S, Berthelier JJ, Briois C, Combi M, Gombosi TI, Cottin H, De Keyser J, Dhooghe F, Fiethe B, Fuselier SA (2020). Evidence of ammonium salts in comet 67P as explanation for the nitrogen depletion in cometary comae. Nat Astron.

[CR14] Bagnulo S, Cellino A, Kolokolova L, Nežič R, Santana-Ros T, Borisov G, Christou AA, Bendjoya P, Devogèle M (2021). Unusual polarimetric properties for interstellar comet 2I/Borisov. Nat Commun.

[CR15] Barucci MA, Filacchione G, Fornasier S, Raponi A, Deshapriya JDP, Tosi F, Feller C, Ciarniello M, Sierks H, Capaccioni F, Pommerol A, Massironi M, Oklay N, Merlin F, Vincent JB, Fulchignoni M, Guilbert-Lepoutre A, Perna D, Capria MT, Hasselmann PH, Rousseau B, Barbieri C, Bockelée-Morvan D, Lamy PL, De Sanctis C, Rodrigo R, Erard S, Koschny D, Leyrat C, Rickman H, Drossart P, Keller HU, A’Hearn MF, Arnold G, Bertaux JL, Bertini I, Cerroni P, Cremonese G, Da Deppo V, Davidsson BJR, El-Maarry MR, Fonti S, Fulle M, Groussin O, Güttler C, Hviid SF, Ip W, Jorda L, Kappel D, Knollenberg J, Kramm JR, Kührt E, Küppers M, Lara L, Lazzarin M, Lopez Moreno JJ, Mancarella F, Marzari F, Mottola S, Naletto G, Pajola M, Palomba E, Quirico E, Schmitt B, Thomas N, Tubiana C (2016). Detection of exposed H_2_O ice on the nucleus of comet 67P/Churyumov-Gerasimenko. As observed by Rosetta OSIRIS and VIRTIS instruments. Astron Astrophys.

[CR16] Bauer JM, Grav T, Fernández YR, Mainzer AK, Kramer EA, Masiero JR, Spahr T, Nugent CR, Stevenson RA, Meech KJ, Cutri RM, Lisse CM, Walker R, Dailey JW, Rosser J, Krings P, Ruecker K, Wright EL, NEOWISE Team (2017). Debiasing the NEOWISE cryogenic mission comet populations. Astron J.

[CR17] Bernhardt PA, Roussel-Dupre RA, Pongratz MB, Haerendel G, Valenzuela A, Gurnett DA, Anderson RR (1987). Observations and theory of the AMPTE magnetotail barium releases. J Geophys Res.

[CR18] Bertini I, La Forgia F, Tubiana C, Güttler C, Fulle M, Moreno F, Frattin E, Kovacs G, Pajola M, Sierks H, Barbieri C, Lamy P, Rodrigo R, Koschny D, Rickman H, Keller HU, Agarwal J, A’Hearn MF, Barucci MA, Bertaux JL, Bodewits D, Cremonese G, Da Deppo V, Davidsson B, Debei S, De Cecco M, Drolshagen E, Ferrari S, Ferri F, Fornasier S, Gicquel A, Groussin O, Gutierrez PJ, Hasselmann PH, Hviid SF, Ip WH, Jorda L, Knollenberg J, Kramm JR, Kührt E, Küppers M, Lara LM, Lazzarin M, Lin ZY, Moreno JJL, Lucchetti A, Marzari F, Massironi M, Mottola S, Naletto G, Oklay N, Ott T, Penasa L, Thomas N, Vincent JB (2017). The scattering phase function of comet 67P/Churyumov-Gerasimenko coma as seen from the Rosetta/OSIRIS instrument. Mon Not R Astron Soc.

[CR19] Beth A, Altwegg K, Behar É, Broiles T, Burch J, Carr C, Eriksson A, Galand M, Goetz C, Henri P, Heritier K, Nilsson H, Odelstad E, Richter I, Rubin M, Vallieres X (2017). Ion composition variety and variability around perihelion. EGU general assembly conference abstracts.

[CR20] Beth A, Altwegg K, Balsiger H, Berthelier JJ, Combi CM, De Keyser J, Fiethe B, Fuselier SA, Galand M, Gombosi TI, Rubin M, Sémon T (2020). ROSINA ion zoo at comet 67P. Astron Astrophys.

[CR21] Bieler A, Altwegg K, Balsiger H, Bar-Nun A, Berthelier JJ, Bochsler P, Briois C, Calmonte U, Combi M, de Keyser J, van Dishoeck EF, Fiethe B, Fuselier SA, Gasc S, Gombosi TI, Hansen KC, Hässig M, Jäckel A, Kopp E, Korth A, Le Roy L, Mall U, Maggiolo R, Marty B, Mousis O, Owen T, Rème H, Rubin M, Sémon T, Tzou CY, Waite JH, Walsh C, Wurz P (2015). Abundant molecular oxygen in the coma of comet 67P/Churyumov-Gerasimenko. Nature.

[CR22] Bieler A, Altwegg K, Balsiger H, Berthelier JJ, Calmonte U, Combi M, De Keyser J, Fiethe B, Fougere N, Fuselier S, Gasc S, Gombosi T, Hansen K, Hässig M, Huang Z, Jäckel A, Jia X, Le Roy L, Mall UA, Rème H, Rubin M, Tenishev V, Tóth G, Tzou CY, Wurz P (2015). Comparison of 3D kinetic and hydrodynamic models to ROSINA-COPS measurements of the neutral coma of 67P/Churyumov-Gerasimenko. Astron Astrophys.

[CR23] Biermann L, Brosowski B, Schmidt HU (1967). The interactions of the solar wind with a comet. Sol Phys.

[CR24] Biver N, Bockelée-Morvan D, Paubert G, Moreno R, Crovisier J, Boissier J, Bertrand E, Boussier H, Kugel F, McKay A, Dello Russo N, DiSanti MA (2018). The extraordinary composition of the blue comet C/2016 R2 (PanSTARRS). Astron Astrophys.

[CR25] Blum J, Gundlach B, Krause M, Fulle M, Johansen A, Agarwal J, von Borstel I, Shi X, Hu X, Bentley MS, Capaccioni F, Colangeli L, Della Corte V, Fougere N, Green SF, Ivanovski S, Mannel T, Merouane S, Migliorini A, Rotundi A, Schmied R, Snodgrass C (2017). Evidence for the formation of comet 67P/Churyumov-Gerasimenko through gravitational collapse of a bound clump of pebbles. Mon Not R Astron Soc.

[CR26] Bockelée-Morvan D, Lis DC, Wink JE, Despois D, Crovisier J, Bachiller R, Benford DJ, Biver N, Colom P, Davies JK, Gérard E, Germain B, Houde M, Mehringer D, Moreno R, Paubert G, Phillips TG, Rauer H (2000). New molecules found in comet C/1995 O1 (Hale-Bopp). Investigating the link between cometary and interstellar material. Astron Astrophys.

[CR27] Bodewits D, Lara LM, A’Hearn MF, La Forgia F, Gicquel A, Kovacs G, Knollenberg J, Lazzarin M, Lin ZY, Shi X, Snodgrass C, Tubiana C, Sierks H, Barbieri C, Lamy PL, Rodrigo R, Koschny D, Rickman H, Keller HU, Barucci MA, Bertaux JL, Bertini I, Boudreault S, Cremonese G, Da Deppo V, Davidsson B, Debei S, De Cecco M, Fornasier S, Fulle M, Groussin O, Gutiérrez PJ, Güttler C, Hviid SF, Ip WH, Jorda L, Kramm JR, Kührt E, Küppers M, López-Moreno JJ, Marzari F, Naletto G, Oklay N, Thomas N, Toth I, Vincent JB (2016). Changes in the physical environment of the inner coma of 67P/Churyumov-Gerasimenko with decreasing heliocentric distance. Astron J.

[CR28] Boe B, Jedicke R, Meech KJ, Wiegert P, Weryk RJ, Chambers KC, Denneau L, Kaiser N, Kudritzki RP, Magnier EA, Wainscoat RJ, Waters C (2019). The orbit and size-frequency distribution of long period comets observed by Pan-STARRS1. Icarus.

[CR29] Boehnhardt H, Tozzi GP, Bagnulo S, Muinonen K, Nathues A, Kolokolova L (2008). Photometry and polarimetry of the nucleus of comet 2P/Encke. Astron Astrophys.

[CR30] Boehnhardt H, Bibring JP, Apathy I, Auster HU, Ercoli Finzi A, Goesmann F, Klingelhöfer G, Knapmeyer M, Kofman W, Krüger H, Mottola S, Schmidt W, Seidensticker K, Spohn T, Wright I (2017). The Philae lander mission and science overview. Philos Trans R Soc Lond Ser A.

[CR31] Bowles NE, Ehlmann BL, Klima RL, Blaney D, Calcutt SB, Dickson J, Donaldson Hanna KL, Edwards CS, Evans R, Green R, Frazier W, Greenberger R, House MA, Howe C, Miura J, Pieters C, Sampson M, Schindhelm R, Scheller E, Seybold C, Thompson DR, Troeltzsch J, Warren TJ, Shirley K, Weinberg J (2020). The Lunar Thermal Mapper instrument for the Lunar Trailblazer mission. 51st annual lunar and planetary science conference.

[CR32] Britt DT, Boice DC, Buratti BJ, Campins H, Nelson RM, Oberst J, Sandel BR, Stern SA, Soderblom LA, Thomas N (2004). The morphology and surface processes of comet 19/P Borrelly. Icarus.

[CR33] Brownlee D (2014). The stardust mission: analyzing samples from the edge of the solar system. Annu Rev Earth Planet Sci.

[CR34] Cambianica P, Cremonese G, Fulle M, Simioni E, Naletto G, Pajola M, Lucchetti A, Penasa L, Massironi M, Frattin E, Güttler C, Sierks H, Tubiana C (2021). Long-term measurements of the erosion and accretion of dust deposits on comet 67P/Churyumov-Gerasimenko with the OSIRIS instrument. Mon Not R Astron Soc.

[CR35] Chernova GP, Kiselev NN, Jockers K (1993). Polarimetric characteristics of dust particles as observed in 13 comets: comparisons with asteroids. Icarus.

[CR36] Choi YJ, Cohen M, Merk R, Prialnik D (2002). Long-term evolution of objects in the Kuiper Belt Zone—effects of insolation and radiogenic heating. Icarus.

[CR37] Choukroun M, Keihm S, Schloerb FP, Gulkis S, Lellouch E (2015). Dark side of comet 67P/Churyumov-Gerasimenko in Aug.-Oct. 2014. MIRO/Rosetta continuum observations of polar night in the southern regions. Astron Astrophys.

[CR38] Choukroun M, Altwegg K, Kührt E, Biver N, Bockelée-Morvan D, Drążkowska J, Hérique A, Hilchenbach M, Marschall R, Pätzold M, Taylor MGGT, Thomas N (2020). Dust-to-gas and refractory-to-ice mass ratios of comet 67P/Churyumov-Gerasimenko from Rosetta observations. Space Sci Rev.

[CR39] Ciarniello M, Fulle M, Tosi F, Capaccioni F, Filacchione G, Raponi A, Rinaldi G, Rotundi A, Mottola S, De Sanctis MC, Bockelée-Morvan D, Formisano M, Magni G, Longobardo A, Capria MT (2021). Modeling the seasonal evolution of 67P/Churyumov-Gerasimenko water loss rate. 52nd Lunar and Planetary Science Conference.

[CR40] Ciarniello M, Fulle M, Raponi A, Filacchione G, Capaccioni F, Rotundi A, Rinaldi G, Formisano M, Magni G, Tosi F, De Sanctis MC, Capria MT, Longobardo A, Beck P, Fornasier S, Kappel D, Mennella V, Mottola S, Rousseau B, Arnold G (2022). Macro and micro structures of pebble-made cometary nuclei reconciled by seasonal evolution. Nat Astron.

[CR41] Coates AJ, Mazelle C, Neubauer FM (1997). Bow shock analysis at comets Halley and Grigg-Skjellerup. J Geophys Res.

[CR42] Crews C, Soman M, Allanwood EA, Stefanov K, Leese M, Turner P, Holland A (2020). Quantum efficiency of the CIS115 in a radiation environment. Society of photo-optical instrumentation engineers (SPIE) conference series.

[CR43] Curdt W, Keller HU (1990). Large dust particles along the Giotto trajectory. Icarus.

[CR44] Curdt W, Wilhelm K, Craubner A, Krahn E, Keller HU (1988). Position of comet P/Halley at the Giotto encounter. Astron Astrophys.

[CR45] Davidsson BJR (2021). Thermophysical evolution of planetesimals in the primordial disc. Mon Not R Astron Soc.

[CR46] Davidsson BJR, Gutiérrez PJ, Groussin O, A’Hearn MF, Farnham T, Feaga LM, Kelley MS, Klaasen KP, Merlin F, Protopapa S, Rickman H, Sunshine JM, Thomas PC (2013). Thermal inertia and surface roughness of comet 9P/Tempel 1. Icarus.

[CR47] Davidsson BJR, Sierks H, Güttler C, Marzari F, Pajola M, Rickman H, A’Hearn MF, Auger AT, El-Maarry MR, Fornasier S, Gutiérrez PJ, Keller HU, Massironi M, Snodgrass C, Vincent JB, Barbieri C, Lamy PL, Rodrigo R, Koschny D, Barucci MA, Bertaux JL, Bertini I, Cremonese G, Da Deppo V, Debei S, De Cecco M, Feller C, Fulle M, Groussin O, Hviid SF, Höfner S, Ip WH, Jorda L, Knollenberg J, Kovacs G, Kramm JR, Kührt E, Küppers M, La Forgia F, Lara LM, Lazzarin M, Lopez Moreno JJ, Moissl-Fraund R, Mottola S, Naletto G, Oklay N, Thomas N, Tubiana C (2016). The primordial nucleus of comet 67P/Churyumov-Gerasimenko. Astron Astrophys.

[CR48] De Keyser J, Dhooghe F, Altwegg K, Balsiger H, Berthelier JJ, Briois C, Calmonte U, Cessateur G, Combi MR, Equeter E, Fiethe B, Fuselier S, Gasc S, Gibbons A, Gombosi T, Gunell H, Hässig M, Le Roy L, Maggiolo R, Mall U, Marty B, Neefs E, Rème H, Rubin M, Sémon T, Tzou CY, Wurz P (2017). Evidence for distributed gas sources of hydrogen halides in the coma of comet 67P/Churyumov-Gerasimenko. Mon Not R Astron Soc.

[CR49] De Sanctis MC, Capria MT, Coradini A (2001). Thermal evolution and differentiation of Edgeworth-Kuiper Belt objects. Astron J.

[CR50] De Sanctis MC, Capaccioni F, Ciarniello M, Filacchione G, Formisano M, Mottola S, Raponi A, Tosi F, Bockelée-Morvan D, Erard S, Leyrat C, Schmitt B, Ammannito E, Arnold G, Barucci MA, Combi M, Capria MT, Cerroni P, Ip WH, Kuehrt E, McCord TB, Palomba E, Beck P, Quirico E, Piccioni G, Bellucci G, Fulchignoni M, Jaumann R, Stephan K, Longobardo A, Mennella V, Migliorini A, Benkhoff J, Bibring JP, Blanco A, Blecka M, Carlson R, Carsenty U, Colangeli L, Combes M, Crovisier J, Drossart P, Encrenaz T, Federico C, Fink U, Fonti S, Irwin P, Langevin Y, Magni G, Moroz L, Orofino V, Schade U, Taylor F, Tiphene D, Tozzi GP, Biver N, Bonal L, Combe JP, Despan D, Flamini E, Fornasier S, Frigeri A, Grassi D, Gudipati MS, Mancarella F, Markus K, Merlin F, Orosei R, Rinaldi G, Cartacci M, Cicchetti A, Giuppi S, Hello Y, Henry F, Jacquinod S, Rees JM, Noschese R, Politi R, Peter G, VIRTIS Team (2015). The diurnal cycle of water ice on comet 67P/Churyumov-Gerasimenko. Nature.

[CR51] Della Corte V, Rotundi A, Fulle M, Gruen E, Weissman P, Sordini R, Ferrari M, Ivanovski S, Lucarelli F, Accolla M, Zakharov V, Mazzotta Epifani E, Lopez-Moreno JJ, Rodriguez J, Colangeli L, Palumbo P, Bussoletti E, Crifo JF, Esposito F, Green SF, Lamy PL, McDonnell JAM, Mennella V, Molina A, Morales R, Moreno F, Ortiz JL, Palomba E, Perrin JM, Rietmeijer FJM, Rodrigo R, Zarnecki JC, Cosi M, Giovane F, Gustafson B, Herranz ML, Jeronimo JM, Leese MR, Lopez-Jimenez AC, Altobelli N (2015). GIADA: shining a light on the monitoring of the comet dust production from the nucleus of 67P/Churyumov-Gerasimenko. Astron Astrophys.

[CR52] Della Corte V, Rotundi A, Zakharov V, Ivanovski S, Palumbo P, Fulle M, Longobardo A, Dionnet Z, Liuzzi V, Salatti M (2019). GIADA microbalance measurements on board Rosetta: submicrometer- to micrometer-sized dust particle flux in the coma of comet 67P/Churyumov-Gerasimenko. Astron Astrophys.

[CR53] Della Corte V, Ferretti S, Piccirillo AM, Zakharov V, Di Paolo F, Rotundi A, Ammannito E, Amoroso M, Bertini I, Di Donato P, Ferraioli G, Fiscale S, Fulle M, Inno L, Longobardo A, Mazzotta-Epifani E, Muscari Tomajoli MT, Sindoni G, Tonietti L, Rothkaehl H, Wozniakiewicz PJ, Burchell MJ, Alesbrook LA, Sylvest ME, Patel MR (2023). DISC – the dust impact sensor and counter on-board Comet Interceptor: characterization of the dust coma of a dynamically new comet. Adv Space Res.

[CR54] Dello Russo N, Kawakita H, Vervack RJ, Weaver HA (2016). Emerging trends and a comet taxonomy based on the volatile chemistry measured in thirty comets with high-resolution infrared spectroscopy between 1997 and 2013. Icarus.

[CR55] Dones L, Weissman PR, Levison HF, Duncan MJ, Festou MC, Keller HU, Weaver HA (2004). Oort Cloud formation and dynamics. Comets II.

[CR56] Dones L, Brasser R, Kaib N, Rickman H (2015). Origin and evolution of the cometary reservoirs. Space Sci Rev.

[CR57] Drozdovskaya MN, van Dishoeck EF, Rubin M, Jørgensen JK, Altwegg K (2019). Ingredients for solar-like systems: protostar IRAS 16293-2422 B versus comet 67P/Churyumov-Gerasimenko. Mon Not R Astron Soc.

[CR58] Duncan MJ, Levison HF (1997). A scattered comet disk and the origin of Jupiter family comets. Science.

[CR59] Edberg NJT, Alho M, André M, Andrews DJ, Behar E, Burch JL, Carr CM, Cupido E, Engelhardt IAD, Eriksson AI, Glassmeier KH, Goetz C, Goldstein R, Henri P, Johansson FL, Koenders C, Mandt K, Möstl C, Nilsson H, Odelstad E, Richter I, Simon Wedlund C, Stenberg Wieser G, Szego K, Vigren E, Volwerk M (2016). CME impact on comet 67P/Churyumov-Gerasimenko. Mon Not R Astron Soc.

[CR60] Edberg NJT, Eriksson AI, Odelstad E, Vigren E, Andrews DJ, Johansson F, Burch JL, Carr CM, Cupido E, Glassmeier KH, Goldstein R, Halekas JS, Henri P, Koenders C, Mandt K, Mokashi P, Nemeth Z, Nilsson H, Ramstad R, Richter I, Wieser GS (2016). Solar wind interaction with comet 67P: impacts of corotating interaction regions. J Geophys Res Space Phys.

[CR61] Ekenbäck A, Holmström M, Barabash S, Gunell H (2008). Energetic neutral atom imaging of comets. Geophys Res Lett.

[CR62] El-Maarry MR, Thomas N, Giacomini L, Massironi M, Pajola M, Marschall R, Gracia-Berná A, Sierks H, Barbieri C, Lamy PL, Rodrigo R, Rickman H, Koschny D, Keller HU, Agarwal J, A’Hearn MF, Auger AT, Barucci MA, Bertaux JL, Bertini I, Besse S, Bodewits D, Cremonese G, Da Deppo V, Davidsson B, De Cecco M, Debei S, Güttler C, Fornasier S, Fulle M, Groussin O, Gutierrez PJ, Hviid SF, Ip WH, Jorda L, Knollenberg J, Kovacs G, Kramm JR, Kührt E, Küppers M, La Forgia F, Lara LM, Lazzarin M, Lopez Moreno JJ, Marchi S, Marzari F, Michalik H, Naletto G, Oklay N, Pommerol A, Preusker F, Scholten F, Tubiana C, Vincent JB (2015). Regional surface morphology of comet 67P/Churyumov-Gerasimenko from Rosetta/OSIRIS images. Astron Astrophys.

[CR63] El-Maarry MR, Thomas N, Gracia-Berná A, Pajola M, Lee JC, Massironi M, Davidsson B, Marchi S, Keller HU, Hviid SF, Besse S, Sierks H, Barbieri C, Lamy PL, Koschny D, Rickman H, Rodrigo R, A’Hearn MF, Auger AT, Barucci MA, Bertaux JL, Bertini I, Bodewits D, Cremonese G, Da Deppo V, De Cecco M, Debei S, Güttler C, Fornasier S, Fulle M, Giacomini L, Groussin O, Gutierrez PJ, Ip WH, Jorda L, Knollenberg J, Kovacs G, Kramm JR, Kührt E, Küppers M, Lara LM, Lazzarin M, Lopez Moreno JJ, Marschall R, Marzari F, Naletto G, Oklay N, Pommerol A, Preusker F, Scholten F, Tubiana C, Vincent JB (2016). Regional surface morphology of comet 67P/Churyumov-Gerasimenko from Rosetta/OSIRIS images: the southern hemisphere. Astron Astrophys.

[CR64] El-Maarry MR, Groussin O, Thomas N, Pajola M, Auger AT, Davidsson B, Hu X, Hviid SF, Knollenberg J, Güttler C, Tubiana C, Fornasier S, Feller C, Hasselmann P, Vincent JB, Sierks H, Barbieri C, Lamy P, Rodrigo R, Koschny D, Keller HU, Rickman H, A’Hearn MF, Barucci MA, Bertaux JL, Bertini I, Besse S, Bodewits D, Cremonese G, Da Deppo V, Debei S, De Cecco M, Deller J, Deshapriya JDP, Fulle M, Gutierrez PJ, Hofmann M, Ip WH, Jorda L, Kovacs G, Kramm JR, Kührt E, Küppers M, Lara LM, Lazzarin M, Lin ZY, Lopez Moreno JJ, Marchi S, Marzari F, Mottola S, Naletto G, Oklay N, Pommerol A, Preusker F, Scholten F, Shi X (2017). Surface changes on comet 67P/Churyumov-Gerasimenko suggest a more active past. Science.

[CR65] El-Maarry MR, Groussin O, Keller HU, Thomas N, Vincent JB, Mottola S, Pajola M, Otto K, Herny C, Krasilnikov S (2019). Surface morphology of comets and associated evolutionary processes: a review of Rosetta’s observations of 67P/Churyumov-Gerasimenko. Space Sci Rev.

[CR66] Feaga LM, Protopapa S, Schindhelm R, Stern SA, A’Hearn MF, Bertaux JL, Feldman PD, Parker JW, Steffl AJ, Weaver HA (2015). Far-UV phase dependence and surface characteristics of comet 67P/Churyumov-Gerasimenko as observed with Rosetta Alice. Astron Astrophys.

[CR67] Feldman PD, A’Hearn MF, Bertaux JL, Feaga LM, Parker JW, Schindhelm R, Steffl AJ, Stern SA, Weaver HA, Sierks H, Vincent JB (2015). Measurements of the near-nucleus coma of comet 67P/Churyumov-Gerasimenko with the Alice far-ultraviolet spectrograph on Rosetta. Astron Astrophys.

[CR68] Fernández YR, Kelley MS, Lamy PL, Toth I, Groussin O, Lisse CM, A’Hearn MF, Bauer JM, Campins H, Fitzsimmons A, Licandro J, Lowry SC, Meech KJ, Pittichová J, Reach WT, Snodgrass C, Weaver HA (2013). Thermal properties, sizes, and size distribution of Jupiter-family cometary nuclei. Icarus.

[CR69] Fernández JA, Gallardo T, Young JD (2016). The end states of long-period comets and the origin of Halley-type comets. Mon Not R Astron Soc.

[CR70] Filacchione G, de Sanctis MC, Capaccioni F, Raponi A, Tosi F, Ciarniello M, Cerroni P, Piccioni G, Capria MT, Palomba E, Bellucci G, Erard S, Bockelee-Morvan D, Leyrat C, Arnold G, Barucci MA, Fulchignoni M, Schmitt B, Quirico E, Jaumann R, Stephan K, Longobardo A, Mennella V, Migliorini A, Ammannito E, Benkhoff J, Bibring JP, Blanco A, Blecka MI, Carlson R, Carsenty U, Colangeli L, Combes M, Combi M, Crovisier J, Drossart P, Encrenaz T, Federico C, Fink U, Fonti S, Ip WH, Irwin P, Kuehrt E, Langevin Y, Magni G, McCord T, Moroz L, Mottola S, Orofino V, Schade U, Taylor F, Tiphene D, Tozzi GP, Beck P, Biver N, Bonal L, Combe JP, Despan D, Flamini E, Formisano M, Fornasier S, Frigeri A, Grassi D, Gudipati MS, Kappel D, Mancarella F, Markus K, Merlin F, Orosei R, Rinaldi G, Cartacci M, Cicchetti A, Giuppi S, Hello Y, Henry F, Jacquinod S, Reess JM, Noschese R, Politi R, Peter G (2016). Exposed water ice on the nucleus of comet 67P/Churyumov-Gerasimenko. Nature.

[CR71] Filacchione G, Raponi A, Capaccioni F, Ciarniello M, Tosi F, Capria MT, De Sanctis MC, Migliorini A, Piccioni G, Cerroni P, Barucci MA, Fornasier S, Schmitt B, Quirico E, Erard S, Bockelee-Morvan D, Leyrat C, Arnold G, Mennella V, Ammannito E, Bellucci G, Benkhoff J, Bibring JP, Blanco A, Blecka MI, Carlson R, Carsenty U, Colangeli L, Combes M, Combi M, Crovisier J, Drossart P, Encrenaz T, Federico C, Fink U, Fonti S, Fulchignoni M, Ip WH, Irwin P, Jaumann R, Kuehrt E, Langevin Y, Magni G, McCord T, Moroz L, Mottola S, Palomba E, Schade U, Stephan K, Taylor F, Tiphene D, Tozzi GP, Beck P, Biver N, Bonal L, Combe JP, Despan D, Flamini E, Formisano M, Frigeri A, Grassi D, Gudipati MS, Kappel D, Longobardo A, Mancarella F, Markus K, Merlin F, Orosei R, Rinaldi G, Cartacci M, Cicchetti A, Hello Y, Henry F, Jacquinod S, Reess JM, Noschese R, Politi R, Peter G (2016). Seasonal exposure of carbon dioxide ice on the nucleus of comet 67P/Churyumov-Gerasimenko. Science.

[CR72] Filacchione G, Capaccioni F, Ciarniello M, Raponi A, Rinaldi G, De Sanctis MC, Bockelèe-Morvan D, Erard S, Arnold G, Mennella V, Formisano M, Longobardo A, Mottola S (2020). An orbital water-ice cycle on comet 67P from colour changes. Nature.

[CR73] Fornasier S, Hasselmann PH, Barucci MA, Feller C, Besse S, Leyrat C, Lara L, Gutierrez PJ, Oklay N, Tubiana C, Scholten F, Sierks H, Barbieri C, Lamy PL, Rodrigo R, Koschny D, Rickman H, Keller HU, Agarwal J, A’Hearn MF, Bertaux JL, Bertini I, Cremonese G, Da Deppo V, Davidsson B, Debei S, De Cecco M, Fulle M, Groussin O, Güttler C, Hviid SF, Ip W, Jorda L, Knollenberg J, Kovacs G, Kramm R, Kührt E, Küppers M, La Forgia F, Lazzarin M, Lopez Moreno JJ, Marzari F, Matz KD, Michalik H, Moreno F, Mottola S, Naletto G, Pajola M, Pommerol A, Preusker F, Shi X, Snodgrass C, Thomas N, Vincent JB (2015). Spectrophotometric properties of the nucleus of comet 67P/Churyumov-Gerasimenko from the OSIRIS instrument onboard the ROSETTA spacecraft. Astron Astrophys.

[CR74] Fornasier S, Mottola S, Keller HU, Barucci MA, Davidsson B, Feller C, Deshapriya JDP, Sierks H, Barbieri C, Lamy PL, Rodrigo R, Koschny D, Rickman H, A’Hearn M, Agarwal J, Bertaux JL, Bertini I, Besse S, Cremonese G, Da Deppo V, Debei S, De Cecco M, Deller J, El-Maarry MR, Fulle M, Groussin O, Gutierrez PJ, Güttler C, Hofmann M, Hviid SF, Ip WH, Jorda L, Knollenberg J, Kovacs G, Kramm R, Kührt E, Küppers M, Lara ML, Lazzarin M, Moreno JJL, Marzari F, Massironi M, Naletto G, Oklay N, Pajola M, Pommerol A, Preusker F, Scholten F, Shi X, Thomas N, Toth I, Tubiana C, Vincent JB (2016). Rosetta’s comet 67P/Churyumov-Gerasimenko sheds its dusty mantle to reveal its icy nature. Science.

[CR75] Fornasier S, Hoang HV, Fulle M, Quirico E, Ciarniello M (2023). Volatile exposures on the 67P/Churyumov-Gerasimenko nucleus. Astron Astrophys.

[CR76] Fougere N, Combi MR, Tenishev V, Rubin M, Bonev BP, Mumma MJ (2012). Understanding measured water rotational temperatures and column densities in the very innermost coma of comet 73P/Schwassmann-Wachmann 3 B. Icarus.

[CR77] Fougere N, Combi MR, Rubin M, Tenishev V (2013). Modeling the heterogeneous ice and gas coma of comet 103P/Hartley 2. Icarus.

[CR78] Fulle M (2021). Water and deuterium-to-hydrogen ratio in comets. Mon Not R Astron Soc.

[CR79] Fulle M, Blum J, Green SF, Gundlach B, Herique A, Moreno F, Mottola S, Rotundi A, Snodgrass C (2019). The refractory-to-ice mass ratio in comets. Mon Not R Astron Soc.

[CR80] Fulle M, Blum J, Rotundi A (2020). CO-driven activity constrains the origin of comets. Astron Astrophys.

[CR81] Fulle M, Blum J, Rotundi A, Gundlach B, Güttler C, Zakharov V (2020). How comets work: nucleus erosion versus dehydration. Mon Not R Astron Soc.

[CR82] Furuya K, Aikawa Y (2018). Depletion of heavy nitrogen in the cold gas of star-forming regions. Astrophys J.

[CR83] Fuselier SA, Altwegg K, Balsiger H, Berthelier JJ, Beth A, Bieler A, Briois C, Broiles TW, Burch JL, Calmonte U, Cessateur G, Combi M, De Keyser J, Fiethe B, Galand M, Gasc S, Gombosi TI, Gunell H, Hansen KC, Hässig M, Heritier KL, Korth A, Le Roy L, Luspay-Kuti A, Mall U, Mandt KE, Petrinec SM, Rème H, Rinaldi M, Rubin M, Sémon T, Trattner KJ, Tzou CY, Vigren E, Waite JH, Wurz P (2016). Ion chemistry in the coma of comet 67P near perihelion. Mon Not R Astron Soc.

[CR84] Galand M, Feldman PD, Bockelée-Morvan D, Biver N, Cheng YC, Rinaldi G, Rubin M, Altwegg K, Deca J, Beth A, Stephenson P, Heritier KL, Henri P, Parker JW, Carr C, Eriksson AI, Burch J (2020). Far-ultraviolet aurora identified at comet 67P/Churyumov-Gerasimenko. Nat Astron.

[CR85] Garrod RT (2019). Simulations of ice chemistry in cometary nuclei. Astrophys J.

[CR86] Gerig SB, Marschall R, Thomas N, Bertini I, Bodewits D, Davidsson B, Fulle M, Ip WH, Keller HU, Küppers M, Preusker F, Scholten F, Su CC, Toth I, Tubiana C, Wu JS, Sierks H, Barbieri C, Lamy PL, Rodrigo R, Koschny D, Rickman H, Agarwal J, Barucci MA, Bertaux JL, Cremonese G, Da Deppo V, Debei S, De Cecco M, Deller J, Fornasier S, Groussin O, Gutierrez PJ, Güttler C, Hviid SF, Jorda L, Knollenberg J, Kramm JR, Kührt E, Lara LM, Lazzarin M, Lopez Moreno JJ, Marzari F, Mottola S, Naletto G, Oklay N, Vincent JB (2018). On deviations from free-radial outflow in the inner coma of comet 67P/Churyumov-Gerasimenko. Icarus.

[CR87] Gerig SB, Pinzón-Rodríguez O, Marschall R, Wu JS, Thomas N (2020). Dayside-to-nightside dust coma brightness asymmetry and its implications for nightside activity at comet 67P/Churyumov-Gerasimenko. Icarus.

[CR88] Glassmeier KH (2017). Interaction of the solar wind with comets: a Rosetta perspective. Philos Trans R Soc Lond Ser A.

[CR89] Glassmeier KH, Boehnhardt H, Koschny D, Kührt E, Richter I (2007). The Rosetta Mission: flying towards the origin of the solar system. Space Sci Rev.

[CR90] Goetz C, Koenders C, Richter I, Altwegg K, Burch J, Carr C, Cupido E, Eriksson A, Güttler C, Henri P, Mokashi P, Nemeth Z, Nilsson H, Rubin M, Sierks H, Tsurutani B, Vallat C, Volwerk M, Glassmeier KH (2016). First detection of a diamagnetic cavity at comet 67P/Churyumov-Gerasimenko. Astron Astrophys.

[CR91] Goetz C, Glassmeier KH, Volwerk M, Richter I (2018). Plasma at comet 67P/Churyumov-Gerasimenko: implications for cometary activity. 42nd COSPAR scientific assembly.

[CR92] Groussin O, Sunshine JM, Feaga LM, Jorda L, Thomas PC, Li Y, A’Hearn MF, Belton MJS, Besse S, Carcich B, Farnham TL, Hampton D, Klaasen K, Lisse C, Merlin F, Protopapa S (2013). The temperature, thermal inertia, roughness and color of the nuclei of comets 103P/Hartley 2 and 9P/Tempel 1. Icarus.

[CR93] Groussin O, Attree N, Brouet Y, Ciarletti V, Davidsson B, Filacchione G, Fischer HH, Gundlach B, Knapmeyer M, Knollenberg J, Kokotanekova R, Kührt E, Leyrat C, Marshall D, Pelivan I, Skorov Y, Snodgrass C, Spohn T, Tosi F (2019). The thermal, mechanical, structural, and dielectric properties of cometary nuclei after Rosetta. Space Sci Rev.

[CR94] Gulkis S, Allen M, von Allmen P, Beaudin G, Biver N, Bockelée-Morvan D, Choukroun M, Crovisier J, Davidsson BJR, Encrenaz P, Encrenaz T, Frerking M, Hartogh P, Hofstadter M, Ip WH, Janssen M, Jarchow C, Keihm S, Lee S, Lellouch E, Leyrat C, Rezac L, Schloerb FP, Spilker T (2015). Subsurface properties and early activity of comet 67P/Churyumov-Gerasimenko. Science.

[CR95] Gundlach B, Fulle M, Blum J (2020). On the activity of comets: understanding the gas and dust emission from comet 67/Churyumov-Gerasimenko’s south-pole region during perihelion. Mon Not R Astron Soc.

[CR96] Gunell H, Goetz C, Simon Wedlund C, Lindkvist J, Hamrin M, Nilsson H, Llera K, Eriksson A, Holmström M (2018). The infant bow shock: a new frontier at a weak activity comet. Astron Astrophys.

[CR97] Gunell H, Goetz C, Odelstad E, Beth A, Hamrin M, Henri P, Johansson FL, Nilsson H, Stenberg Wieser G (2021). Ion acoustic waves near a comet nucleus: Rosetta observations at comet 67P/Churyumov-Gerasimenko. Ann Geophys.

[CR98] Güttler C, Mannel T, Rotundi A, Merouane S, Fulle M, Bockelée-Morvan D, Lasue J, Levasseur-Regourd AC, Blum J, Naletto G, Sierks H, Hilchenbach M, Tubiana C, Capaccioni F, Paquette JA, Flandes A, Moreno F, Agarwal J, Bodewits D, Bertini I, Tozzi GP, Hornung K, Langevin Y, Krüger H, Longobardo A, Della Corte V, Tóth I, Filacchione G, Ivanovski SL, Mottola S, Rinaldi G (2019). Synthesis of the morphological description of cometary dust at comet 67P/Churyumov-Gerasimenko. Astron Astrophys.

[CR99] Hadamcik E, Levasseur-Regourd AC, Hines DC, Sen AK, Lasue J, Renard JB (2016). Properties of dust particles in comets from photometric and polarimetric observations of 67P. Mon Not R Astron Soc.

[CR100] Haeberli RM, Altwegg K, Balsiger H, Geiss J (1995). Physics and chemistry of ions in the pile-up region of comet P/Halley. Astron Astrophys.

[CR101] Hajra R, Henri P, Myllys M, Héritier KL, Galand M, Simon Wedlund C, Breuillard H, Behar E, Edberg NJT, Goetz C, Nilsson H, Eriksson AI, Goldstein R, Tsurutani BT, Moré J, Vallières X, Wattieaux G (2018). Cometary plasma response to interplanetary corotating interaction regions during 2016 June-September: a quantitative study by the Rosetta Plasma Consortium. Mon Not R Astron Soc.

[CR102] Hansen KC, Altwegg K, Berthelier JJ, Bieler A, Biver N, Bockelée-Morvan D, Calmonte U, Capaccioni F, Combi MR, de Keyser J, Fiethe B, Fougere N, Fuselier SA, Gasc S, Gombosi TI, Huang Z, Le Roy L, Lee S, Nilsson H, Rubin M, Shou Y, Snodgrass C, Tenishev V, Toth G, Tzou CY, Simon Wedlund C, Rosina Team (2016). Evolution of water production of 67P/Churyumov-Gerasimenko: an empirical model and a multi-instrument study. Mon Not R Astron Soc.

[CR103] Harmon JK, Nolan MC, Giorgini JD, Howell ES (2010). Radar observations of 8P/Tuttle: a contact-binary comet. Icarus.

[CR104] Hartogh P, Lis DC, Bockelée-Morvan D, de Val-Borro M, Biver N, Küppers M, Emprechtinger M, Bergin EA, Crovisier J, Rengel M, Moreno R, Szutowicz S, Blake GA (2011). Ocean-like water in the Jupiter-family comet 103P/Hartley 2. Nature.

[CR105] Hasselmann PH, Barucci MA, Fornasier S, Feller C, Deshapriya JDP, Fulchignoni M, Jost B, Sierks H, Barbieri C, Lamy PL, Rodrigo R, Koschny D, Rickman H, A’Hearn M, Bertaux JL, Bertini I, Cremonese G, Da Deppo V, Davidsson B, Debei S, De Cecco M, Deller J, Fulle M, Gaskell RW, Groussin O, Gutierrez PJ, Güttler C, Hofmann M, Hviid SF, Ip WH, Jorda L, Keller HU, Knollenberg J, Kovacs G, Kramm R, Kührt E, Küppers M, Lara ML, Lazzarin M, Lopez-Moreno JJ, Marzari F, Mottola S, Naletto G, Oklay N, Pommerol A, Thomas N, Tubiana C, Vincent JB (2017). The opposition effect of 67P/Churyumov-Gerasimenko on post-perihelion Rosetta images. Mon Not R Astron Soc.

[CR106] Hässig M, Altwegg K, Balsiger H, Bar-Nun A, Berthelier JJ, Bieler A, Bochsler P, Briois C, Calmonte U, Combi M, De Keyser J, Eberhardt P, Fiethe B, Fuselier SA, Galand M, Gasc S, Gombosi TI, Hansen KC, Jäckel A, Keller HU, Kopp E, Korth A, Kührt E, Le Roy L, Mall U, Marty B, Mousis O, Neefs E, Owen T, Rème H, Rubin M, Sémon T, Tornow C, Tzou CY, Waite JH, Wurz P (2015). Time variability and heterogeneity in the coma of 67P/Churyumov-Gerasimenko. Science.

[CR107] Heritier KL, Altwegg K, Balsiger H, Berthelier JJ, Beth A, Bieler A, Biver N, Calmonte U, Combi MR, De Keyser J, Eriksson AI, Fiethe B, Fougere N, Fuselier SA, Galand M, Gasc S, Gombosi TI, Hansen KC, Hassig M, Kopp E, Odelstad E, Rubin M, Tzou CY, Vigren E, Vuitton V (2017). Ion composition at comet 67P near perihelion: Rosetta observations and model-based interpretation. Mon Not R Astron Soc.

[CR108] Hily-Blant P, Magalhaes V, Kastner J, Faure A, Forveille T, Qi C (2017). Direct evidence of multiple reservoirs of volatile nitrogen in a protosolar nebula analogue. Astron Astrophys.

[CR109] Hoppe P, Rubin M, Altwegg K (2018). Presolar isotopic signatures in meteorites and comets: new insights from the Rosetta mission to comet 67P/Churyumov-Gerasimenko. Space Sci Rev.

[CR110] Huebner WF, Benkhoff J, Capria MT, Coradini A, De Sanctis C, Orosei R, Prialnik D (2006). Heat and gas diffusion in comet nuclei.

[CR111] Jewitt DC, Festou MC, Keller HU, Weaver HA (2004). From cradle to grave: the rise and demise of the comets. Comets II.

[CR112] Jewitt D (2015). Color systematics of comets and related bodies. Astron J.

[CR113] Jewitt D, Kim Y, Mutchler M, Agarwal J, Li J, Weaver H (2021). Cometary activity begins at Kuiper Belt distances: evidence from C/2017 K2. Astron J.

[CR114] Jutzi M, Benz W, Toliou A, Morbidelli A, Brasser R (2017). How primordial is the structure of comet 67P? Combined collisional and dynamical models suggest a late formation. Astron Astrophys.

[CR115] Kameda S, Ozaki M, Enya K, Fuse R, Kouyama T, Sakatani N, Suzuki H, Osada N, Kato H, Miyamoto H, Yamazaki A, Nakamura T, Okamoto T, Ishimaru T, Hong P, Ishibashi K, Takashima T, Ishigami R, Kuo CL, Abe S, Goda Y, Murao H, Fujishima S, Aoyama T, Hagiwara K, Mizumoto S, Tanaka N, Murakami K, Matsumoto M, Tanaka K, Sakuta H (2021). Design of telescopic nadir imager for geomorphology (TENGOO) and observation of surface reflectance by optical chromatic imager (OROCHI) for the Martian Moons Exploration (MMX). Earth Planets Space.

[CR116] Karlsson T, Eriksson AI, Odelstad E, André M, Dickeli G, Kullen A, Lindqvist PA, Nilsson H, Richter I (2017). Rosetta measurements of lower hybrid frequency range electric field oscillations in the plasma environment of comet 67P. Geophys Res Lett.

[CR117] Keihm S, Tosi F, Kamp L, Capaccioni F, Gulkis S, Grassi D, Hofstadter M, Filacchione G, Lee S, Giuppi S, Janssen M, Capria M (2012). Interpretation of combined infrared, submillimeter, and millimeter thermal flux data obtained during the Rosetta fly-by of Asteroid (21) Lutetia. Icarus.

[CR118] Keller HU, Kührt E (2020). Cometary nuclei—from Giotto to Rosetta. Space Sci Rev.

[CR119] Keller HU, Arpigny C, Barbieri C, Bonnet RM, Cazes S, Coradini M, Cosmovici CB, Delamere WA, Huebner WF, Hughes DW, Jamar C, Malaise D, Reitsema HJ, Schmidt HU, Schmidt WKH, Seige P, Whipple FL, Wilhelm K (1986). First Halley multicolour camera imaging results from Giotto. Nature.

[CR120] Keller HU, Delamere WA, Reitsema HJ, Huebner WF, Schmidt HU (1987). Comet P/Halley’s nucleus and its activity. Astron Astrophys.

[CR121] Keller HU, Mottola S, Davidsson B, Schröder SE, Skorov Y, Kührt E, Groussin O, Pajola M, Hviid SF, Preusker F, Scholten F, A’Hearn MF, Sierks H, Barbieri C, Lamy P, Rodrigo R, Koschny D, Rickman H, Barucci MA, Bertaux JL, Bertini I, Cremonese G, Da Deppo V, Debei S, De Cecco M, Fornasier S, Fulle M, Gutiérrez PJ, Ip WH, Jorda L, Knollenberg J, Kramm JR, Küppers M, Lara LM, Lazzarin M, Lopez Moreno JJ, Marzari F, Michalik H, Naletto G, Sabau L, Thomas N, Vincent JB, Wenzel KP, Agarwal J, Güttler C, Oklay N, Tubiana C (2015). Insolation, erosion, and morphology of comet 67P/Churyumov-Gerasimenko. Astron Astrophys.

[CR122] Kelley MSP, Woodward CE, Gehrz RD, Reach WT, Harker DE (2017). Mid-infrared spectra of comet nuclei. Icarus.

[CR123] Kimura H, Hilchenbach M, Merouane S, Paquette J, Stenzel O (2020). The morphological, elastic, and electric properties of dust aggregates in comets: a close look at COSIMA/Rosetta’s data on dust in comet 67P/Churyumov-Gerasimenko. Planet Space Sci.

[CR124] Kiselev N, Rosenbush V, Levasseur-Regourd AC, Kolokolova L (2015). Comets. Polarimetry of stars and planetary systems.

[CR125] Knight MM, Kokotanekova R, Samarasinha HF, Levison NH, Meech K, Combi M (2023). Physical properties of comet nuclei from remote observations. Comets III.

[CR126] Koenders C, Glassmeier KH, Richter I, Motschmann U, Rubin M (2013). Revisiting cometary bow shock positions. Planet Space Sci.

[CR127] Koenders C, Perschke C, Goetz C, Richter I, Motschmann U, Glassmeier KH (2016). Low-frequency waves at comet 67P/Churyumov-Gerasimenko. Observations compared to numerical simulations. Astron Astrophys.

[CR128] Kohout T, Näsilä A, Tikka T, Granvik M, Kestilä A, Penttilä A, Kuhno J, Muinonen K, Viherkanto K, Kallio E (2018). Feasibility of asteroid exploration using CubeSats-ASPECT case study. Adv Space Res.

[CR129] Kokotanekova R, Snodgrass C, Lacerda P, Green SF, Lowry SC, Fernández YR, Tubiana C, Fitzsimmons A, Hsieh HH (2017). Rotation of cometary nuclei: new light curves and an update of the ensemble properties of Jupiter-family comets. Mon Not R Astron Soc.

[CR130] Kokotanekova R, Snodgrass C, Lacerda P, Green SF, Nikolov P, Bonev T (2018). Implications of the small spin changes measured for large Jupiter-family comet nuclei. Mon Not R Astron Soc.

[CR131] Kuroda D, Ishiguro M, Watanabe M, Akitaya H, Takahashi J, Hasegawa S, Ui T, Kanda Y, Takaki K, Itoh R, Moritani Y, Imai M, Goda S, Takagi Y, Morihana K, Honda S, Arai A, Hanayama H, Nagayama T, Nogami D, Sarugaku Y, Murata K, Morokuma T, Saito Y, Oasa Y, Sekiguchi K, Ji W (2015). Optical and near-infrared polarimetry for a highly dormant comet 209P/LINEAR. Astrophys J.

[CR132] Lamy PL, Toth I, Fernandez YR, Weaver HA, Festou MC, Keller HU, Weaver HA (2004). The sizes, shapes, albedos, and colors of cometary nuclei. Comets II.

[CR133] Langevin Y, Merouane S, Hilchenbach M, Vincendon M, Hornung K, Engrand C, Schulz R, Kissel J, Ryno J (2020). Optical properties of cometary particles collected by COSIMA: assessing the differences between microscopic and macroscopic scales. Planet Space Sci.

[CR134] Lara LM, Lin ZY, Rodrigo R, Ip WH (2011). 67P/Churyumov-Gerasimenko activity evolution during its last perihelion before the Rosetta encounter. Astron Astrophys.

[CR135] Lee S, von Allmen P, Allen M, Beaudin G, Biver N, Bockelée-Morvan D, Choukroun M, Crovisier J, Encrenaz P, Frerking M, Gulkis S, Hartogh P, Hofstadter M, Ip WH, Janssen M, Jarchow C, Keihm S, Lellouch E, Leyrat C, Rezac L, Schloerb FP, Spilker T, Gaskell B, Jorda L, Keller HU, Sierks H (2015). Spatial and diurnal variation of water outgassing on comet 67P/Churyumov-Gerasimenko observed from Rosetta/MIRO in August 2014. Astron Astrophys.

[CR136] Levasseur-Regourd AC, Bertaux JL, Dumont R, Festou M, Giese RH, Giovane F, Lamy P, Le Blanc JM, Llebaria A, Weinberg JL (1986). Optical probing of comet Halley from the Giotto spacecraft. Nature.

[CR137] Levasseur-Regourd AC, Renard JB, Shkuratov Y, Hadamcik E (2015). Laboratory studies. Polarimetry of stars and planetary systems.

[CR138] Levasseur-Regourd AC, Agarwal J, Cottin H, Engrand C, Flynn G, Fulle M, Gombosi T, Langevin Y, Lasue J, Mannel T, Merouane S, Poch O, Thomas N, Westphal A (2018). Cometary dust. Space Sci Rev.

[CR139] Levasseur-Regourd AC, Renard JB, Hadamcik E, Lasue J, Bertini I, Fulle M (2019). Interpretation through experimental simulations of phase functions revealed by Rosetta in 67P/Churyumov-Gerasimenko dust coma. Astron Astrophys.

[CR140] Levison HF, Rettig T, Hahn JM (1996). Comet taxonomy. Completing the inventory of the solar system.

[CR141] Levison HF, Duncan MJ (1997). From the Kuiper Belt to Jupiter-family comets: the spatial distribution of ecliptic comets. Icarus.

[CR142] Levison HF, Duncan MJ, Dones L, Gladman BJ (2006). The scattered disk as a source of Halley-type comets. Icarus.

[CR143] Leyrat C, Blain D (2015). Search for regional variations of thermal and electrical properties of comet 67P/CG probed by MIRO/Rosetta. AAS/division for planetary sciences meeting abstracts #47.

[CR144] Lis DC, Bockelée-Morvan D, Güsten R, Biver N, Stutzki J, Delorme Y, Durán C, Wiesemeyer H, Okada Y (2019). Terrestrial deuterium-to-hydrogen ratio in water in hyperactive comets. Astron Astrophys.

[CR145] Longobardo A, Palomba E, Capaccioni F, Ciarniello M, Tosi F, Mottola S, Moroz LV, Filacchione G, Raponi A, Quirico E, Zinzi A, Capria MT, Bockelee-Morvan D, Erard S, Leyrat C, Rinaldi G, Dirri F (2017). Photometric behaviour of 67P/Churyumov-Gerasimenko and analysis of its pre-perihelion diurnal variations. Mon Not R Astron Soc.

[CR146] Lowry S, Duddy SR, Rozitis B, Green SF, Fitzsimmons A, Snodgrass C, Hsieh HH, Hainaut O (2012). The nucleus of comet 67P/Churyumov-Gerasimenko. A new shape model and thermophysical analysis. Astron Astrophys.

[CR147] Luspay-Kuti A, Mousis O, Lunine JI, Ellinger Y, Pauzat F, Raut U, Bouquet A, Mandt KE, Maggiolo R, Ronnet T, Brugger B, Ozgurel O, Fuselier SA (2018). Origin of molecular oxygen in comets: current knowledge and perspectives. Space Sci Rev.

[CR148] Mall U, Altwegg K, Balsiger H, Bar-Nun A, Berthelier JJ, Bieler A, Bochsler P, Briois C, Calmonte U, Combi MR, Dabrowski B, De Keyser J, Dhooghe F, Fiethe B, Fuselier SA, Galli A, Garnier P, Gasc S, Gombosi TI, Hansen KC, Hässig M, Hoang M, Jäckel A, Kopp E, Korth A, Le Roy L, Magee B, Marty B, Mousis O, Rème H, Rubin M, Sémon T, Tzou CY, Waite JH, Wurz P (2016). High-time resolution in-situ investigation of major cometary volatiles around 67P/C-G at 3.1–2.3 AU measured with ROSINA-RTOF. Astrophys J.

[CR149] Mannel T, Bentley MS, Boakes PD, Jeszenszky H, Ehrenfreund P, Engrand C, Koeberl C, Levasseur-Regourd AC, Romstedt J, Schmied R, Torkar K, Weber I (2019). Dust of comet 67P/Churyumov-Gerasimenko collected by Rosetta/MIDAS: classification and extension to the nanometer scale. Astron Astrophys.

[CR150] Markkanen J, Agarwal J, Väisänen T, Penttilä A, Muinonen K (2018). Interpretation of the phase functions measured by the OSIRIS instrument for comet 67P/Churyumov-Gerasimenko. Astrophys J Lett.

[CR151] Marschall R, Su CC, Liao Y, Thomas N, Altwegg K, Sierks H, Ip WH, Keller HU, Knollenberg J, Kührt E, Lai IL, Rubin M, Skorov Y, Wu JS, Jorda L, Preusker F, Scholten F, Gracia-Berná A, Gicquel A, Naletto G, Shi X, Vincent JB (2016). Modelling observations of the inner gas and dust coma of comet 67P/Churyumov-Gerasimenko using ROSINA/COPS and OSIRIS data: first results. Astron Astrophys.

[CR152] Marschall R, Markkanen J, Gerig SB, Pinzón-Rodríguez O, Thomas N, Wu JS (2020). The dust-to-gas ratio, size distribution, and dust fall-back fraction of comet 67P/Churyumov-Gerasimenko: inferences from linking the optical and dynamical properties of the inner comae. Front Phys.

[CR153] Marschall R, Zakharov V, Tubiana C, Kelley MSP, van Damme CC, Snodgrass C, Jones GH, Ivanovski SL, Postberg F, Della Corte V, Vincent JB, Muñoz O, La Forgia F, Levasseur-Regourd AC (2022). Determining the dust environment of an unknown comet for a spacecraft flyby: the case of ESA’s Comet Interceptor mission. Astron Astrophys.

[CR154] Marshall D, Groussin O, Vincent JB, Brouet Y, Kappel D, Arnold G, Capria MT, Filacchione G, Hartogh P, Hofstadter M, Ip WH, Jorda L, Kührt E, Lellouch E, Mottola S, Rezac L, Rodrigo R, Rodionov S, Schloerb P, Thomas N (2018). Thermal inertia and roughness of the nucleus of comet 67P/Churyumov-Gerasimenko from MIRO and VIRTIS observations. Astron Astrophys.

[CR155] Marty B, Avice G, Sano Y, Altwegg K, Balsiger H, Hässig M, Morbidelli A, Mousis O, Rubin M (2016). Origins of volatile elements (H, C, N, noble gases) on Earth and Mars in light of recent results from the ROSETTA cometary mission. Earth Planet Sci Lett.

[CR156] Masoumzadeh N, Oklay N, Kolokolova L, Sierks H, Fornasier S, Barucci MA, Vincent JB, Tubiana C, Güttler C, Preusker F, Scholten F, Mottola S, Hasselmann PH, Feller C, Barbieri C, Lamy PL, Rodrigo R, Koschny D, Rickman H, A’Hearn MF, Bertaux JL, Bertini I, Cremonese G, Da Deppo V, Davidsson BJR, Debei S, De Cecco M, Fulle M, Gicquel A, Groussin O, Gutiérrez PJ, Hall I, Hofmann M, Hviid SF, Ip WH, Jorda L, Keller HU, Knollenberg J, Kovacs G, Kramm JR, Kührt E, Küppers M, Lara LM, Lazzarin M, Lopez Moreno JJ, Marzari F, Naletto G, Shi X, Thomas N (2017). Opposition effect on comet 67P/Churyumov-Gerasimenko using Rosetta-OSIRIS images. Astron Astrophys.

[CR157] Mazelle C, Belmont G, Glassmeier KH, Le Quéau D, Rème H (1991). Ultra low frequency waves at the magnetic pile-up boundary of comet P/Halley. Adv Space Res.

[CR158] McKinnon WB, Richardson DC, Marohnic JC, Keane JT, Grundy WM, Hamilton DP, Nesvorný D, Umurhan OM, Lauer TR, Singer KN, Stern SA, Weaver HA, Spencer JR, Buie MW, Moore JM, Kavelaars JJ, Lisse CM, Mao X, Parker AH, Porter SB, Showalter MR, Olkin CB, Cruikshank DP, Elliott HA, Gladstone GR, Parker JW, Verbiscer AJ, Young LA, New Horizons Science Team (2020). The solar nebula origin of (486958) Arrokoth, a primordial contact binary in the Kuiper Belt. Science.

[CR159] Meech KJ, Svoren J, Festou MC, Keller HU, Weaver HA (2004). Using cometary activity to trace the physical and chemical evolution of cometary nuclei. Comets II.

[CR160] Meech KJ, Kleyna JT, Hainaut O, Micheli M, Bauer J, Denneau L, Keane JV, Stephens H, Jedicke R, Wainscoat R, Weryk R, Flewelling H, Schunová-Lilly E, Magnier E, Chambers KC (2017). CO-driven activity in comet C/2017 K2 (PANSTARRS). Astrophys J Lett.

[CR161] Meier P, Glassmeier KH, Motschmann U (2016). Modified ion-Weibel instability as a possible source of wave activity at comet 67P/Churyumov-Gerasimenko. Ann Geophys.

[CR162] Mennella V, Ciarniello M, Raponi A, Capaccioni F, Filacchione G, Suhasaria T, Popa C, Kappel D, Moroz L, Vinogradoff V, Pommerol A, Rousseau B, Istiqomah I, Bockelee-Morvan D, Carlson RW, Pilorget C (2020). Hydroxylated Mg-rich amorphous silicates: a new component of the 3.2 μm absorption band of comet 67P/Churyumov-Gerasimenko. Astrophys J Lett.

[CR163] Michel P, Küppers M, Bagatin AC, Carry B, Charnoz S, Leon J, Fitzsimmons A, Gordo P, Green SF, Hérique A, Juzi M, Karatekin Ö, Kohout T, Lazzarin M, Murdoch N, Okada T, Palomba E, Pravec P, Snodgrass C, Tortora P, Tsiganis K, Ulamec S, Vincent JB, Wünnemann K, Zhang Y, Raducan SD, Dotto E, Chabot N, Cheng AF, Rivkin A, Barnouin O, Ernst C, Stickle A, Richardson DC, Thomas C, Arakawa M, Miyamoto H, Nakamura A, Sugita S, Yoshikawa M, Abell P, Asphaug E, Ballouz RL, Bottke WF, Lauretta DS, Walsh KJ, Martino P, Carnelli I (2022). The ESA Hera Mission: detailed characterization of the DART impact outcome and of the binary asteroid (65803) Didymos. Planet Sci J.

[CR164] Migliorini A, Piccioni G, Capaccioni F, Filacchione G, Bockelée-Morvan D, Erard S, Leyrat C, Combi MR, Fougere N, Crovisier J, Taylor FW, De Sanctis MC, Capria MT, Grassi D, Rinaldi G, Tozzi GP, Fink U (2016). Water and carbon dioxide distribution in the 67P/Churyumov-Gerasimenko coma from VIRTIS-M infrared observations. Astron Astrophys.

[CR165] Morbidelli A, Nesvorny D, Laurenz V, Marchi S, Rubie DC, Elkins-Tanton L, Wieczorek M, Jacobson S (2018). The timeline of the lunar bombardment: revisited. Icarus.

[CR166] Moreno F, Guirado D, Muñoz O, Bertini I, Tubiana C, Güttler C, Fulle M, Rotundi A, Della Corte V, Ivanovski SL, Rinaldi G, Bockelée-Morvan D, Zakharov VV, Agarwal J, Mottola S, Toth I, Frattin E, Lara LM, Gutiérrez PJ, Lin ZY, Kolokolova L, Sierks H, Naletto G, Lamy PL, Rodrigo R, Koschny D, Davidsson B, Barucci MA, Bertaux JL, Bodewits D, Cremonese G, Da Deppo V, Debei S, De Cecco M, Deller J, Fornasier S, Ip WH, Keller HU, Lazzarin M, López-Moreno JJ, Marzari F, Shi X (2018). Models of Rosetta/OSIRIS 67P dust coma phase function. Astron J.

[CR167] Muñoz O, Moreno F, Gómez-Martín JC, Vargas-Martín F, Guirado D, Ramos JL, Bustamante I, Bertini I, Frattin E, Markannen J, Tubiana C, Fulle M, Güttler C, Sierks H, Rotundi A, Della Corte V, Ivanovski S, Zakharov VV, Bockelée-Morvan D, Blum J, Merouane S, Levasseur-Regourd AC, Kolokolova L, Jardiel T, Caballero AC (2020). Experimental phase function and degree of linear polarization curves of millimeter-sized cosmic dust analogs. Astrophys J Suppl Ser.

[CR168] Myers RV, Nordsieck KH (1984). Spectropolarimetry of comets Austin and Churyumov-Gerasimenko. Icarus.

[CR169] Näsilä A, Kohout T (2020). Miniaturized spectral imaging instrumentation for planetary exploration. 2020 IEEE aerospace conference (AEROCONF 2020), IEEE; AIAA; PHM Soc; IEEE AESS; Cornell Tech Serv; Adv Inst Ind Technol; Motiv; IFT, IEEE aerospace conference proceedings, IEEE aerospace conference, big sky, MT, MAR 06-13, 2020.

[CR170] Nesvorný D, Vokrouhlický D, Dones L, Levison HF, Kaib N, Morbidelli A (2017). Origin and evolution of short-period comets. Astrophys J.

[CR171] Nesvorný D, Vokrouhlický D, Bottke WF, Levison HF (2018). Evidence for very early migration of the solar system planets from the patroclus-menoetius binary Jupiter Trojan. Nat Astron.

[CR172] Neubauer FM (1987). Giotto magnetic-field results on the boundaries of the pile-up region and the magnetic cavity. Astron Astrophys.

[CR173] Neubauer FM, Glassmeier KH, Acuña MH, Mariani F, Musmann G, Ness NF, Coates AJ (1990). Giotto magnetic field observations at the outbound quasi-parallel bow shock of comet Halley. Ann Geophys.

[CR174] Nilsson H, Stenberg Wieser G, Behar E, Simon Wedlund C, Gunell H, Yamauchi M, Lundin R, Barabash S, Wieser M, Carr C, Cupido E, Burch JL, Fedorov A, Sauvaud JA, Koskinen H, Kallio E, Lebreton JP, Eriksson A, Edberg N, Goldstein R, Henri P, Koenders C, Mokashi P, Nemeth Z, Richter I, Szego K, Volwerk M, Vallat C, Rubin M (2015). Birth of a comet magnetosphere: a spring of water ions. Science.

[CR175] Oberst J, Giese B, Howington-Kraus E, Kirk R, Soderblom L, Buratti B, Hicks M, Nelson R, Britt D (2004). The nucleus of comet Borrelly: a study of morphology and surface brightness. Icarus.

[CR176] Odelstad E, Eriksson AI, Johansson FL, Vigren E, Henri P, Gilet N, Heritier KL, Vallières X, Rubin M, André M (2018). Ion velocity and electron temperature inside and around the diamagnetic cavity of comet 67P. J Geophys Res Space Phys.

[CR177] Oort JH (1950). The structure of the cloud of comets surrounding the solar system and a hypothesis concerning its origin. Bull Astron Inst Neth.

[CR178] Parker RJ (2020). The birth environment of planetary systems. R Soc Open Sci.

[CR179] Pfalzner S, Vincke K (2020). Cradle(s) of the Sun. Astrophys J.

[CR180] Pirani S, Johansen A, Bitsch B, Mustill AJ, Turrini D (2019). Consequences of planetary migration on the minor bodies of the early solar system. Astron Astrophys.

[CR181] Pirani S, Johansen A, Mustill AJ (2021). How the formation of Neptune shapes the Kuiper Belt. Astron Astrophys.

[CR182] Poch O, Istiqomah I, Quirico E, Beck P, Schmitt B, Theulé P, Faure A, Hily-Blant P, Bonal L, Raponi A, Ciarniello M, Rousseau B, Potin S, Brissaud O, Flandinet L, Filacchione G, Pommerol A, Thomas N, Kappel D, Mennella V, Moroz L, Vinogradoff V, Arnold G, Erard S, Bockelée-Morvan D, Leyrat C, Capaccioni F, De Sanctis MC, Longobardo A, Mancarella F, Palomba E, Tosi F (2020). Ammonium salts are a reservoir of nitrogen on a cometary nucleus and possibly on some asteroids. Science.

[CR183] Pommerol A, Thomas N, El-Maarry MR, Pajola M, Groussin O, Auger AT, Oklay N, Fornasier S, Feller C, Davidsson B, Gracia-Berná A, Jost B, Marschall R, Poch O, Barucci MA, Bertaux JL, La Forgia F, Keller HU, Kührt E, Lowry SC, Mottola S, Naletto G, Sierks H, Barbieri C, Lamy PL, Rodrigo R, Koschny D, Rickman H, Agarwal J, A’Hearn MF, Bertini I, Boudreault S, Cremonese G, Da Deppo V, De Cecco M, Debei S, Güttler C, Fulle M, Gutierrez PJ, Hviid SF, Ip WH, Jorda L, Knollenberg J, Kovacs G, Kramm JR, Küppers E, Lara L, Lazzarin M, Lopez Moreno JL, Marzari F, Michalik H, Preusker F, Scholten F, Tubiana C, Vincent JB (2015). OSIRIS observations of meter-sized exposures of H_2_O ice at the surface of 67P/Churyumov-Gerasimenko and interpretation using laboratory experiments. Astron Astrophys.

[CR184] Preusker F, Scholten F, Matz KD, Roatsch T, Hviid SF, Mottola S, Knollenberg J, Kührt E, Pajola M, Oklay N, Vincent JB, Davidsson B, A’Hearn MF, Agarwal J, Barbieri C, Barucci MA, Bertaux JL, Bertini I, Cremonese G, Da Deppo V, Debei S, De Cecco M, Fornasier S, Fulle M, Groussin O, Gutiérrez PJ, Güttler C, Ip WH, Jorda L, Keller HU, Koschny D, Kramm JR, Küppers M, Lamy P, Lara LM, Lazzarin M, Lopez Moreno JJ, Marzari F, Massironi M, Naletto G, Rickman H, Rodrigo R, Sierks H, Thomas N, Tubiana C (2017). The global meter-level shape model of comet 67P/Churyumov-Gerasimenko. Astron Astrophys.

[CR185] Quirico E, Moroz LV, Schmitt B, Arnold G, Faure M, Beck P, Bonal L, Ciarniello M, Capaccioni F, Filacchione G, Erard S, Leyrat C, Bockelée-Morvan D, Zinzi A, Palomba E, Drossart P, Tosi F, Capria MT, De Sanctis MC, Raponi A, Fonti S, Mancarella F, Orofino V, Barucci A, Blecka MI, Carlson R, Despan D, Faure A, Fornasier S, Gudipati MS, Longobardo A, Markus K, Mennella V, Merlin F, Piccioni G, Rousseau B, Taylor F (2016). Refractory and semi-volatile organics at the surface of comet 67P/Churyumov-Gerasimenko: insights from the VIRTIS/Rosetta imaging spectrometer. Icarus.

[CR186] Raeder J, Neubauer FM, Ness NF, Burlaga LF (1987). Macroscopic perturbations of the IMF by p/ Halley as seen by the Giotto magnetometer. Astron Astrophys.

[CR187] Raponi A, Ciarniello M, Capaccioni F, Mennella V, Filacchione G, Vinogradoff V, Poch O, Beck P, Quirico E, De Sanctis MC, Moroz LV, Kappel D, Erard S, Bockelée-Morvan D, Longobardo A, Tosi F, Palomba E, Combe JP, Rousseau B, Arnold G, Carlson RW, Pommerol A, Pilorget C, Fornasier S, Bellucci G, Barucci A, Mancarella F, Formisano M, Rinaldi G, Istiqomah I, Leyrat C (2020). Infrared detection of aliphatic organics on a cometary nucleus. Nat Astron.

[CR188] Richter I, Koenders C, Auster HU, Frühauff D, Götz C, Heinisch P, Perschke C, Motschmann U, Stoll B, Altwegg K, Burch J, Carr C, Cupido E, Eriksson A, Henri P, Goldstein R, Lebreton JP, Mokashi P, Nemeth Z, Nilsson H, Rubin M, Szegö K, Tsurutani BT, Vallat C, Volwerk M, Glassmeier KH (2015). Observation of a new type of low-frequency waves at comet 67P/Churyumov-Gerasimenko. Ann Geophys.

[CR189] Rubin M, Altwegg K, van Dishoeck EF, Schwehm G (2015). Molecular oxygen in Oort Cloud comet 1P/Halley. Astrophys J Lett.

[CR190] Rubin M, Engrand C, Snodgrass C, Weissman P, Altwegg K, Busemann H, Morbidelli A, Mumma M (2020). On the origin and evolution of the material in 67P/Churyumov-Gerasimenko. Space Sci Rev.

[CR191] Sánchez JP, Morante D, Hermosin P, Ranuschio D, Estalella A, Viera D, Centuori S, Jones G, Snodgrass C, Levasseur-Regourd AC, Tubiana C (2021). Esa f-class comet interceptor: trajectory design to intercept a yet-to-be-discovered comet. Acta Astronaut.

[CR192] Scarf FL, Ferdinand V, Coroniti V, Kennel CF, Gurnett DA, Ip WH, Smith EJ (1986). Plasma wave observations at comet Giacobini-Zinner. Science.

[CR193] Schloerb FP, Keihm S, von Allmen P, Choukroun M, Lellouch E, Leyrat C, Beaudin G, Biver N, Bockelée-Morvan D, Crovisier J, Encrenaz P, Gaskell R, Gulkis S, Hartogh P, Hofstadter M, Ip WH, Janssen M, Jarchow C, Jorda L, Keller HU, Lee S, Rezac L, Sierks H (2015). MIRO observations of subsurface temperatures of the nucleus of 67P/Churyumov-Gerasimenko. Astron Astrophys.

[CR194] Schwamb ME, Knight MM, Jones GH, Snodgrass C, Bucci L, Sánchez Pérez JM, Skuppin N (2020). Potential backup targets for Comet Interceptor. Res Notes Am Astron Soc.

[CR195] Schwartz SR, Michel P, Jutzi M, Marchi S, Zhang Y, Richardson DC (2018). Catastrophic disruptions as the origin of bilobate comets. Nat Astron.

[CR196] Schwehm G (1991). Giotto extended mission. Adv Space Res.

[CR197] Shirley KA, Warren TJ, Faggi S, Villanueva GL, Protopapa S, Donaldson Hanna KL, Kohut T, Nasila A, Thirumangalath S (2022). Optimizing filter bandpass selection for the Thermal Infrared Imager on ESA’s Comet Interceptor Mission. 53rd lunar and planetary science conference, LPI contributions.

[CR198] Sierks H, Barbieri C, Lamy PL, Rodrigo R, Koschny D, Rickman H, Keller HU, Agarwal J, A’Hearn MF, Angrilli F, Auger AT, Barucci MA, Bertaux JL, Bertini I, Besse S, Bodewits D, Capanna C, Cremonese G, Da Deppo V, Davidsson B, Debei S, De Cecco M, Ferri F, Fornasier S, Fulle M, Gaskell R, Giacomini L, Groussin O, Gutierrez-Marques P, Gutiérrez PJ, Güttler C, Hoekzema N, Hviid SF, Ip WH, Jorda L, Knollenberg J, Kovacs G, Kramm JR, Kührt E, Küppers M, La Forgia F, Lara LM, Lazzarin M, Leyrat C, Lopez Moreno JJ, Magrin S, Marchi S, Marzari F, Massironi M, Michalik H, Moissl R, Mottola S, Naletto G, Oklay N, Pajola M, Pertile M, Preusker F, Sabau L, Scholten F, Snodgrass C, Thomas N, Tubiana C, Vincent JB, Wenzel KP, Zaccariotto M, Pätzold M (2015). On the nucleus structure and activity of comet 67P/Churyumov-Gerasimenko. Science.

[CR199] Simon Wedlund C, Kallio E, Alho M, Nilsson H, Stenberg Wieser G, Gunell H, Behar E, Pusa J, Gronoff G (2016). The atmosphere of comet 67P/Churyumov-Gerasimenko diagnosed by charge-exchanged solar wind alpha particles. Astron Astrophys.

[CR200] Simon Wedlund C, Alho M, Gronoff G, Kallio E, Gunell H, Nilsson H, Lindkvist J, Behar E, Stenberg Wieser G, Miloch WJ (2017). Hybrid modelling of cometary plasma environments. I. Impact of photoionisation, charge exchange, and electron ionisation on bow shock and cometopause at 67P/Churyumov-Gerasimenko. Astron Astrophys.

[CR201] Simon Wedlund C, Behar E, Kallio E, Nilsson H, Alho M, Gunell H, Bodewits D, Beth A, Gronoff G, Hoekstra R (2019). Solar wind charge exchange in cometary atmospheres. II. Analytical model. Astron Astrophys.

[CR202] Simon Wedlund C, Behar E, Nilsson H, Alho M, Kallio E, Gunell H, Bodewits D, Heritier K, Galand M, Beth A, Rubin M, Altwegg K, Volwerk M, Gronoff G, Hoekstra R (2019). Solar wind charge exchange in cometary atmospheres. III. Results from the Rosetta mission to comet 67P/Churyumov-Gerasimenko. Astron Astrophys.

[CR203] Simon Wedlund C, Bodewits D, Alho M, Hoekstra R, Behar E, Gronoff G, Gunell H, Nilsson H, Kallio E, Beth A (2019). Solar wind charge exchange in cometary atmospheres. I. Charge-changing and ionization cross sections for He and H particles in H_2_O. Astron Astrophys.

[CR204] Snodgrass C, Jones GH (2019). The European Space Agency’s Comet Interceptor lies in wait. Nat Commun.

[CR205] Snodgrass C, Fitzsimmons A, Lowry SC, Weissman P (2011). The size distribution of Jupiter family comet nuclei. Mon Not R Astron Soc.

[CR206] Snodgrass C, A’Hearn MF, Aceituno F, Afanasiev V, Bagnulo S, Bauer J, Bergond G, Besse S, Biver N, Bodewits D, Boehnhardt H, Bonev BP, Borisov G, Carry B, Casanova V, Cochran A, Conn BC, Davidsson B, Davies JK, de León J, de Mooij E, de Val-Borro M, Delacruz M, DiSanti MA, Drew JE, Duffard R, Edberg NJT, Faggi S, Feaga L, Fitzsimmons A, Fujiwara H, Gibb EL, Gillon M, Green SF, Guijarro A, Guilbert-Lepoutre A, Gutiérrez PJ, Hadamcik E, Hainaut O, Haque S, Hedrosa R, Hines D, Hopp U, Hoyo F, Hutsemékers D, Hyland M, Ivanova O, Jehin E, Jones GH, Keane JV, Kelley MSP, Kiselev N, Kleyna J, Kluge M, Knight MM, Kokotanekova R, Koschny D, Kramer EA, López-Moreno JJ, Lacerda P, Lara LM, Lasue J, Lehto HJ, Levasseur-Regourd AC, Licandro J, Lin ZY, Lister T, Lowry SC, Mainzer A, Manfroid J, Marchant J, McKay AJ, McNeill A, Meech KJ, Micheli M, Mohammed I, Monguió M, Moreno F, Muñoz O, Mumma MJ, Nikolov P, Opitom C, Ortiz JL, Paganini L, Pajuelo M, Pozuelos FJ, Protopapa S, Pursimo T, Rajkumar B, Ramanjooloo Y, Ramos E, Ries C, Riffeser A, Rosenbush V, Rousselot P, Ryan EL, Santos-Sanz P, Schleicher DG, Schmidt M, Schulz R, Sen AK, Somero A, Sota A, Stinson A, Sunshine JM, Thompson A, Tozzi GP, Tubiana C, Villanueva GL, Wang X, Wooden DH, Yagi M, Yang B, Zaprudin B, Zegmott TJ (2017). The 67P/Churyumov-Gerasimenko observation campaign in support of the Rosetta mission. Philos Trans R Soc Lond Ser A.

[CR207] Stern SA (2003). The evolution of comets in the Oort Cloud and Kuiper Belt. Nature.

[CR208] Stern SA, Shull JM (1988). The influence of supernovae and passing stars on comets in the Oort Cloud. Nature.

[CR209] Sunshine JM, A’Hearn MF, Groussin O, Li JY, Belton MJS, Delamere WA, Kissel J, Klaasen KP, McFadden LA, Meech KJ, Melosh HJ, Schultz PH, Thomas PC, Veverka J, Yeomans DK, Busko IC, Desnoyer M, Farnham TL, Feaga LM, Hampton DL, Lindler DJ, Lisse CM, Wellnitz DD (2006). Exposed water ice deposits on the surface of comet 9P/Tempel 1. Science.

[CR210] Taylor MGGT, Altobelli N, Buratti BJ, Choukroun M (2017). The Rosetta mission orbiter science overview: the comet phase. Philos Trans R Soc Lond Ser A.

[CR211] Thomas N, Keller HU (1989). The colour of comet P/Halley’s nucleus and dust. Astron Astrophys.

[CR212] Thomas PC, A’Hearn MF, Veverka J, Belton MJS, Kissel J, Klaasen KP, McFadden LA, Melosh HJ, Schultz PH, Besse S, Carcich BT, Farnham TL, Groussin O, Hermalyn B, Li JY, Lindler DJ, Lisse CM, Meech K, Richardson JE (2013). Shape, density, and geology of the nucleus of comet 103P/Hartley 2. Icarus.

[CR213] Thomas N, Davidsson B, El-Maarry MR, Fornasier S, Giacomini L, Gracia-Berná AG, Hviid SF, Ip WH, Jorda L, Keller HU, Knollenberg J, Kührt E, La Forgia F, Lai IL, Liao Y, Marschall R, Massironi M, Mottola S, Pajola M, Poch O, Pommerol A, Preusker F, Scholten F, Su CC, Wu JS, Vincent JB, Sierks H, Barbieri C, Lamy PL, Rodrigo R, Koschny D, Rickman H, A’Hearn MF, Barucci MA, Bertaux JL, Bertini I, Cremonese G, Da Deppo V, Debei S, de Cecco M, Fulle M, Groussin O, Gutierrez PJ, Kramm JR, Küppers M, Lara LM, Lazzarin M, Lopez Moreno JJ, Marzari F, Michalik H, Naletto G, Agarwal J, Güttler C, Oklay N, Tubiana C (2015). Redistribution of particles across the nucleus of comet 67P/Churyumov-Gerasimenko. Astron Astrophys.

[CR214] Thomas N, Cremonese G, Ziethe R, Gerber M, Brändli M, Bruno G, Erismann M, Gambicorti L, Gerber T, Ghose K, Gruber M, Gubler P, Mischler H, Jost J, Piazza D, Pommerol A, Rieder M, Roloff V, Servonet A, Trottmann W, Uthaicharoenpong T, Zimmermann C, Vernani D, Johnson M, Pelò E, Weigel T, Viertl J, De Roux N, Lochmatter P, Sutter G, Casciello A, Hausner T, Ficai Veltroni I, Da Deppo V, Orleanski P, Nowosielski W, Zawistowski T, Szalai S, Sodor B, Tulyakov S, Troznai G, Banaskiewicz M, Bridges JC, Byrne S, Debei S, El-Maarry MR, Hauber E, Hansen CJ, Ivanov A, Keszthelyi L, Kirk R, Kuzmin R, Mangold N, Marinangeli L, Markiewicz WJ, Massironi M, McEwen AS, Okubo C, Tornabene LL, Wajer P, Wray JJ (2017). The Colour and Stereo Surface Imaging System (CaSSIS) for the ExoMars Trace Gas Orbiter. Space Sci Rev.

[CR215] Tiscareno MS, Malhotra R (2003). The dynamics of known centaurs. Astron J.

[CR216] Tosi F, Capaccioni F, Capria MT, Mottola S, Zinzi A, Ciarniello M, Filacchione G, Hofstadter M, Fonti S, Formisano M, Kappel D, Kührt E, Leyrat C, Vincent JB, Arnold G, De Sanctis MC, Longobardo A, Palomba E, Raponi A, Rousseau B, Schmitt B, Barucci MA, Bellucci G, Benkhoff J, Bockelée-Morvan D, Cerroni P, Combe JP, Despan D, Erard S, Mancarella F, McCord TB, Migliorini A, Orofino V, Piccioni G (2019). The changing temperature of the nucleus of comet 67P induced by morphological and seasonal effects. Nat Astron.

[CR217] Tremaine S, Dones L (1993). On the statistical distribution of massive impactors. Icarus.

[CR218] Veverka J, Klaasen K, A’Hearn M, Belton M, Brownlee D, Chesley S, Clark B, Economou T, Farquhar R, Green SF, Groussin O, Harris A, Kissel J, Li JY, Meech K, Melosh J, Richardson J, Schultz P, Silen J, Sunshine J, Thomas P, Bhaskaran S, Bodewits D, Carcich B, Cheuvront A, Farnham T, Sackett S, Wellnitz D, Wolf A (2013). Return to comet Tempel 1: overview of Stardust-NExT results. Icarus.

[CR219] Vincent JB (2019). Cometary topography and phase darkening. Astron Astrophys.

[CR220] Vincent JB, Böhnhardt H, Lara LM (2010). A numerical model of cometary dust coma structures. Application to comet 9P/Tempel 1. Astron Astrophys.

[CR221] Vincent JB, Lara LM, Tozzi GP, Lin ZY, Sierks H (2013). Spin and activity of comet 67P/Churyumov-Gerasimenko. Astron Astrophys.

[CR222] Vincent JB, Bodewits D, Besse S, Sierks H, Barbieri C, Lamy P, Rodrigo R, Koschny D, Rickman H, Keller HU, Agarwal J, A’Hearn MF, Auger AT, Barucci MA, Bertaux JL, Bertini I, Capanna C, Cremonese G, da Deppo V, Davidsson B, Debei S, de Cecco M, El-Maarry MR, Ferri F, Fornasier S, Fulle M, Gaskell R, Giacomini L, Groussin O, Guilbert-Lepoutre A, Gutierrez-Marques P, Gutiérrez PJ, Güttler C, Hoekzema N, Höfner S, Hviid SF, Ip WH, Jorda L, Knollenberg J, Kovacs G, Kramm R, Kührt E, Küppers M, La Forgia F, Lara LM, Lazzarin M, Lee V, Leyrat C, Lin ZY, Lopez Moreno JJ, Lowry S, Magrin S, Maquet L, Marchi S, Marzari F, Massironi M, Michalik H, Moissl R, Mottola S, Naletto G, Oklay N, Pajola M, Preusker F, Scholten F, Thomas N, Toth I, Tubiana C (2015). Large heterogeneities in comet 67P as revealed by active pits from sinkhole collapse. Nature.

[CR223] Vincent JB, Oklay N, Pajola M, Höfner S, Sierks H, Hu X, Barbieri C, Lamy PL, Rodrigo R, Koschny D, Rickman H, Keller HU, A’Hearn MF, Barucci MA, Bertaux JL, Bertini I, Besse S, Bodewits D, Cremonese G, Da Deppo V, Davidsson B, Debei S, De Cecco M, El-Maarry MR, Fornasier S, Fulle M, Groussin O, Gutiérrez PJ, Gutiérrez-Marquez P, Güttler C, Hofmann M, Hviid SF, Ip WH, Jorda L, Knollenberg J, Kovacs G, Kramm JR, Kührt E, Küppers M, Lara LM, Lazzarin M, Lin ZY, Lopez Moreno JJ, Lowry S, Marzari F, Massironi M, Moreno F, Mottola S, Naletto G, Preusker F, Scholten F, Shi X, Thomas N, Toth I, Tubiana C (2016). Are fractured cliffs the source of cometary dust jets? Insights from OSIRIS/Rosetta at 67P/Churyumov-Gerasimenko. Astron Astrophys.

[CR224] Volk K, Malhotra R (2008). The scattered disk as the source of the Jupiter family comets. Astrophys J.

[CR225] Volwerk M, Goetz C, Behar E, Delva M, Edberg NJT, Eriksson A, Henri P, Llera K, Nilsson H, Richter I, Stenberg Wieser G, Glassmeier KH (2019). Dynamic field line draping at comet 67P/Churyumov-Gerasimenko during the Rosetta dayside excursion. Astron Astrophys.

[CR226] Wang JH, Brasser R (2014). An Oort Cloud origin of the Halley-type comets. Astron Astrophys.

[CR227] Weibel ES (1959). Spontaneously growing transverse waves in a plasma due to an anisotropic velocity distribution. Phys Rev Lett.

[CR228] Weissman P, Morbidelli A, Davidsson B, Blum J (2020). Origin and evolution of cometary nuclei. Space Sci Rev.

[CR229] Whipple FL (1950). A comet model. I. The acceleration of comet Encke. Astrophys J.

[CR230] Wiegert P, Tremaine S (1999). The evolution of long-period comets. Icarus.

[CR231] Wieser M, Barabash S (2016). A family for miniature, easily reconfigurable particle sensors for space plasma measurements. J Geophys Res Space Phys.

[CR232] Wieser M, Barabash S, Wang XD, Grigoriev A, Zhang A, Wang C, Wang W (2020). The Advanced Small Analyzer for Neutrals (ASAN) on the Chang’E-4 Rover Yutu-2. Space Sci Rev.

[CR233] Wirström ES, Charnley SB (2018). Revised models of interstellar nitrogen isotopic fractionation. Mon Not R Astron Soc.

[CR234] Witasse O (2021). JUICE (Jupiter Icy Moon Explorer): a European mission to explore the emergence of habitable worlds around gas giants. EGU general assembly conference abstracts.

[CR235] Wurz P, Rubin M, Altwegg K, Balsiger H, Berthelier JJ, Bieler A, Calmonte U, De Keyser J, Fiethe B, Fuselier SA, Galli A, Gasc S, Gombosi TI, Jäckel A, Le Roy L, Mall UA, Rème H, Tenishev V, Tzou CY (2015). Solar wind sputtering of dust on the surface of 67P/Churyumov-Gerasimenko. Astron Astrophys.

[CR236] Wyatt MC, Bonsor A, Jackson AP, Marino S, Shannon A (2017). How to design a planetary system for different scattering outcomes: giant impact sweet spot, maximizing exocomets, scattered discs. Mon Not R Astron Soc.

[CR237] Zakharov VV, Ivanovski SL, Crifo JF, Della Corte V, Rotundi A, Fulle M (2018). Asymptotics for spherical particle motion in a spherically expanding flow. Icarus.

